# Insights into the evolutionary history of the most skilled tool-handling platyrrhini monkey: *Sapajus libidinosus* from the Serra da Capivara National Park

**DOI:** 10.1590/1678-4685-GMB-2023-0165

**Published:** 2023-11-10

**Authors:** Thaynara Lima, Bibiana Fam, Gustavo Medina Tavares, Tiago Falótico, Camila Cantele, Lucca Fanti, Luane Landau, Lucas Henriques Viscardi, Pedro Vargas-Pinilla, Ossman Barrientos-Diaz, Alcides Pissinatti, Vinicius A. Sortica, Eduardo B. Ottoni, Ana Lúcia A. Segatto, Andreia Carina Turchetto-Zolet, Maria Cátira Bortolini

**Affiliations:** 1 Universidade Federal do Rio Grande do Sul, Departamento de Genética, Programa de Pós-Graduação em Genética e Biologia Molecular (PPGBM), Instituto de Biociências, Porto Alegre, RS, Brazil.; 2 Universidade de São Paulo, Escola de Artes, Ciências e Humanidades, São Paulo, SP, Brazil.; 3 Universidade Pontifícia Universidade Católica do Rio Grande do Sul, Escola de Medicina, Programa de Pós-Graduação em Medicina e Ciências da Saúde, Porto Alegre, RS, Brazil.; 4 Universidade de São Paulo, Faculdade de Medicina de Ribeirão Preto, Ribeirão Preto, SP, Brazil.; 5 Centro de Primatologia do Rio de Janeiro, Rio de Janeiro, RJ, Brazil.; 6 Universidade de São Paulo, Medicina Preventiva da Faculdade de Medicina, São Paulo, SP, Brazil.; 7 Universidade de São Paulo, Instituto de Psicologia, Departamento de Psicologia Experimental, São Paulo, SP, Brazil.; 8 Universidade Federal de Santa Maria, Centro de Ciências Naturais e Exatas, Departamento de Bioquímica e Biologia Molecular, Santa Maria, RS, Brazil.

**Keywords:** CYTB, diversity, demography, primates

## Abstract

*Sapajus libidinosus* members of the Pedra Furada group, living in the Serra da Capivara National Park, use stone tools in a wider variety of behaviors than any other living animal, except humans. To rescue the evolutionary history of the Caatinga *S. libidinosus* and identify factors that may have contributed to the emergence and maintenance of their tool-use culture, we conducted fieldwork seasons to obtain biological samples of these capuchin monkeys. Using*CYTB*sequences, we show a discrete but constant population growth from the beginning of the Holocene to the present, overlapping the emergence of the Caatinga biome. Our habitat suitability reconstruction reports the presence of plants whose hard fruits, seeds, or roots are processed by capuchins using tools. The*S. libidinosus*individuals in the Caatinga* *were capable of dynamically developing and maintaining their autochthonous culture thanks to: a) cognitive capacity to generate and execute innovation under selective pressure; b) tolerance favoring learning and cultural inheritance; c) an unknown genetic repertoire that underpins the adaptive traits; d) a high degree of terrestriality; e) presence and abundance of natural resources, which makes some places “hot spots” for innovation, and cultural diversification within a relatively short time.

## Introduction

### *Cebus/Sapajus:* Exceptional tool users 

The clade composed of the *Cebus* and *Sapajus* genera (Cebidae family, Platyrrhini suborder) is characterized by a high proportion of brain size relative to their body size, manual dexterity, and exceptional ability to manipulate objects to solve problems in challenging manipulative tasks, both in captive and controlled environments (Reader and Laland, 2002). However, the most notable characteristic of these capuchin monkeys is their relatively sophisticated, innovative, and complex social organization. This includes the use of tools to obtain food and adults who are tolerant of the presence of young learners in a natural environment, allowing the existence of a transgenerational culture (Fragaszy et al., 2004; Ottoni and Izar, 2008; Mannu and Ottoni, 2009; Coelho et al., 2015).

Most studies on tool use by wild populations of capuchin monkeys focus on populations of the *Sapajus* species in the Brazilian Cerrado and Caatinga biomes, which have savanna-like characteristics (Figure S1) (Ottoni and Izar, 2008; Mannu and Ottoni, 2009; Falótico and Ottoni, 2013; Fragaszy et al., 2017; Falótico *et al.*, 2018). These biomes are known for their low and irregular weather patterns that cause periodic droughts, particularly severe in the Caatinga. Some authors prefer to define the Caatinga climate as predominantly semiarid but somewhat unpredictable (Mares et al., 1985; Abreu et al., 2016). 

As a result of the gradual global warming in the early Holocene (12-10 thousand years ago, kya), there was presence of Cerrado vegetation in regions such as the northeastern (NE) Brazil (Mota and Scheel-Ybert, 2019). The Caatinga biome started to emerge from the middle to the late Holocene, about 6-3.2 kya, with the environmental and ecological conditions similar to the current NE Brazilian region (De Oliveira et al., 1999; Pessenda et al., 2010; Mendes, 2016; De Medeiros et al., 2018). 

Nonetheless, the arrival of *Sapajus* species in the Caatinga probably occurred much earlier, in the middle to late Pleistocene (~200 kya; Lima et al., 2017; Martins-Junior et al., 2018), indicating that *Sapajus*and other species were subject to a long selective pressure period in the region. The Caatinga harsh environment imposes challenges for mammals, including primates, as the mammalian fauna is very poor in this biome, with a small number of species at low abundance among tropical arid and semiarid environments (Moura, 2007). For instance, research has shown that the howler monkey *Alouatta caraya* and the marmoset *Callithrix jacchus* have low population densities in the Caatinga, compared to the other biomes these species are distributed. Nevertheless, the average group size of *Sapajus* is within the range reported for Amazonian and Atlantic forests, indicating relative independence from ecological constraints (Moura, 2007). 

*Sapajus libidinosus* individuals have been observed processing endemic cashew nuts (*Anacardium* spp.) and palm nuts (*Astrocarium* spp.*, Attalea* spp.*, Syagrus* spp.), as well as other hard-shelled fruits that are abundant in the Cerrado and the Caatinga (*e.g., Hymenaea* spp., Falótico et al., 2018). They crack the hard-shelled fruits with the help of stone tools, which are used as hammers and anvils (Mannu and Ottoni, 2009). This behavior is exhibited by most individuals over two years of age, with males being the most active tool users when dealing with harder palm nuts. However, no sex differences are observed in the breaking of softer fruits (Ottoni and Izar, 2008). 

Social learning is a significant factor in the traditional and transgenerational use of tools, and essential in establishing a culture (Whitehead et al., 2019). The tolerance of proximity of experienced individuals is a crucial factor that promotes social learning conditions (Coussi-Korbel and Fragaszy, 1995). Adults of *S. libidinosus* exhibit high tolerance, which allows infants and juveniles to observe them and learn tool use as well as the manipulation of objects needed to open encapsulated fruits (Ottoni and Izar, 2008; Mannu and Ottoni, 2009; Coelho et al., 2015). In addition, infants and juveniles scavenge for fresh food obtained from adult fruit processing, providing young apprentices access to a valuable food source. This social tolerance has a high adaptive value in the society of *S. libidinosus* (Ottoni *et al.*, 2005; Ottoni and Izar, 2008). 

### *Sapajus libidinosus* of the Serra da Capivara National Park (SCNP) 

The SCNP *S. libidinosus* individuals routinely use hammers and anvils, as well as stones as “hoes” to dig roots, spider nests, and to cut wood in search of insects and larvae (Mannu and Ottoni, 2009; Falótico et al., 2017). SCNP male monkeys have also been observed using sticks or rods as a “probe” to access prey that nests and hides in small spaces (Ottoni and Izar, 2008; Falótico and Ottoni, 2014). Other innovative behavior has been incorporated into the culture of *S. libidinosus* in the SCNP, such as females throwing stones at males to get their attention (Falótico and Ottoni, 2013).

SCNP is home to numerous capuchin groups. However, most of the exciting results have been described for*S. libidinosus*members of the Boqueirão da Pedra Furada (PF) group (Falótico and Ottoni, 2013, 2014, 2016). According to Falótico *et al.* (2019), Pedra Furada individuals use stone tools extensively and for a wider variety of behaviors more than any other living animal, except for the human species. They are also the most terrestrial platyrrhines group of wild monkeys observed to date, spending around 41% of their daily time on the ground (Falótico and Ottoni, 2023) Despite that, no genetic or transdisciplinary studies have been conducted on *S. libidinosus* individuals in SCNP. Part of the reason for this knowledge gap is the difficulty in accessing these animals, whose level of protection prevents more invasive approaches.

Noteworthy, *Sapajus libidinosus* is a species classified as “Near Threatened” by the Red List of the International Union for Conservation of Nature (IUCN) (Martins et al., 2021) and is at risk of losing 54% of its habitat in the Caatinga in the next 50 years (Moraes et al., 2020) The *S. libidinosus* groups in SCNP are particularly vulnerable, as a decline in population may lead to the end of their tool-using culture (Presotto et al., 2020).

We conducted the first evolutionary, niche modeling, and demographic tests in SCNP/PF group individuals, using mtDNA *CYTB* sequences. Therefore, we predicted the past and present habitat suitability for *S. libidinosus*, *Sapajus nigritus* (a non-tool-using species), and plants whose encapsulated fruits or roots are processed or obtained using tools by SCNP/PF *S. libidinosus* individuals. We hypothesize that using these approaches to retrieve the demographic history of the Caatinga *S. libidinosus* and considering our results in the context of behavioral and archaeological studies, we will be able to identify some of the conditions and factors that may have contributed to the emergence and persistence of this unique (and threatened) culture, which is rare even among primates.

## Material and Methods

### Samples and original genetic data 

The collection of fecal material from 47 individuals belonging to the same social group, named Boqueirão da Pedra Furada (PF), took place during field expeditions organized by one of us (TF) between 2015 and 2019, as well as in 2022, in the Serra da Capivara National Park (SCNP), Piauí State, Northeast Brazil. The PF group size fluctuated during the sampling periods, ranging from 38 to 47 individuals, but the various collection periods allowed us to record the maximum number of animals in the PF group.

Fourteen *Sapajus libidinosus* individuals from Ubajara National Park (UNP, Ceará State, Northeast Brazil) were also sampled and sequenced. The group has a range of 28-30 individuals. In addition, *S. libidinosus* samples from ten individuals living in the Tietê Ecological Park (TEP; São Paulo State, Southeast Brazil) were also sequenced. The founding individuals (two males and three females) were released on the TEP after confiscation by the Brazilian Institute for the Environment and Renewable Natural Resources (IBAMA). The origin of the founder specimens was unknown. Finally, samples of six *Sapajus xanthosternos* from the Serra da Itabaiana National Park (SINP, Sergipe State, Northeast Brazil) were also obtained. They live in a group of around 20-25 monkeys.

SCNP and UNP are located in the Caatinga biome, while SINP is located in the Atlantic Forest biome (Figure S1). *S. libidinosus* living in the SCNP and UNP habitually use stone tools to process hard shell fruits, among other uses, while for this *S. xanthosternos* population there is no record of the use of stones as tools up to date. Other species were included in some analysis for comparison purposes and can be seen in Supplementary material (Table S1).

DNA samples from these capuchins and of other species were extracted using commercial kits according to the instructions of the manufacturer. Original Cytochrome B (*CYTB*) data for these samples were obtained (Table S1). We selected *CYTB* for sequencing because its utility extends beyond reconstructing phylogenetic relationships between taxonomic groups. For instance, Schrago and Mello (2020) estimated the mean CYTB pairwise distances within-species and between-species of primates as 1.5% (0.0-6.7) and 7.3% (2.6-15.9), respectively. This demonstrates that the level of *CYTB* diversity within a primate species can be helpful in population studies.

### Ethics

The current project was registered in three official Brazilian systems: SISBIO (protocol numbers: 48323-1, 05/05/2015; 57039, 09/01/2017; 59019-1; 23/06/2017), SISGEN (protocol number AF00ED5; 27/09/2018) and IBAMA/ICMBio (60134). The Animal Ethics Committee of the involved institutions approved the current project. 

Our approach also complies with the principles for the ethical treatment of non-human primates proposed by the American Society of Primatologists.

### Genetic diversity indices and network analysis

The diversity indices (nucleotide diverstity [π] and haplotype diversity [H]) and genetic structure analysis (*F*_*ST*_ ) were calculated using the *S. libidinosus* CYTB gene dataset, using Arlequin v.3.5.2.2. The DnaSP v.6 program was used to identify the haplotypes of the *S. libidinosus* CYTB sequences and generate data matrices. The hierarchical relationships between haplotypes were observed in haplotype networks constructed in the Haplotype Viewer software, which is based on the implementation of the Phylip package algorithm (phylogeny inference package) that generates a tree from the maximum likelihood method (DNAML). References for the programs cited in this section are presented in the Supplementary material (Materials and Methods).

### Demographic history

A Bayesian Skyline Plot (BSP) was generated using the BEAST v.2.6.7 program to identify possible changes in the effective population size (N_e_; weighted for males and females) over time, based on the*CYTB*data set of*S. libidinosus*. We used a strict clock and a rate of 2x10^-8^ mutations per site per year. The chains were executed for 200 million iterations, from a random starting tree, and sampled every 5,000 generations for the Caatinga biome (including samples from SCNP, UNP, and Genbank sequences) dataset. The first 10% was discarded as burn-in. We tested BSP with the substitution model GTR+I+G, selected according to the results of the jModelTest v.2.1.9 program and present in BEAST 2 package, under the Akaike Information Criterion (AIC). The demographic history over time was reconstructed using Tracer v. 1.7 and Effective Sample Sizes (ESSs) > 200, as indicated by the developers, were checked in the same software.

Another demographic analysis was performed using LAMARC 2.1.10 software. We estimated the molecular diversity parameter theta (Θ) and growth rate (g) to estimate N_e_ and population patterns (expansion, stability, or contraction). Through the Θ parameter we can estimate N_e_ using the formula Θ = Ne × µ (according to the software developers for mtDNA), where µ is the mutation rate in generations. We used the same molecular clock adjusted for generations *(i.e* 1.2 x 10^-7^, considering a generation time of 6 years for *S. libidinosus*. We used a Bayesian search strategy with three replicas, each run for 15,000,000 iterations, sampling every 100 generations, with a 10% burn-in. The best substitution model used was GTR estimated in jModelTest v.2.1.9 from the available models present in the LAMARC software. All ESS values (> 200) were also checked using Tracer v. 1.7.

Finally, Approximate Bayesian Computation (ABC) analyses were conducted to compare scenarios of population stability, bottleneck, expansion, and transitory bottleneck with the BSP N_e_ estimates and LAMARC N_e_ estimates, using DIYABC v. 2.1.0, amounting eight different historical scenarios (Figure S2). This approach allows the choice of the demographic scenario that best-fits the related data to the value of posterior probabilities of the parameters. The posterior probability of each scenario was estimated using logistic regression and direct method. For the best scenario we estimated the analysis of model verification which compares the simulations between prior and posterior distributions and a “real” dataset considering various parameter sets.

We conducted 8,000,000 simulations as indicated by the software and because the GTR model (used in LAMARC runs) is not implemented in the DIYABC software, we used TN93+I+G as the substitution model and the same mutation rate used in BSP and LAMARC. We also conducted a comparison of the best scenario of each model with 5,000,000 simulations and used the median values of current N_e_ (Ne), ancestral N_e_ (Na), and generation time (T) to calculate population patterns (whether growth, stability, or contraction). For all Bayesian analyzes and calculations we used median values and 95% HPD or 95% Credibility Intervals (CI) depending on the software and their methodologies. A complete description of the methods used in the demographic analyzes, as well as the parameters, and priors used in the ABC analyses, and all bioinformatic software references can be found in the Supplementary material (Materials and Methods).

### Times of divergence

The BEAST v.2.6.7 package was used to infer the phylogeny and divergence times based on data from the *CYTB* gene of Platyrrhini primates (Table S1). We performed the Markov Chain Monte Carlo (MCMC) analysis for 100 million iterations and sampled states every 5,000 generations with a random starting tree and relaxed molecular clock log normal, and default distributions considering other parameters. We employed the Yule Method as the prior tree. The substitution model was GTR+I+G, selected according to the program jModelTest v.2.1.10, under the AIC. We evaluated the convergence visually using Tracer v.1.7 to plot probability scores for all parameters by generation time and to check the ESS (>200). A 10% burn-in was used. The Tree Annotator v.2.6.7 (available in the BEAST package) was used to summarize all nodes and the posterior distributions of each parameter in a maximum clade credibility (MCC) tree. The tree was visualized using the FigTree v.1.4.4. More information and the calibration points that were used based on primate fossils can be found in the Supplementary material (Materials and Methods).

### Species distribution modeling

We used species distribution models (SDMs) for*S. libidinosus*and*S. nigritus*, and eight plant species that*S. libidinosus*access as food using tools. We also included*Ficus gomelleria,* a fruit without a hard shell, which Capuchin monkeys also consume. We used both current and past climate data (Middle Holocene: Mid Hol, ~6 kya; Last Glacial Maximum: LGM, ~22 kya; Last Interglacial: LIG, ~140-120 kya) based on 19 bioclimatic variables from the WorldClim database (version 1.4, https://www.worldclim.org/) and CCSM4 model. We retrieved the geospatial occurrences of monkeys and plant species from the Global Biodiversity Information Facility (https://www.gbif.org/, 2021) and SpeciesLink (https://www.macaulaylibrary.org/, 2021). We included location records from the literature and unpublished field data collection by one of us (TF). The occurrences used in modeling for each species were presented in Tables S2-S11. We used a presence-only approach in Maxent v.3.4.4 to create presence SDMs and then project these models to mid Hol, LGM, and LIG climates. To reduce overfitting and improve model performance, we optimized the regularization parameter testing for the optimal regularization value (1 - 3 - 5 - 7 - 9 - 11) using ENMTools version 1.3 (Table S12). Habitat suitability models were evaluated according to the acceptable area under the Receiver Operating Characteristic (ROC) curve values (Area Under the Curve [AUC > 0.7]). More information about the species and bioclimatic variables used can be found in the Supplementary material (Material and Methods).

## Results

### Phylogenetic tree, divergence time and haplotype networks 

Figure S3 and Table S13 demonstrate that *CYTB* provides comparable divergence time estimates to those obtained from genomic data by Martins et al. (2023). This compelling evidence establishes the quality of the original *CYTB* data presented here. The two *S. libidinosus* populations sampled in the Caatinga (SCNP/PF and UNP) may be rescuing a relatively deep coalescing time (~250 kya), connected to the arrival and dispersal of the species in the region during the middle to late Pleistocene. Another possibility is that genetic drift and the founding effect are leading to a level of divergence that can mask the real split time between these two Caatinga subpopulations. 

**Figure 1 -:**
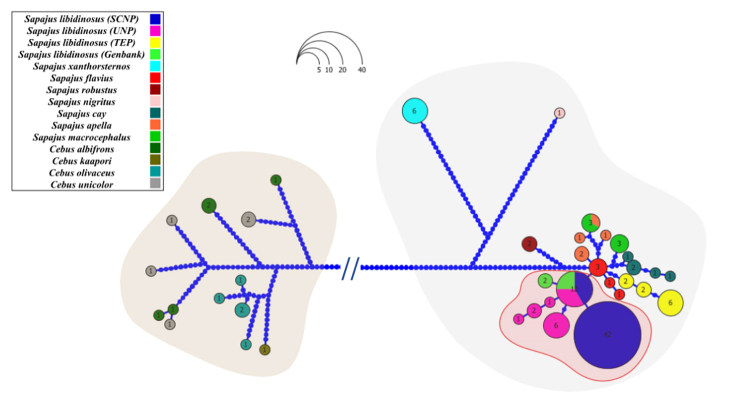
Haplotype network of *Sapajus* and*Cebus*species based on*CYTB*sequences**.** The size of each haplotype circle indicates the number of individuals observed. Each dot on a line connecting two haplotypes indicates a mutational step. The colors correspond to the different species described in the legend. The overlapping shapes indicate the groups, the red indicates the species*S. libidinosus*from the Caatinga, and the gray colors indicate the different genera (*Cebus*and*Sapajus*). The cut bar reduces the size of mutational steps between genera. The intersection of lines can indicate a mean vector interpreted as an unsampled but possibly existing sequence (haplotype) or even extinct ancestral sequences.

The haplotype networks, built using *CYTB* sequences from the *Sapajus* genus, are shown in Figures 1 and Figure S4, with the *Cebus* species used as outgroup. The pattern of not sharing haplotypes within the *Sapajus* genus is remarkable. Two haplotypes were detected in the SCNP/PF *S. libidinosus* group, with the most frequent haplotype being exclusive to that subpopulation and the other haplotype being shared with the Caatinga UNP *S. libidinosus* subpopulation. Notably, when all Caatinga samples are considered, a network with a star-like shape indicates a central haplotype shared by several individuals. Despite not being a classic star shape, this characteristic is often associated with a population that has undergone a bottleneck due to drift or founder effects and subsequent demographic expansion. 

Our *Sapajus xanthosternos* samples indicated that all individuals shared the same *CYTB* haplotype but with many different mutational steps when comparing them with other species of the genus *Sapajus*, corroborating their outermost position in the phylogenetic tree. These animals live in the Atlantic Forest biome but present a transition to Caatinga in the northern area. 

The *F*_*ST*_ value considering SCNP/PF and UNP is 0.69390 (*p*-value = 0.00000+-0.00000; Table S14), indicating that the two natural populations from the Caatinga are significantly different concerning their *CYTB* sequences. These numbers suggest that the SCNP/PF and UNP have old divergence time or experienced genetic drift/founder effect, corroborating our network analysis. In addition, these populations are separated by a relatively large geographical distance, with urbanized areas between them, which also hinders the genetic flow between them. 

### Diversity indices and demographic history

The estimated nucleotide diversity (π) was 0.000181 +/- 0.000257, while the haplotype diversity (H) was 0.1943 +/- 0.0710 for SCNP/PF *S. libidinosus* subpopulation. These values are ~7 times smaller than those obtained for the other investigated Caatinga *S. libidinosus* subpopulation (UNP) and the heterogeneous TEP group (Table S14). Since the diversity of the SCNP/PF population is low, we opted to perform the population demographic analysis considering all available *Sapajus libidinosus* sequences from the Caatinga (H = 0.5604 ± 0.0626; π = 0.001001 +/- 0.000742; Table S14).

**Figure 2 -:**
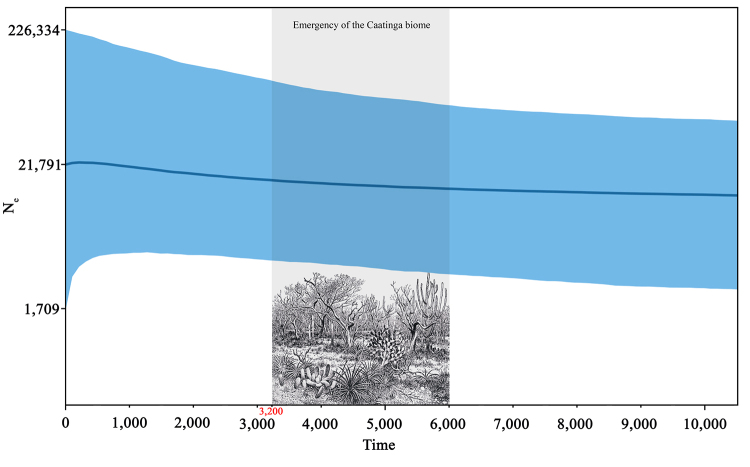
Bayesian Skyline Plot (BSP) with*CYTB*data with a mutation rate of 2x10^-8^ mutations *per* site *per* year. The Y-axis indicates the effective population size (N_e_; weighted, considering males and females), while the X axis indicates the time in thousands of years before the present. The solid line represents the median, while the blue bands represent the 95% higher posterior density (HPD) ranges. The figure representing the Caatinga biome is called “Caatingas” from Bico de pena by Percy Lau (1940) taken from Bernardes (1999).

Our Bayesian Skyline Plot (BSP) showed a tendency of population expansion during the Holocene in the last ~10,000 years, with a current median N_e_ of 21,791 individuals and a wide 95% High Posterior Density (HPD) interval (1,709 - 226,334) (Figure 2). No previous Holocene demographic signals were detected. LAMARC software estimated a current N_e_ of 14,550 individuals, with a tighter 95% HPD confidence interval (5,783 - 30,350). The growth factor in LAMARC showed population expansion, however, it was not statistically significant (Table S15). Because of the wide BSP interval and this result in LAMARC, we decided to compare different demographic scenarios using Approximate Bayesian Computation (ABC).

More specifically, we used ABC to compare population stability, bottleneck, expansion, and transitory bottleneck scenarios based on estimates obtained from BSP and LAMARC (Table S15), reaching a total of eight scenarios. The population expansion scenarios in the Holocene was favored for both BSP and LAMARC estimates. We also performed an ABC analysis with only these two expansion scenarios. Between them, the LAMARC estimate was selected in the ABC analysis by 85% in the logistic regression analysis (Figure S5), with an estimated current median N_e_ of 21,800 individuals (95% HPD, 11,800 - 29,400) and an ancestral N_e_ of 12,900 individuals (95% HPD, 6,320 - 24,100) (Figure S5 and Figure S6, Table S15). In other words, the Caatinga *S. libidinosus* experienced a population expansion of approximately 70% in the last ~3.3 kya. These results suggest that the population size of these capuchins was not significantly impacted by the emergence of the drier and more hostile Caatinga environment during the middle Holocene. 

### Species distribution modeling (SDM)

**Figure 3 -:**
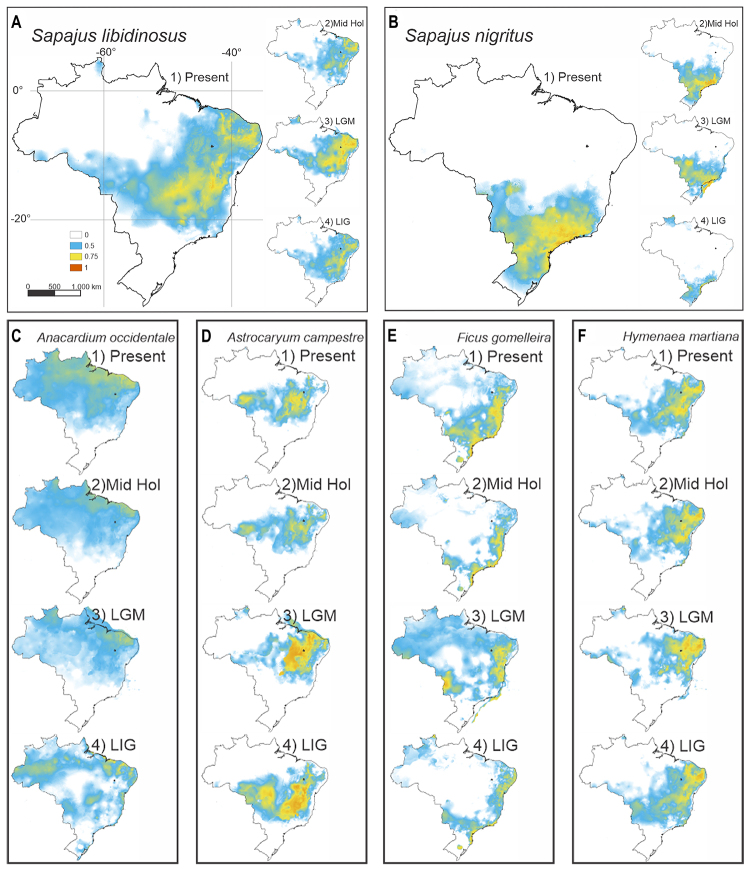
Predicted present and past habitat suitability for (A)*Sapajus libidinosus*, (B)*Sapajus nigritus*, (C)*Anacardium occidentale*, (D)*Astrocarium campestre*, (E)*Ficus gomelleira,*and (F)*Hymenaea martiana.*Predicted distributions were inferred as a function of bioclimatic variables from the (1) Present, (2) Middle Holocene (~6 kya), (3) Last Glacial Maximum (LGM, ~22 kya), and (4) Last Interglacial (LIG, ~140-120 kya) periods. White indicates areas of unsuitable habitat, with green, yellow, and red indicating increasing habitat suitability for the corresponding species. The Serra da Capivara National Park, located in Piauí (Figure S1), is marked on the maps.

Our study used species distribution modeling (SDM) to investigate the availability of plants that *Sapajus libidinosus* uses as food after being processed or accessed through tools. Our SDMs showed that the plant species *Anacardium occidentale*, *Astrocaryum campestre*, and *Hymenaea martiana* had suitable habitats in both present and past periods (Figure 3). These findings also suggest that these plants, but not others, (Figure S7) were available during the establishment of the Caatinga biome in the middle-late Holocene, as previously reported (6-3.2 kya,  De Oliveira et al., 1999; Behling et al., 2000; Pessenda et al., 2010; Novello et al., 2012; Mendes, 2016; De Medeiros et al., 2018). Our analysis also revealed that this period showed the only significant sign of slight population growth of the Caatinga *S. libidinosus* detected using BSP, LAMARC, and ABC (Figure 2, Table S15). Interestingly, we observed that the distribution model of *Ficus gomelleira* predicted significant habitat loss in the Caatinga area during the Holocene, suggesting that access to soft fruits may have been challenging during that period (Figure 3). 

## Discussion

### Demography and evolution of S. libidinosus from the Caatinga. 

The low *CYTB* diversity found in the Pedra Furada (PF) site suggests that the present-day group of *S. libidinosus* living there was established by a small number of females or possibly closely related female founders. Subsequently, vegetative population growth promoted the existence of a healthy natural population and holder of remarkable culture in that site. Despite the potential consequences of inbreeding depression, it is well-established that a rapid population expansion can occur after a severe bottleneck (Kirkpatrick and Jarne, 2000). However, our study cannot ascertain whether the current PF group represents the descendants of an ancient population that continuously inhabited the area and occasionally experienced genetic bottlenecks or if it originated from a few related *S. libidinosus* individuals from other SCNP regions that re-colonized the area. 

Falótico et al. (2019) showed that fluctuations in the availability of cashew trees (*Anacardium occidentale*) in the Pedra Furada site could create waves of capuchin monkey occupation and moments of cultural diversification. Our species distribution models (SDMs) indicated a continuous presence of cashew trees in the region since the occupation of the current Caatinga territory by the *Sapajus* species, albeit with fluctuations. However, our analysis did not evaluate punctual and seasonal fluctuations within smaller time frames. These waves of *S. libidinosus* occupation may also be associated with prehistoric human movements in the Pedra Furada area, which is well-documented (Strauss et al., 2018).

Due to the low *CYTB* diversity, we could not accurately reconstruct the demographic events of the PF population of *Sapajus libidinosus* using methodologies such as BSP, LAMARC, and ABC. In contrast, the *CYTB* diversity in the Caatinga is higher, making it possible to recover more general demographic events, particularly those from the Holocene (12-10 kya to present), when the current semiarid conditions of the Caatinga were established (6-3.2 kya). This period is much more recent than the estimated invasion date of *Sapajus* species into the NE Brazilian region. Therefore, during the last ~200 kya, these environments underwent many climate changes, including glacial cycles (De Oliveira et al., 2020), affecting the flora and fauna distribution in the region (Mares et al., 1985; Cruz et al., 2009). Our habitat suitability reconstruction analysis of capuchin monkeys and selected plants of interest indicated that these past climatic events had a significant impact in the distribution of these species in the region. 

However, our demographic analyses rescued a stable N_e_, with subtle but constant growth, for the *S. libidinosus* population in the Caatinga only throughout the Holocene period. The absence of any previous demographic signals before the Holocene could be due to two reasons. Firstly, any earlier demographic events may have been eroded or hidden by the demographic events that occurred during the Holocene. While there is no evidence of significant population movement in the Holocene that might have masked earlier events, it is also possible that the current sample fails to capture older, more dramatic demographic events, as only a single locus (*CYTB*) is being considered. Future studies should consider nuclear multiloci analysis to gain a more accurate understanding of other potential demographic events. 

### Population expansion of S. libidinosus from the Caatinga and their culture 

We suggest that the Holocene steady demographic expansion of the Caatinga *S. libidinosus*, even with the establishment of more severe climatic conditions*,* may be related to a relevant event in the successful evolutionary history of this species in that biome: the use of tools and the development of autochthonous culture, as seen in the Pedra Furada group. Although some morphological modifications of *S. libidinosus* (limb proportions and tail length) are potentially associated with adaptations to the semiarid environment (Lynch Alfaro et al., 2015; Lima et al., 2017), others are not, especially those expected due to the type of diet, such as a fragile skeletal structure (Moura and Lee, 2004; Moura, 2007; Falótico et al., 2022). Our results indicated that critical environmental challenges in the Holocene triggered natural selection pressure in *S. libidinosus* individuals, leading to innovations in obtaining food in the Caatinga through the use of tools. Moura (2007) reported that *S. libidinosus* in the Caatinga had an average group size within the range reported for Amazonian and Atlantic forests, which differs from that found for other Platyrrhini species investigated by them.

It is noteworthy that the cognitive capacity of *S. libidinosus* (and probably the genetic repertoire responsible for it) was already in place, as the *Cebus/Sapajus* clade is the only Platyrrhini branch with a high and positive general intelligence index that arose independently in four events within the order Primates (Reader et al., 2011). Similarly, the skills to innovate and use tools are already known in the *Cebus/Sapajus* species. However, we suggest that the *S. libidinosus* population that inhabited the northeast region of Brazil began to experience selective pressure, especially when climate change brought a savanna-like ecosystem to the NE Brazilian region, particularly the Caatinga. In response, they developed innovations in tool use and associated behaviors. They are also tolerant and adept at social learning over time, essential characteristics for a transgenerational culture to emerge.

Our SDM analyses also showed that some plant species with encapsulated fruits, currently processed by*S. libidinosus*, were present during the Holocene in refuges in the SCNP landscape. On the other hand, there was potential habitat loss for the*Ficus gomelleira*in the Caatinga area in the middle Holocene, signaling that more easily consumed fruits could be scarce in specific periods.

Among the several theories on the emergence of tool use in primates, the so-called “necessity hypothesis” states that the primary function of the use of tools by primates is to obtain alternative or fallback foods, especially in environments where the conditions do not favor the acquisition of the preferred food in a comfortable and/or abundant way during the year. This type of scenario would be particularly present in savanna-like environments, promoting selective force necessary for innovation and an adaptive culture to emerge, at least among primates (Harrison and Marshall, 2011). The necessity hypothesis is a more recent version of the ideas postulated by Parker and Gibson (1977), in which the use of tools is more likely to arise in omnivorous primate species that forage and feed on encapsulated fruits and other non-accessible foods without the use of tools, in specific periods, when easily accessible resources are scarce. Moura and Lee (2004) investigated*S. libidinosus,*an omnivorous species*,* in* *the SCNP and suggested that when tools are easy to obtain, they potentially reduce the time and cost of processing encapsulated fruits. According to these authors, capuchin monkeys use tools in wild areas where food bottlenecks are frequent due to climate change, such as in the Caatinga biome (Moura and Lee, 2004). This suggestion is consistent with the hypothesis of necessity. An alternative hypothesis suggested by Falótico et al. (2017), tested in another population of*S. libidinosus*(not just those that inhabit the SCNP), is the “opportunity hypothesis”. The opportunity hypothesis involves a high degree of terrestriality, which has been reported among*S. libidinosus*individuals (27% to 43%; Falótico and Ottoni 2023; Wright et al., 2019), allowing them to have more chances to interact with the raw material needed for stone tool use (stones, nuts, and roots). 

In our view, and based on the present study, the hypotheses of necessity and opportunity are not mutually exclusive. The Caatinga is characterized by a semiarid and unpredictable climate (Mares et al., 1985; Moura and Lee, 2004; Abreu et al., 2016) and a hostile environment for animals (Ryland et al., 1997; Alves et al., 2016) and plants (Moura, 2007; Silva and Souza, 2018). As a result, the abundance of plant and animal specimens and species diversity decreased gradually during the establishment of the Caatinga, representing only a fraction of what they were in the past (De Medeiros et al., 2018). Our demographic analyses show that during Holocene the Caatinga*S. libidinosus*N_e_ remained stable, with a continuous growth from that to the present day, particularly notable in the last 3.3 kya. It is known that a minimum population size is also required for a culture to persist over time because it minimizes losses due to drift and facilitates innovation (Rorabaugh, 2014). This stability in population size must also favor the Caatinga*S. libidinosus*culture as a whole. Signatures of tool use by*S. libidinosus*in* *SCNP refer to at least ~3 kya (Falótico et al., 2019).

The regular use of tools, even in seasonal/annual times when easily accessible foods are abundant, that is, without the need for processing them with tools, as observed by Falótico et al. (2017), favors the opportunity hypothesis. However, it does not exclude the possibility that fitness problems during*S. libidinosus*evolution in the Caatinga have been an essential trigger for the natural selection pressure (necessity hypothesis), culminating in the*S. libidinosus*habitual use of tools in that savanna-like biome.

The existence of an adaptive, autochthonous, stable, and dynamic *S. libidinosus* culture over thousands of years in the Caatinga evokes several attributes and conditions previously described for chimpanzees (Whiten et al., 2009). For instance, there are evident and well-known cognitive abilities (coupled with a large brain; Reader et al., 2011). Other relevant causal factors, as already mentioned, include the availability of resources, the readiness to develop innovations, and the ability to transmit them through social learning. Undoubtedly, the manifestation of adaptive behaviors in animals across successive generations is closely linked to a genetic repertoire responsible for expressing such traits that have evolved under the selective pressures requiring cognitively demanding solutions. In addition, sensorimotor intelligence is also required (Schrago, 2022). However, despite extensive research on capuchin monkey behavior, the complete genetic repertoire underlying the development of advanced cognition/intelligence in *Sapajus* remains to be discovered (Schrago, 2022). All these elements together promote a “storm” of gene-culture coevolution (GCC). In other words, the *Sapajus* cognition/intelligence genetic repertoire carries a fitness advantage, enhanced by cultural attributes (*e.g*., tool use), enabling its spread in tandem, similar to other described GCC processes (Waring and Wood, 2021).

Furthermore, like chimpanzees, there is the persistence of constructed niches, where the artifacts, including full hammer-anvil sets, are preserved and re-used through the*S. libidinosus*generations, which favors social learning (Fragaszy et al., 2013; Ottoni, 2021). It is noteworthy that social learning is a critical element in developing adaptive and transmissible cultures because it focuses on functional behavioral variants and eliminates accidental occurrences. This dynamic prevents unimportant behaviors from incorporating into the cultural repertoire of the group (Reader et al., 2016). Currently, behaviors associated with using tools by Caatinga*S. libidinosus*adults and learning by the young are firmly rooted in the fact that seasonal fluctuations in the food supply cannot dissipate them. Still, this Caatinga *S. libidinosus*culture is vigorous, dynamic, and diverse, not only by the current use and re-use of tools but also by the relatively constant innovation, particularly in the SCNP and sites such as Boqueirão da Pedra Furada, where are found the most proficient and skillful of all *S. libidinosus* that have been investigated to date (Moura, 2007; Falótico and Ottoni, 2013; Falótico *et al.*, 2019). Furthermore, comparative studies with chimpanzees living in savannas suggested that drier environments are drivers of primate tool use culture and associated behavioral diversity (Kalan et al., 2020).

The potential “hot spot” of innovation and cultural diversification observed in the SCNP/Pedra Furada may be connected to other factors beyond the weather, some of which have already been identified. For example, in SCNP sites, quartzite is the best raw material for stone tools, which is more available than the other already investigated Caatinga/Cerrado sites, where capuchin monkeys use tools. This condition also allows more interaction and innovation (Ottoni, 2021; Falótico et al., 2022). Our genetic data corroborate the nature of an innovative and cultural “hot spot” in the SCNP/ Pedra Furada site since it is clear that the current sampled*S. libidinosus*subpopulation underwent a bottleneck relatively recently (only two mtDNA lineages detected), indicating that the tools stone repertoire observed today may have been improved and diversified relatively in a short time from a few founding individuals. 

In conclusion, based on our findings and of previous authors, we propose that in order for the *S*. *libidinosus* SCNP/Pedra Furada culture to emerge and perpetuate, a combination of factors must occur, including the following: 1) cognitive capacity of some individual in the social group with particular creative and physical gifts to generate and execute the innovation; 2) cognitive capacity in the social group, which allows the social learning and cultural intergenerational inheritance; 3) tolerance between adult members of the social group, including the dominant males, as a factor that also favors social learning and cultural intergenerational inheritance; 4) a complex (and unknown) genetic repertoire behind these adaptive traits; 5) The presence and abundance of natural resources, such as reusable stones for hammers and anvils, along with hidden food resources requiring tool processing (*e.g*., hard fruits, seeds, and roots), turn certain geographical areas such as Pedra Furada site into “hot spots” for innovation and cultural diversification; 6) high degree of terrestriality, increasing the interaction with those natural resources; 7) selective pressure in Holocene as a trigger for the emergence of innovative behavior; 8) selective pressure to maintain the innovation and a continued learning capacity in the predominantly semiarid and unpredictable Caatinga climate, particularly in SCNP microenvironment.

We stress that some of our results should be considered with caution. For instance, the demographic analyses that were performed using only a single maternal marker with a moderate mutation rate, and our sample size were relatively small. Consequently, our findings provided insights primarily into a demographic scenario in the Holocene period, influenced by female-mediated factors. However, these limitations do not invalidate the relevance of our main findings but rather emphasize the need for further investigation.

## Supplementary material

The following online material is available for this article:

Table S1 - Primate species analyzed for the *CYTB* gene and their respective references.

Table S2 - Occurrence data of *Sapajus libidinosus* used for the Species Distribution Modeling

Table S3 - Occurrence data of *Sapajus nigritus* used for the Species Distribution Modeling.

Table S4 - Occurence data of *Astrocarium campestre* used for the Species Distribution Modeling.

Table S5 - Occurence data of *Attalea maripa* used for the Species Distribution Modeling.

Table S6 - Occurence data of *Anacardium occidentale* used for the Species Distribution Modeling.

Table S7 - Occurence data of *Attalea speciosa* used for the Species Distribution Modeling.

Table S8 - Occurence data of *Ficus gomeleria* used for the Species Distribution Modeling.

Table S9 - Occurence data of *Hymenaea martiana* used for the Species Distribution Modeling.

Table S10 - Occurence data of *Manihot dichotoma* used for the Species Distribution Modeling.

Table S11 - Occurence data of *Syagrus coronata* used for the Species Distribution Modeling.

Table S12 - Results of the Species Distribution Models for the two studied primates (*S. libidinosus* and *S. nigritus*) and the nine plant species

Table S13 - Clades in the Primates phylogeny

Table S14 - The number of individuals *pe*r species or group, nucleotide, and haplotype diversities.

Table S15 - Demographic parameters and effective size for each software.

Figure S1 - Brazilian biomes. The intern divisions represent the federation states.

Figure S2 - Historical scenarios analyzed on DIYABC 2.1.

Figure S3 - (A) Phylogenetic tree generated in BEAST, based on *CYTB* sequences.

Figure S4 - Haplotype network based on *CYTB* sequences.

Figure S5 - Comparison of the two best scenarios of each methodology, Bayesian Skyline Plot (BSP) and LAMARC, both selecting population expansion.

Figure S6 - Model verification applying a PCA in the best-supported scenario (Scenario 1) in the DIYABC analysis.

Figure S7 - Species Distribution Model (SDM) results for *Sapajus libidinosus, Attalea speciosa, Attalea maripa, Hymenaea stignocarpa, Syagrus coronate,* and *Manihot dichotoma.*

Supplementary material (Materials and Methods) - Samples, DNA extraction, sequencing, and ethical authorization
