# Supplementary material for: Insights into the evolutionary history of the most skilled tool-handling platyrrhini monkey: Sapajus libidinosus from the Serra da Capivara National Park
Source: Genet Mol Biol. 2023 Nov 10;46(3 Suppl 1):e20230165. doi: 10.1590/1678-4685-GMB-2023-0165 (PMC10637428; doi:10.1590/1678-4685-GMB-2023-0165)
Supplement: Table S1 - [file 1415-4757-GMB-46-3-s1-e20230165-s1.pdf]

Genetics and Molecular Biology

https://doi.org/10.1590/1678-4685-GMB-2023-0165

Supplementary Material to "Insights into the evolutionary history of the most skilled tool-handling platyrrhini monkey: S*apajus libidinosus* from the Serra da Capivara National Park"

Table S1 - Primate species analyzed for the *CYTB* gene and their respective references.

**Species**	**Access number**
Alouatta belzebul	DQ387034.1
Alouatta caraya	KT626649.1
Alouatta guariba	KR528422.1
Alouatta sara	KR902386.1
Alouatta seniculus	HQ644333.1
Alouatta stramineus	AF289983.1
Aotus azarai	KC757385.1
Aotus infulatus	HQ005494.1
Aotus lemurinus	HQ005506.1
Aotus nancymaae	HQ005509.1
Aotus nigriceps	HQ005498.1
Aotus trivirgatus	HQ005499.1
Aotus vociferans	HQ005503.1
Ateles belzebuth	KR902369.1
Ateles geoffroyi	KR902388.1
Ateles paniscus	KM591218.1
Brachyteles arachnoides	AF289989.1/KR528394.1
Cacajao ayresi	EU560411.1
Cacajao hosomi	EU560418.1
Cacajao melanocephalus	FJ531649.1
Callicebus caquetensis	KX353779.1

**Species**	**Access number**
Callicebus coimbrai	Present study
Callicebus cupreus	KC959986.1
Callicebus discolor	KX353788.1
Callicebus donacophilus	FJ785423.1
Callicebus lugens	DQ337707.1
Callimico goeldii	NC_024628.1
Callithrix geoffroyi	KU253509.1
Callithrix jacchus	AY434079.1
Callithrix kuhlii	KU253510.1
Cebuella pygmaea	NC_021942.1
Cebus albifrons	KU694249.1
Cebus capucinus	JN409305.1
Cebus kaapori	KY173241.1
Cebus olivaceus	FJ529106.1
Chiropotes albinasus	NC_021946.1
Chiropotes chiropotes	FJ531667.1
Chiropotes israelita	KC592392.1
Chiropotes utahickae	Present study
Gorilla gorilla	NC_011120.1
Homo neanderthalensis	ENST00000361789
Homo sapiens	J01415.2
Lagothrix lagotricha	KC757398.1
Leontopithecus chrysomelas	KR528398.1
Leontopithecus chrysopygus	MG933868.1
Leontopithecus rosalia	KR528404.1
Mico chrysoleucus	KR528411.1
Mico humaralifer	Present study
Mico melanura	Present study
Mico saterei	Present study
Pan paniscus	GU189672.1

Species	Access number
Pan troglodytes	JF727205.1
Pithecia irrorata	AY226183.1
Pithecia monachus	FJ531668.1
Plecturocebus miltoni	KU694288.1
Pongo abelii	U38274.1
Saguinus_bicolor	KR528403.1
Saguinus cruzlimai	KP182174.1
Saguinus inustus	KM370853.1
Saguinus labiatus	HM367996.1
Saguinus martinsi	Present study
Saguinus midas	AJ489760.1
Saguinus mystax mystax	HM368011.1
Saguinus niger	Present study
Saguinus tripartitus	HM368076.1
Saimiri boliviensis	KR902432.1
Saimiri oerstedii	HQ644336.1
Saimiri sciureus	FJ785425.1
Sapajus apella	KY173251.1
Sapajus cay	FJ529060.1
Sapajus flavius	KR528427.1
Sapajus libidinosus	Present study / Lima et al. (2017)
Sapajus macrocephalus	FJ529102.1
Sapajus nigritus	KY173338.1
Sapajus robustus	KR528406.1
Sapajus xanthosternos	Present study

Note: *Sapajus libidinosus* (*N= 71*) and *Sapajus xanthosternos* (N=6) individuals were sequenced. One individual of each of the other species indicated were also sequenced. They are deposited into Genbank (accession numbers OQ859634 – OQ859709).
