# Supplementary material for: Insights into the evolutionary history of the most skilled tool-handling platyrrhini monkey: Sapajus libidinosus from the Serra da Capivara National Park
Source: Genet Mol Biol. 2023 Nov 10;46(3 Suppl 1):e20230165. doi: 10.1590/1678-4685-GMB-2023-0165 (PMC10637428; doi:10.1590/1678-4685-GMB-2023-0165)
Supplement: Table S2 - [file 1415-4757-GMB-46-3-s1-e20230165-s2.pdf]

Genetics and Molecular Biology

https://doi.org/10.1590/1678-4685-GMB-2023-0165

Supplementary Material to "Insights into the evolutionary history of the most skilled tool-handling platyrrhine monkey: S*apajus libidinosus* from the Serra da Capivara National Park"

Table S2 - Occurrence data of *Sapajus libidinosus* used for the Species Distribution Modeling.

Species	Longitude	Latitude
S. libidinosus	-49,19504347	-14,02914208
S. libidinosus	-48,54999473	-12,03216458
S. libidinosus	-48,40742933	-14,36529863
S. libidinosus	-48,2457107	-13,5859973
S. libidinosus	-47,92719357	-15,73899024
S. libidinosus	-47,43997	-14,10889
S. libidinosus	-46,4551484	-13,40577108
S. libidinosus	-43,498905	-9,219753
S. libidinosus	-42,68666667	-2,621666667
S. libidinosus	-42,60193649	-8,857755447
S. libidinosus	-40,90314384	-3,839450027
S. libidinosus	-38,37488188	-6,391727349
S. libidinosus	-38,29515278	-7,957127778
S. libidinosus	-37,90020052	-6,066294181
S. libidinosus	-37,47985095	-6,88785098
S. libidinosus	-37,406387	-9,656667
S. libidinosus	-39,32	-7,93
S. libidinosus	-39,358483	-14,6638167
S. libidinosus	-39,3742231	-14,6678411
S. libidinosus	-39,391518	-14,6732269
S. libidinosus	-39,78	-7,68
S. libidinosus	-41,574763	-10,705236
S. libidinosus	-42,55141563	-8,832027569
S. libidinosus	-42,61355	-9,963192
S. libidinosus	-43,463333	-9,226667
S. libidinosus	-44,4271912	-14,2562958
S. libidinosus	-44,55	-14,17
S. libidinosus	-46,87	-17,22
S. libidinosus	-47,464919	-7,3360699
S. libidinosus	-47,47	-7,33
S. libidinosus	-47,8581116	-19,6474392
S. libidinosus	-47,92	-19,75

Species	Longitude	Latitude
S. libidinosus	-48,0736867	-21,59014
S. libidinosus	-48,18	-18,63
S. libidinosus	-48,18	-8,98
S. libidinosus	-48,2	-15,68
S. libidinosus	-48,2430273	-18,6378424
S. libidinosus	-48,43	-18,48
S. libidinosus	-48,4347	-18,4739893
S. libidinosus	-48,5466477	-17,8458549
S. libidinosus	-48,5788782	-18,5416454
S. libidinosus	-48,6227621	-17,7972102
S. libidinosus	-48,6267996	-17,7785043
S. libidinosus	-48,63	-17,75
S. libidinosus	-48,7	-18,58
S. libidinosus	-49,02	-17,3
S. libidinosus	-50,33	-16,08
S. libidinosus	-57,1899675	-15,1524725
