# Supplementary material for: Insights into the evolutionary history of the most skilled tool-handling platyrrhini monkey: Sapajus libidinosus from the Serra da Capivara National Park
Source: Genet Mol Biol. 2023 Nov 10;46(3 Suppl 1):e20230165. doi: 10.1590/1678-4685-GMB-2023-0165 (PMC10637428; doi:10.1590/1678-4685-GMB-2023-0165)
Supplement: Table S3 - [file 1415-4757-GMB-46-3-s1-e20230165-s3.pdf]

Genetics and Molecular Biology

https://doi.org/10.1590/1678-4685-GMB-2023-0165

Supplementary Material to "Insights into the evolutionary history of the most skilled tool-handling platyrrhini monkey: S*apajus libidinosus* from the Serra da Capivara National Park"

Table S3 - Occurrence data of *Sapajus nigritus* used for the Species Distribution Modeling.

Species	Longitude	Latitude
S. nigritus	-40,60029984	-19,93560028
S. nigritus	-54,319289	-25,68029
S. nigritus	-49,333332	-15,75
S. nigritus	-48,833333	-26,3
S. nigritus	-48,412966	-21,072948
S. nigritus	-40,369722	-19,831944
S. nigritus	-54,44638	-25,687287
S. nigritus	-43,23333	-22,9
S. nigritus	-40,6	-19,92
S. nigritus	-41,95	-6,55
S. nigritus	-42,68	-21,87
S. nigritus	-43,12	-22,15
S. nigritus	-43,33	-21,88
S. nigritus	-43,42	-21,95
S. nigritus	-44,3	-23
S. nigritus	-44,3	-18,43
S. nigritus	-45,07	-23,43
S. nigritus	-45,25	-5,5
S. nigritus	-45,95	-21,43
S. nigritus	-46,38	-20,87
S. nigritus	-47,02	-21,47
S. nigritus	-49,67	-26,92
S. nigritus	-49,75	-21,666666
S. nigritus	-50,23	-20,27
S. nigritus	-51,02	-22,8
S. nigritus	-51,48	-23,55
S. nigritus	-51,65	-23,6
S. nigritus	-51,83	-21,87
S. nigritus	-52,32	-27,27
S. nigritus	-52,6	-27,1
S. nigritus	-53,78	-27,85
S. nigritus	-54,44	-25,7
S. nigritus	-46,082055	-21,535187
S. nigritus	-45,988744	-21,578251
S. nigritus	-51,06644	-23,054796
S. nigritus	-51,243353	-23,443279
