# Supplementary material for: Insights into the evolutionary history of the most skilled tool-handling platyrrhini monkey: Sapajus libidinosus from the Serra da Capivara National Park
Source: Genet Mol Biol. 2023 Nov 10;46(3 Suppl 1):e20230165. doi: 10.1590/1678-4685-GMB-2023-0165 (PMC10637428; doi:10.1590/1678-4685-GMB-2023-0165)
Supplement: Table S4 - [file 1415-4757-GMB-46-3-s1-e20230165-s4.pdf]

Genetics and Molecular Biology

https://doi.org/10.1590/1678-4685-GMB-2023-0165

Supplementary Material to Insights into the evolutionary history of the most skilled tool-handling platyrrhini monkey: S*apajus libidinosus* from the Serra da Capivara National Park"

Table S4 - Occurence data of *Astrocarium campestre* used for the Species Distribution Modeling.

Species	Longitude	Latitude
A. campestre	-45,15075	-17,277194
A. campestre	-46,32925	-13,371417
A. campestre	-65,031111	-9,6
A. campestre	-44,701944	-12,416667
A. campestre	-45,368611	-13,701944
A. campestre	-45,451944	-14,309167
A. campestre	-45,285278	-12,166667
A. campestre	-44,951944	-12,559167
A. campestre	-46,451944	-13,475833
A. campestre	-41,790556	-13,588056
A. campestre	-41,4	-12,95
A. campestre	-46,585278	-14,200278
A. campestre	-42,503333	-14,131667
A. campestre	-44,766667	-12,6
A. campestre	-44,966667	-12,35
A. campestre	-42,505278	-14,088611
A. campestre	-42,493056	-14,184444
A. campestre	-44,666667	-14,381389
A. campestre	-41,894167	-13,08
A. campestre	-41,366667	-13,1
A. campestre	-44,533333	-12,3
A. campestre	-44,65	-13,333333
A. campestre	-44,55	-14,166667
A. campestre	-45,986944	-10,714444
A. campestre	-46,5	-12,75
A. campestre	-47,416666	-6,3333333
A. campestre	-46,5	-5,75
A. campestre	-46,607777	-10,374722
A. campestre	-47,550833	-7,3636111
A. campestre	-47,91	-7,895833
A. campestre	-47,370277	-7,1258333
A. campestre	-45,387222	-15,4825
A. campestre	-46,361667	-10,570556
A. campestre	-46,766667	-15,133333

Species	Longitude	Latitude
A. campestre	-45,6675	-15,097222
A. campestre	-47,45	-7,033333
A. campestre	-47,5	-8,5
A. campestre	-54,916667	-18,95
A. campestre	-54,732778	-17,820278
A. campestre	-45,8575	-6,615
A. campestre	-43,366417	-10,680167
A. campestre	-57,833333	-8
A. campestre	-48,062222	-13,436667
A. campestre	-50,82109833	-1,935559988
A. campestre	-46,083333	-7,583333
A. campestre	-48	-10,295833
A. campestre	-41,795556	-13,597222
A. campestre	-46,333333	-14,25
A. campestre	-44,333333	-7,416667
A. campestre	-51,8275	-16,933333
A. campestre	-51,440556	-17,840833
A. campestre	-50,226944	-18,913056
A. campestre	-46,216667	-14,016667
A. campestre	-46,066667	-17,916667
A. campestre	-46,740556	-10,939722
A. campestre	-41,916667	-13,533333
A. campestre	-41,933056	-13,08
A. campestre	-45,0325	-13,659167
A. campestre	-42,3075	-13,954167
A. campestre	-42,5	-14,066667
A. campestre	-44,910833	-13,780278
A. campestre	-46,597222	-10,597861
A. campestre	-46,593167	-10,603528
A. campestre	-53,884611	-16,867972
A. campestre	-47,5	-6,5
A. campestre	-47,489806	-6,990139
A. campestre	-46,004444	-14,826667
A. campestre	-44,5	-7,25
A. campestre	-60,72	-13,98
A. campestre	-61,5	-17,5
