# Supplementary material for: Insights into the evolutionary history of the most skilled tool-handling platyrrhini monkey: Sapajus libidinosus from the Serra da Capivara National Park
Source: Genet Mol Biol. 2023 Nov 10;46(3 Suppl 1):e20230165. doi: 10.1590/1678-4685-GMB-2023-0165 (PMC10637428; doi:10.1590/1678-4685-GMB-2023-0165)
Supplement: Table S5 - [file 1415-4757-GMB-46-3-s1-e20230165-s5.pdf]

Genetics and Molecular Biology

https://doi.org/10.1590/1678-4685-GMB-2023-0165

Supplementary Material to “Insights into the evolutionary history of the most skilled tool-handling platyrrhini monkey: S*apajus libidinosus* from the Serra da Capivara National Park”

Table S5 - Occurence data of *Attalea maripa* used for the Species Distribution Modeling.

Species	Longitude	Latitude
A. maripa	-44,23500061	-2,934439898
A. maripa	-45,5	-3,5
A. maripa	-46,08	-3,12
A. maripa	-46,62888889	-0,99597222
A. maripa	-46,67138889	-0,91694444
A. maripa	-46,916666	-1,0833333
A. maripa	-47,34	-5,94
A. maripa	-47,95	-5,02
A. maripa	-48,484525	-0,668956
A. maripa	-48,51139832	-5,342780113
A. maripa	-48,976111	-1,619167
A. maripa	-49,639546	-1,612315
A. maripa	-49,683333	-0,75
A. maripa	-50,177222	-6,13
A. maripa	-52,8731	-10,8339
A. maripa	-54,41666667	4,16666667
A. maripa	-54,7	-2,6
A. maripa	-55	5,42
A. maripa	-55,083333	5,5
A. maripa	-55,179444	-10,453861
A. maripa	-55,43472	3,96805
A. maripa	-55,572222	-11,161667
A. maripa	-56,357222	-10,344556
A. maripa	-56,45381	4,3025
A. maripa	-57,16666667	5,66666667
A. maripa	-58,5	4,37
A. maripa	-59,05	4,17
A. maripa	-59,25	2,17
A. maripa	-59,35	3,02
A. maripa	-59,5	3,37
A. maripa	-59,7	7,366667
A. maripa	-59,966667	-2,883333
A. maripa	-60,78333	-14,6
A. maripa	-61,00657	-1,29118

Species	Longitude	Latitude
A. maripa	-61,07	2,148194
A. maripa	-61,15	-14,7
A. maripa	-61,2	-12,24
A. maripa	-61,27043	-3,22551
A. maripa	-61,47755	-3,39141
A. maripa	-62,033333	-7,966667
A. maripa	-62,6166667	-14,6333333
A. maripa	-62,9	9,9
A. maripa	-63,12	10,5014
A. maripa	-63,4	4,97
A. maripa	-63,5264	6,3194
A. maripa	-64,23	2,97
A. maripa	-61,85	8,55
A. maripa	-55,92	-22,32
A. maripa	-72,7725	2,580278
A. maripa	-67,86	5,33
A. maripa	-62,283333	8,466667
A. maripa	-46,92	-1,083333
A. maripa	-64,46666	6,21666
A. maripa	-75,45	-1
A. maripa	-74,83333	-10,16666
A. maripa	-75,21666	-10,16666
A. maripa	-47,59	-1,3
A. maripa	-66,03333	-11,75
A. maripa	-52,516667	-4,816667
A. maripa	-65,023417	-10,811472
A. maripa	-65,56666667	2,16666667
A. maripa	-66,92	1,27
A. maripa	-67	1,916667
A. maripa	-67,6	5,58
A. maripa	-67,77	5,02
A. maripa	-68,024444	5,355556
A. maripa	-68,3161	-10,5711
A. maripa	-68,323	3,987
A. maripa	-68,667778	5,84075
A. maripa	-68,67	-12,53
A. maripa	-68,849167	-13,03
A. maripa	-69,25	-11,33
A. maripa	-69,92	-4,09
A. maripa	-70,082	-12,542
A. maripa	-71,14389	2,60167
A. maripa	-71,4	2,78
A. maripa	-71,5825	4,868611
A. maripa	-72,248611	-0,606389
A. maripa	-72,35	-0,61
A. maripa	-72,423611	0,213056
A. maripa	-72,91666	-3,33333
A. maripa	-72,92	-7,67

Species	Longitude	Latitude
A. maripa	-73,1639	-5,2917
A. maripa	-73,319	-3,688
A. maripa	-73,33	-4,12
A. maripa	-73,33	-4,17
A. maripa	-73,333	-4,116
A. maripa	-73,333	-3,933
A. maripa	-73,43	-4,00416
A. maripa	-73,443	-4
A. maripa	-73,466	-3,783
A. maripa	-73,583	-4,283
A. maripa	-73,721667	3,695278
A. maripa	-73,75	-3,075
A. maripa	-73,75	-4,92
A. maripa	-74	2,083333
A. maripa	-75	-4
A. maripa	-75,728333	1,262778
A. maripa	-76,1666667	0,0166667
A. maripa	-76,18333	0,01666
A. maripa	-76,22	-5,97
A. maripa	-76,24285	-0,8726167
A. maripa	-76,38	-0,53
A. maripa	-76,38333	-0,66666
A. maripa	-76,4645833	-0,6425833
A. maripa	-76,8	-2,5166667
A. maripa	-77,3	-2,13333
A. maripa	-77,6	-2,78
A. maripa	-73,368284	-3,805585
A. maripa	-55,345312	-3,391552
A. maripa	-74,166667	2,666667
A. maripa	-57,493696	5,800849
A. maripa	-52,407497	-1,065947
A. maripa	-59,9250271	-2,9633385
A. maripa	-77,67	-1,83
A. maripa	-55,450354	3,332707
A. maripa	-73,482116	-3,940439
