# Supplementary material for: Insights into the evolutionary history of the most skilled tool-handling platyrrhini monkey: Sapajus libidinosus from the Serra da Capivara National Park
Source: Genet Mol Biol. 2023 Nov 10;46(3 Suppl 1):e20230165. doi: 10.1590/1678-4685-GMB-2023-0165 (PMC10637428; doi:10.1590/1678-4685-GMB-2023-0165)
Supplement: Table S6 - [file 1415-4757-GMB-46-3-s1-e20230165-s6.pdf]

Genetics and Molecular Biology

https://doi.org/10.1590/1678-4685-GMB-2023-0165

Supplementary Material to "Insights into the evolutionary history of the most skilled tool-handling platyrrhini monkey: S*apajus libidinosus* from the Serra da Capivara National Park"

Table S6 - Occurence data of *Anacardium occidentale* used for the Species Distribution Modeling.

Species	Longitude	Latitude
A. occidentale	-100,003056	16,985278
A. occidentale	-100,084167	16,993056
A. occidentale	-102	23
A. occidentale	-34,801917	-7,365583
A. occidentale	-34,824389	-7,326861
A. occidentale	-34,849528	-7,390778
A. occidentale	-34,853417	-7,275444
A. occidentale	-34,922583	-6,796056
A. occidentale	-34,9575	-7,26
A. occidentale	-34,9769	-6,5022
A. occidentale	-35,013611	-6,392778
A. occidentale	-35,018611	-7,775278
A. occidentale	-35,02325	-6,373833
A. occidentale	-35,02495	-6,394783
A. occidentale	-35,095	-8,714444
A. occidentale	-35,126111	-5,990833
A. occidentale	-35,129167	-6,604444
A. occidentale	-35,14055556	-5,99611111
A. occidentale	-35,141944	-6,741944
A. occidentale	-35,166111	-6,697778
A. occidentale	-35,194717	-5,931033
A. occidentale	-35,23861111	-6,61472222
A. occidentale	-35,28722222	-5,47444444
A. occidentale	-35,375	-5,883333
A. occidentale	-35,585278	-6,892222
A. occidentale	-35,639992	-6,659994
A. occidentale	-35,661139	-7,193083
A. occidentale	-35,685005	-7,019993
A. occidentale	-35,795458	-9,55775
A. occidentale	-35,7969017	-8,233329773
A. occidentale	-35,85	-9,3
A. occidentale	-35,860858	-9,769575
A. occidentale	-35,87	-9,7
A. occidentale	-35,893411	-9,697808

Species	Longitude	Latitude
A. occidentale	-35,922222	-8,238333
A. occidentale	-35,933417	-8,248139
A. occidentale	-35,959993	-6,84001
A. occidentale	-35,96060181	-7,161109924
A. occidentale	-36,18833333	-6,06222222
A. occidentale	-36,30305556	-10,29388889
A. occidentale	-36,3454	-10,2744
A. occidentale	-36,374722	-10,252778
A. occidentale	-36,38181	-10,15224
A. occidentale	-36,389444	-9,013611
A. occidentale	-36,427672	-9,228003
A. occidentale	-36,46	-9,938889
A. occidentale	-36,51222222	-10,44166667
A. occidentale	-36,5257	-10,3758
A. occidentale	-36,782222	-10,641944
A. occidentale	-36,804444	-10,640833
A. occidentale	-36,829444	-10,699444
A. occidentale	-36,949997	-9,733333
A. occidentale	-37,02194444	-10,54333333
A. occidentale	-37,025414	-9,971172
A. occidentale	-37,0315	-10,4721
A. occidentale	-37,04166667	-10,54388889
A. occidentale	-37,054667	-10,536861
A. occidentale	-37,061	-10,5537
A. occidentale	-37,0934	-10,5802
A. occidentale	-37,12222222	-11,00527778
A. occidentale	-37,148333	-10,688611
A. occidentale	-37,17199	-10,18156
A. occidentale	-37,18361111	-10,59527778
A. occidentale	-37,1854	-10,4529
A. occidentale	-37,1944	-11,0429
A. occidentale	-37,2027	-10,4554
A. occidentale	-37,207439	-11,074844
A. occidentale	-37,238361	-8,580805
A. occidentale	-37,241411	-8,525008
A. occidentale	-37,26166667	-11,16083333
A. occidentale	-37,292472	-11,317583
A. occidentale	-37,299167	-7,065278
A. occidentale	-37,355833	-10,773056
A. occidentale	-37,36342	-9,79139
A. occidentale	-37,39	-10,55055556
A. occidentale	-37,398333	-7,198444
A. occidentale	-37,424278	-11,378056
A. occidentale	-37,478519	-11,226019
A. occidentale	-37,511389	-10,519444
A. occidentale	-37,571094	-11,513091
A. occidentale	-37,575833	-11,871667
A. occidentale	-37,597222	-11,889722

Species	Longitude	Latitude
A. occidentale	-37,604389	-8,205
A. occidentale	-37,61	-10,55371
A. occidentale	-37,65	-10,918056
A. occidentale	-37,704	-6,445333
A. occidentale	-37,719167	-8,01
A. occidentale	-37,766389	-11,483333
A. occidentale	-37,773056	-12,024444
A. occidentale	-37,783333	-11,55
A. occidentale	-37,866667	-12,329167
A. occidentale	-37,888111	-6,478444
A. occidentale	-37,89	-12,329167
A. occidentale	-37,910667	-5,688053
A. occidentale	-37,91579819	-6,570559978
A. occidentale	-37,979444	-12,374444
A. occidentale	-38,083175	-8,182994
A. occidentale	-38,166667	-12,343889
A. occidentale	-38,201944	-8
A. occidentale	-38,209167	-7,411944
A. occidentale	-38,25	-9,201944
A. occidentale	-38,279028	-3,974139
A. occidentale	-38,283333	-12,516667
A. occidentale	-38,309167	-11,916667
A. occidentale	-38,333333	-12,016667
A. occidentale	-38,365833	-12,5775
A. occidentale	-38,367778	-6,164167
A. occidentale	-38,375	-6,172222
A. occidentale	-38,383333	-6,266667
A. occidentale	-38,383333	-6,166667
A. occidentale	-38,398333	-10,25
A. occidentale	-38,4	-12,933333
A. occidentale	-38,4	-12,166667
A. occidentale	-38,4225	-12,185556
A. occidentale	-38,433333	-9,966667
A. occidentale	-38,48	-11,956389
A. occidentale	-38,483333	-12,5
A. occidentale	-38,505833	-12,160278
A. occidentale	-38,5426	-7,1752
A. occidentale	-38,61470032	-10,57689953
A. occidentale	-38,6175	-10,617222
A. occidentale	-38,618611	-6,25
A. occidentale	-38,65	-10,866667
A. occidentale	-38,65	-10,42
A. occidentale	-38,66944	-10,669167
A. occidentale	-38,67444	-10,656111
A. occidentale	-38,706625	-4,424508
A. occidentale	-38,725583	-7,432111
A. occidentale	-38,767222	-3,611944
A. occidentale	-38,775556	-8,845278

Species	Longitude	Latitude
A. occidentale	-38,785278	-7,6425
A. occidentale	-38,816667	-3,566667
A. occidentale	-38,817111	-4,246833
A. occidentale	-38,85	-12,533333
A. occidentale	-38,880008	-3,599997
A. occidentale	-38,883333	-4,9
A. occidentale	-38,916667	-12,766667
A. occidentale	-38,940722	-7,297778
A. occidentale	-38,95	-12,55
A. occidentale	-38,961405	-6,713091
A. occidentale	-38,964167	-16,040556
A. occidentale	-38,981389	-14,106944
A. occidentale	-38,994722	-15,334444
A. occidentale	-39	-14,1
A. occidentale	-39,005888	-9,917972
A. occidentale	-39,033333	-13,483333
A. occidentale	-39,039317	-16,289196
A. occidentale	-39,044444	-14,520833
A. occidentale	-39,045556	-15,299167
A. occidentale	-39,059994	-3,419986
A. occidentale	-39,073333	-14,469167
A. occidentale	-39,075278	-4,485833
A. occidentale	-39,083333	-15,15
A. occidentale	-39,083333	-16,373055
A. occidentale	-39,083333	-12,533333
A. occidentale	-39,083333	-12,633333
A. occidentale	-39,116667	-14,683333
A. occidentale	-39,118147	-7,529027
A. occidentale	-39,118611	-7,285278
A. occidentale	-39,174167	-17,668056
A. occidentale	-39,207778	-13,905278
A. occidentale	-39,221	-8,118944
A. occidentale	-39,2221999	-17,4948313
A. occidentale	-39,283639	-7,05125
A. occidentale	-39,292222	-13,901111
A. occidentale	-39,292294	-4,945789
A. occidentale	-39,33	-13,383611
A. occidentale	-39,332639	-7,176417
A. occidentale	-39,333056	-7,398333
A. occidentale	-39,338056	-17,380278
A. occidentale	-39,392222	-17,300556
A. occidentale	-39,408333	-7,326944
A. occidentale	-39,415833	-17,274444
A. occidentale	-39,420006	-3,599997
A. occidentale	-39,454167	-17,949167
A. occidentale	-39,454167	-7,275556
A. occidentale	-39,466667	-14,983333
A. occidentale	-39,470556	-7,337778

Species	Longitude	Latitude
A. occidentale	-39,483333	-12,683333
A. occidentale	-39,5	-13,233333
A. occidentale	-39,500103	-7,408192
A. occidentale	-39,513889	-7,265833
A. occidentale	-39,525	-14,578611
A. occidentale	-39,545833	-16,418333
A. occidentale	-39,568611	-7,994167
A. occidentale	-39,577222	-13,736111
A. occidentale	-39,590833	-17,196667
A. occidentale	-39,596667	-7,208889
A. occidentale	-39,6	-13,333333
A. occidentale	-39,616906	-15,167825
A. occidentale	-39,693889	-18,403889
A. occidentale	-39,7025	-18,405833
A. occidentale	-39,707778	-17,997222
A. occidentale	-39,711389	-18,423056
A. occidentale	-39,722222	-14,167222
A. occidentale	-39,733889	-18,620833
A. occidentale	-39,966667	-16,616667
A. occidentale	-40,007777	-9,185527
A. occidentale	-40,046277	-11,596775
A. occidentale	-40,13083333	-19,86944444
A. occidentale	-40,193444	-13,212806
A. occidentale	-40,256306	-7,195361
A. occidentale	-40,350278	-20,386667
A. occidentale	-40,439222	-7,334194
A. occidentale	-40,4563	-2,8728
A. occidentale	-40,482778	-12,303889
A. occidentale	-40,490556	-12,305278
A. occidentale	-40,499722	-13,320556
A. occidentale	-40,507222	-11,195833
A. occidentale	-40,558361	-9,328944
A. occidentale	-40,576667	-13,002778
A. occidentale	-40,870556	-9,6425
A. occidentale	-40,923283	-5,800467
A. occidentale	-40,92725	-5,147194
A. occidentale	-41,256944	-12,225833
A. occidentale	-41,283333	-13,8
A. occidentale	-41,34388889	-9,97388889
A. occidentale	-41,343911	-9,976119
A. occidentale	-41,365278	-12,544722
A. occidentale	-41,383333	-12,566667
A. occidentale	-41,432778	-16,353889
A. occidentale	-41,45	-3,500556
A. occidentale	-41,472778	-13,583333
A. occidentale	-41,529789	-5,340033
A. occidentale	-41,551944	-22,233333
A. occidentale	-41,55889893	-12,52890015

Species	Longitude	Latitude
A. occidentale	-41,669167	-4,100833
A. occidentale	-41,6833333	-4,1166667
A. occidentale	-41,683806	-4,12875
A. occidentale	-41,693139	-4,1275
A. occidentale	-41,696389	-4,085278
A. occidentale	-41,702583	-4,115
A. occidentale	-41,713889	-4,099722
A. occidentale	-41,717	-2,885917
A. occidentale	-41,717528	-2,970056
A. occidentale	-41,725278	-2,970056
A. occidentale	-41,736167	-3,105139
A. occidentale	-41,742306	-3,359028
A. occidentale	-41,770639	-2,819528
A. occidentale	-41,772917	-3,076861
A. occidentale	-41,774139	-3,365417
A. occidentale	-41,786944	-3,0866666
A. occidentale	-41,795833	-2,957778
A. occidentale	-41,812778	-2,808056
A. occidentale	-41,82139969	-2,858330011
A. occidentale	-41,824556	-2,83725
A. occidentale	-41,841528	-4,119278
A. occidentale	-41,854206	-2,765417
A. occidentale	-41,940003	-3,059999
A. occidentale	-42,072222	-4,865667
A. occidentale	-42,072778	-4,87
A. occidentale	-42,12	-2,7
A. occidentale	-42,183611	-2,731944
A. occidentale	-42,3015	-10,0012
A. occidentale	-42,31666667	-5,35
A. occidentale	-42,366944	-11,759167
A. occidentale	-42,368683	-3,552572
A. occidentale	-42,43972222	-6,43388889
A. occidentale	-42,50439835	-10,0031004
A. occidentale	-42,5103	-22,9203
A. occidentale	-42,556944	-14,323333
A. occidentale	-42,69	-12,401111
A. occidentale	-42,69	-12,034444
A. occidentale	-42,736389	-11,117222
A. occidentale	-42,786944	-6,357222
A. occidentale	-42,8369	-10,075
A. occidentale	-42,837778	-10,083611
A. occidentale	-42,840002	-3,24
A. occidentale	-42,840003	-2,880012
A. occidentale	-42,840011	-3,779992
A. occidentale	-42,861389	-3,621111
A. occidentale	-42,94575	-4,207511
A. occidentale	-42,999306	-5,072306
A. occidentale	-43	-12,866667

Species	Longitude	Latitude
A. occidentale	-43,019722	-5,039722
A. occidentale	-43,020003	-3,599981
A. occidentale	-43,12530136	-21,45750046
A. occidentale	-43,15	-12,866667
A. occidentale	-43,199796	-5,219737
A. occidentale	-43,199984	-3,059982
A. occidentale	-43,2	-5,58
A. occidentale	-43,200001	-2,699996
A. occidentale	-43,233333	-2,55
A. occidentale	-43,333333	-15,75
A. occidentale	-43,379978	-3,420008
A. occidentale	-43,379997	-2,700006
A. occidentale	-43,38	-7,559989
A. occidentale	-43,380003	-4,950004
A. occidentale	-43,405	-10,143333
A. occidentale	-43,448386	-22,913514
A. occidentale	-43,532222	-6,845
A. occidentale	-43,559979	-3,599994
A. occidentale	-43,559989	-4,45497
A. occidentale	-43,559991	-4,5
A. occidentale	-43,559995	-4,320026
A. occidentale	-43,56	-4,679722
A. occidentale	-43,560008	-3,059995
A. occidentale	-43,59277778	-23,03527778
A. occidentale	-43,739722	-6,839722
A. occidentale	-43,740011	-4,5
A. occidentale	-43,740012	-4,679986
A. occidentale	-43,860278	-3,622778
A. occidentale	-43,920005	-3,599996
A. occidentale	-44,028222	-14,741611
A. occidentale	-44,1	-10,98
A. occidentale	-44,1	-5,94
A. occidentale	-44,100004	-3,419999
A. occidentale	-44,1516	-2,646625
A. occidentale	-44,17	-22,855556
A. occidentale	-44,272778	-2,596389
A. occidentale	-44,459989	-11,699956
A. occidentale	-44,459997	-12,566647
A. occidentale	-44,460159	-5,579416
A. occidentale	-44,486944	-13,784167
A. occidentale	-44,5833333	-13,4
A. occidentale	-44,6	-15,533333
A. occidentale	-44,624386	-7,025686
A. occidentale	-44,6275	-13,293056
A. occidentale	-44,63	-15,52
A. occidentale	-44,639986	-7,38
A. occidentale	-44,64	-9,72
A. occidentale	-44,640011	-10,799994

Species	Longitude	Latitude
A. occidentale	-44,640385	-5,939195
A. occidentale	-44,783056	-2,433333
A. occidentale	-44,819722	-11,159722
A. occidentale	-44,819992	-12,24
A. occidentale	-44,820278	-5,4
A. occidentale	-44,85	-12,6
A. occidentale	-44,873472	-1,635767
A. occidentale	-44,874767	-3,171881
A. occidentale	-44,912778	-12,254167
A. occidentale	-45	-10,8
A. occidentale	-45	-7,433333
A. occidentale	-45	-7
A. occidentale	-45,05	-7,38
A. occidentale	-45,10555556	-11,05722222
A. occidentale	-45,18	-8,944406
A. occidentale	-45,180627	-10,260237
A. occidentale	-45,216667	-16,516667
A. occidentale	-45,25138889	-11,01694444
A. occidentale	-45,333333	-7,383333
A. occidentale	-45,359977	-4,680006
A. occidentale	-45,359994	-14,399978
A. occidentale	-45,360003	-6,660002
A. occidentale	-45,366944	-13,524722
A. occidentale	-45,4069	-13,5403
A. occidentale	-45,416667	-11,233333
A. occidentale	-45,4667	-11,1214
A. occidentale	-45,51527778	-11,0225
A. occidentale	-45,5175	-11,1172
A. occidentale	-45,539583	-11,515936
A. occidentale	-45,666667	-7,383333
A. occidentale	-45,72	-1,44
A. occidentale	-45,720008	-12,619883
A. occidentale	-45,778611	-2,485639
A. occidentale	-45,8917809	-1,8926301
A. occidentale	-45,899417	-9,179979
A. occidentale	-45,9	-11,339722
A. occidentale	-45,900469	-9,000173
A. occidentale	-45,92055556	-15,40555556
A. occidentale	-45,938086	-3,166032
A. occidentale	-45,972667	-5,868078
A. occidentale	-45,989286	-8,908025
A. occidentale	-46,05	-5,0667
A. occidentale	-46,079538	-8,999972
A. occidentale	-46,08	-3,12
A. occidentale	-46,18594444	-0,92333333
A. occidentale	-46,1955	-0,91652778
A. occidentale	-46,275555	-12,507222
A. occidentale	-46,326666	-14,023611

Species	Longitude	Latitude
A. occidentale	-46,351944	-13,768888
A. occidentale	-46,380555	-6,9477777
A. occidentale	-46,381944	-13,882777
A. occidentale	-46,39111111	-10,55138889
A. occidentale	-46,4	-13,316667
A. occidentale	-46,40000153	-24
A. occidentale	-46,424722	-10,576944
A. occidentale	-46,61711111	-0,83813889
A. occidentale	-46,619444	-9,179444
A. occidentale	-46,65	-10,55
A. occidentale	-46,651666	-13,1925
A. occidentale	-46,66666667	-0,91552778
A. occidentale	-46,66666667	-0,88333333
A. occidentale	-46,66744444	-0,92202778
A. occidentale	-46,68166667	-0,915
A. occidentale	-46,68297222	-0,91722222
A. occidentale	-46,683333	-1,1
A. occidentale	-46,688611	-10,197778
A. occidentale	-46,7	-1,183333
A. occidentale	-46,716666	-14,116666
A. occidentale	-46,75	-13
A. occidentale	-46,769444	-13,168333
A. occidentale	-46,770555	-14,511388
A. occidentale	-46,772778	-11,094444
A. occidentale	-46,772778	-11,111111
A. occidentale	-46,780277	-13,173888
A. occidentale	-46,791111	-10,960556
A. occidentale	-46,799722	-13,021666
A. occidentale	-46,8	-6,120556
A. occidentale	-46,8025	-13,227777
A. occidentale	-46,839722	-12,995833
A. occidentale	-46,869722	-23,869722
A. occidentale	-46,916666	-13,116666
A. occidentale	-46,926111	-12,945833
A. occidentale	-46,953055	-9,5166666
A. occidentale	-46,991317	-13,952333
A. occidentale	-46,999166	-12,881666
A. occidentale	-47,01027778	-13,87777778
A. occidentale	-47,09305556	-0,69638889
A. occidentale	-47,129444	-11,115277
A. occidentale	-47,144722	-19,211667
A. occidentale	-47,159988	-7,740004
A. occidentale	-47,16	-19,233611
A. occidentale	-47,16	-12,24
A. occidentale	-47,16	-10,08
A. occidentale	-47,16	-12,78
A. occidentale	-47,203333	-12,806111
A. occidentale	-47,206944	-12,483611

Species	Longitude	Latitude
A. occidentale	-47,211111	-11,593611
A. occidentale	-47,216666	-13,7
A. occidentale	-47,217611	-13,593194
A. occidentale	-47,24309921	-8,727780342
A. occidentale	-47,25	-13,83
A. occidentale	-47,284722	-7,68
A. occidentale	-47,375555	-11,631111
A. occidentale	-47,383333	-13,783333
A. occidentale	-47,433333	-13,783333
A. occidentale	-47,5	-13,5
A. occidentale	-47,5	-6,5
A. occidentale	-47,52561	-11,44245
A. occidentale	-47,53575	-11,145
A. occidentale	-47,54999924	-24,70000076
A. occidentale	-47,55	-15,533333
A. occidentale	-47,5652	-11,1914
A. occidentale	-47,574722	-10,666667
A. occidentale	-47,58190155	-0,595000029
A. occidentale	-47,605	-11,725277
A. occidentale	-47,61666667	-22,7
A. occidentale	-47,61999893	-22,21999931
A. occidentale	-47,631667	-11,970833
A. occidentale	-47,6339	-13,8847
A. occidentale	-47,665556	-8,4475
A. occidentale	-47,665556	-8,433611
A. occidentale	-47,66999817	-22,84000015
A. occidentale	-47,708333	-15,591666
A. occidentale	-47,765836	-1,675947
A. occidentale	-47,778333	-1,916667
A. occidentale	-47,816667	-15,616667
A. occidentale	-47,8319	-14,1831
A. occidentale	-47,846388	-13,673055
A. occidentale	-47,866667	-0,616667
A. occidentale	-47,869722	-5,370833
A. occidentale	-47,877778	-15,947222
A. occidentale	-47,88	-16,56
A. occidentale	-47,88225	-11,740139
A. occidentale	-47,936111	-16,780833
A. occidentale	-47,9475	-16,7825
A. occidentale	-47,9475	-11,811944
A. occidentale	-47,947778	-11,320556
A. occidentale	-48	-12
A. occidentale	-48,05	-15,55
A. occidentale	-48,059167	-13,565
A. occidentale	-48,069472	-13,596333
A. occidentale	-48,072222	-15,683888
A. occidentale	-48,076944	-11,456666
A. occidentale	-48,085278	-13,5875

Species	Longitude	Latitude
A. occidentale	-48,095	-14,196666
A. occidentale	-48,112777	-12,996388
A. occidentale	-48,113611	-13,406667
A. occidentale	-48,116389	-10,442778
A. occidentale	-48,137778	-13,486944
A. occidentale	-48,167778	-12,144167
A. occidentale	-48,18	-15,58
A. occidentale	-48,2	-13,666667
A. occidentale	-48,201388	-11,247222
A. occidentale	-48,216667	-15,283333
A. occidentale	-48,216667	-10
A. occidentale	-48,2275	-12,8
A. occidentale	-48,233333	-13,583333
A. occidentale	-48,236667	-10,358056
A. occidentale	-48,238889	-12,788889
A. occidentale	-48,24	-7,38
A. occidentale	-48,24	-7,74
A. occidentale	-48,25	-15,5
A. occidentale	-48,262778	-10,42
A. occidentale	-48,266389	-10,351667
A. occidentale	-48,28170013	-0,936667025
A. occidentale	-48,283333	-14,016667
A. occidentale	-48,288611	-14,267778
A. occidentale	-48,3	-13,583333
A. occidentale	-48,306111	-12,904722
A. occidentale	-48,310806	-10,414056
A. occidentale	-48,316666	-13,966666
A. occidentale	-48,323611	-13,155555
A. occidentale	-48,333333	-10,666667
A. occidentale	-48,366666	-15,1
A. occidentale	-48,366666	-9,5833333
A. occidentale	-48,366667	-9,583333
A. occidentale	-48,366667	-15,1
A. occidentale	-48,367867	-10,8808
A. occidentale	-48,41666	-1,66666
A. occidentale	-48,418972	-1,17375
A. occidentale	-48,433333	-6,233333
A. occidentale	-48,433333	-9,55
A. occidentale	-48,433333	-10,1
A. occidentale	-48,433611	-1,431944
A. occidentale	-48,45	-14,496111
A. occidentale	-48,473333	-1,5
A. occidentale	-48,487056	-0,663333
A. occidentale	-48,496389	-16,149444
A. occidentale	-48,50583333	-15,46055556
A. occidentale	-48,507464	-0,667394
A. occidentale	-48,514722	-14,5625
A. occidentale	-48,5275	-12,959722

Species	Longitude	Latitude
A. occidentale	-48,5275	-16,549444
A. occidentale	-48,533333	-9,4166666
A. occidentale	-48,537222	-11,121389
A. occidentale	-48,563333	-27,8325
A. occidentale	-48,566666	-9,4
A. occidentale	-48,566666	-14,466666
A. occidentale	-48,583333	-15,5
A. occidentale	-48,584167	-16,423611
A. occidentale	-48,6	-9,54
A. occidentale	-48,613836	-1,52225
A. occidentale	-48,616667	-15,95
A. occidentale	-48,648889	-11,913611
A. occidentale	-48,666666	-16,05
A. occidentale	-48,667222	-10,641944
A. occidentale	-48,683333	-9,45
A. occidentale	-48,683333	-15,7
A. occidentale	-48,702777	-15,341944
A. occidentale	-48,772806	-11,77575
A. occidentale	-48,78375	-6,510111
A. occidentale	-48,8082	-25,5105
A. occidentale	-48,824444	-15,800278
A. occidentale	-48,83	-15,67
A. occidentale	-48,8769445	-14,5544444
A. occidentale	-48,883333	-10,15
A. occidentale	-48,9	-9,55
A. occidentale	-48,966667	-14,633333
A. occidentale	-48,983333	-9,2833333
A. occidentale	-48,988889	-15,830278
A. occidentale	-48,993139	-15,822639
A. occidentale	-49,033333	-14,666667
A. occidentale	-49,047222	-15,738889
A. occidentale	-49,05	-9,266667
A. occidentale	-49,05	-16,133333
A. occidentale	-49,05	-14,733333
A. occidentale	-49,05	-14,633333
A. occidentale	-49,066666	-14,616666
A. occidentale	-49,26	-15,7
A. occidentale	-49,274444	-14,200278
A. occidentale	-49,278333	-14,196944
A. occidentale	-49,3163889	-20,7080556
A. occidentale	-49,316667	-15,6
A. occidentale	-49,333333	-15,8
A. occidentale	-49,3715662	-3,7710406
A. occidentale	-49,487731	-16,352778
A. occidentale	-49,49000168	-21,01000023
A. occidentale	-49,507175	-16,371836
A. occidentale	-49,6395457	-1,6123151
A. occidentale	-49,771667	-12,561667

Species	Longitude	Latitude
A. occidentale	-49,87829971	-6,496940136
A. occidentale	-50,3166667	-5,9833333
A. occidentale	-50,390833	-6,355833
A. occidentale	-50,440861	-2,014247
A. occidentale	-50,5	-10,416667
A. occidentale	-50,73	-12,04
A. occidentale	-50,78555556	-12,955
A. occidentale	-50,92269444	-12,30986111
A. occidentale	-51,033333	0,15
A. occidentale	-51,06639862	-0,366667002
A. occidentale	-51,07152778	-13,02402778
A. occidentale	-51,07775	-13,03127778
A. occidentale	-51,0846984	0,3138598
A. occidentale	-51,12	-13,14
A. occidentale	-51,15	-15,083333
A. occidentale	-51,2785	-16,114111
A. occidentale	-51,286389	-16,105556
A. occidentale	-51,333333	0,63333333
A. occidentale	-51,365639	-0,2385
A. occidentale	-51,523333	-3,222056
A. occidentale	-51,66	-14,58
A. occidentale	-51,692333	-3,007056
A. occidentale	-51,75	-0,8333333
A. occidentale	-51,766667	-12,816667
A. occidentale	-51,77	-17,35
A. occidentale	-51,866667	-12,9
A. occidentale	-52	0,71666666
A. occidentale	-52,0510769	-1,5964373
A. occidentale	-52,065472	-23,726444
A. occidentale	-52,18	-15,47
A. occidentale	-52,183333	-15,316667
A. occidentale	-52,23	-15,28
A. occidentale	-52,33	-14,73
A. occidentale	-52,38	-14,94
A. occidentale	-52,38	-16,02
A. occidentale	-52,43333333	-14,53333333
A. occidentale	-52,448709	-3,864276
A. occidentale	-52,57	-13,18
A. occidentale	-52,57	-13,33
A. occidentale	-52,714444	-17,881389
A. occidentale	-52,8731	-10,8339
A. occidentale	-53,2	3,62
A. occidentale	-53,2175	-17,301944
A. occidentale	-53,8	5,61
A. occidentale	-54,0833333	-1,9666667
A. occidentale	-54,135167	5,4035
A. occidentale	-54,25	-1,916667
A. occidentale	-54,516667	-1,666667

Species	Longitude	Latitude
A. occidentale	-54,531056	-2,315083
A. occidentale	-54,650833	4,127222
A. occidentale	-54,72	-19,8
A. occidentale	-54,798333	-2,445
A. occidentale	-54,9	-9,366667
A. occidentale	-54,932778	-2,500833
A. occidentale	-54,936278	-2,502611
A. occidentale	-54,95	-2,483333
A. occidentale	-54,957778	-2,494722
A. occidentale	-54,960964	-2,593669
A. occidentale	-55	-9
A. occidentale	-55,055833	-2,3377777
A. occidentale	-55,070278	-2,89
A. occidentale	-55,1346	-9,0408
A. occidentale	-55,1833333	5,4166667
A. occidentale	-55,2	5,45
A. occidentale	-55,229367	-9,668978
A. occidentale	-55,479444	-10,980278
A. occidentale	-55,551939	-12,251203
A. occidentale	-55,583333	-15,5
A. occidentale	-55,697694	-10,817028
A. occidentale	-55,72	3,17
A. occidentale	-55,784722	-10,898889
A. occidentale	-55,8575	-1,826111
A. occidentale	-55,9530983	-10,55809975
A. occidentale	-56	-11
A. occidentale	-56,155833	-13,043056
A. occidentale	-56,183333	-15,316667
A. occidentale	-56,41	-14,753056
A. occidentale	-56,5	3,6
A. occidentale	-56,6558075	-4,7009877
A. occidentale	-56,7333333	-15,8166667
A. occidentale	-56,813953	-10,349608
A. occidentale	-57	-14,363333
A. occidentale	-57	-12
A. occidentale	-57,010528	-14,363417
A. occidentale	-57,22750092	-15,32439995
A. occidentale	-57,5333	5,71667
A. occidentale	-57,558278	-16,555056
A. occidentale	-57,560639	-16,566694
A. occidentale	-57,5833	5,61667
A. occidentale	-57,599722	-13,32
A. occidentale	-57,6667	5,55
A. occidentale	-57,679861	-16,58
A. occidentale	-58	-3
A. occidentale	-58,010694	-16,145556
A. occidentale	-58,1667	6,5
A. occidentale	-58,1833	6,5

Species	Longitude	Latitude
A. occidentale	-58,21	6,45
A. occidentale	-58,2333	6,3
A. occidentale	-58,2433	6,38472
A. occidentale	-58,25	6,3333333
A. occidentale	-58,25	6,41667
A. occidentale	-58,35	5,61
A. occidentale	-58,3542	6,5775
A. occidentale	-58,35889816	-10,31809998
A. occidentale	-58,6167	6,53333
A. occidentale	-58,65	6,4
A. occidentale	-58,67	-14,92
A. occidentale	-58,7017	4,7364
A. occidentale	-58,71	7,25
A. occidentale	-58,74110031	-11,37829971
A. occidentale	-58,86	7,61
A. occidentale	-58,9808	5,34139
A. occidentale	-59	5,36
A. occidentale	-59,066667	2,066667
A. occidentale	-59,0758	2,06778
A. occidentale	-59,08	7,75
A. occidentale	-59,1478	7,55944
A. occidentale	-59,16	3,75
A. occidentale	-59,3133	5,30028
A. occidentale	-59,35	4
A. occidentale	-59,5	5,33
A. occidentale	-59,583333	-4,383333
A. occidentale	-59,66666	8,33333
A. occidentale	-59,7	7,366667
A. occidentale	-59,86	3,56
A. occidentale	-59,883333	3,58333333
A. occidentale	-59,9	5,95
A. occidentale	-59,907806	-15,082306
A. occidentale	-59,98	8,16
A. occidentale	-60	-2
A. occidentale	-60	8,16666
A. occidentale	-60,033333	-2,6
A. occidentale	-60,083333	-2,316667
A. occidentale	-60,0917441	-2,326638
A. occidentale	-60,1	3,25
A. occidentale	-60,21361111	-3,08944444
A. occidentale	-60,3	3,08333333
A. occidentale	-60,30805556	-3,04972222
A. occidentale	-60,416667	2,883333
A. occidentale	-60,41809845	0,946111023
A. occidentale	-60,833333	2,383333
A. occidentale	-60,833333	2,46666666
A. occidentale	-60,916667	3,15
A. occidentale	-60,92694	-14,55555

Species	Longitude	Latitude
A. occidentale	-60,983333	2,983333
A. occidentale	-60,983333	3,483333
A. occidentale	-60,983333	2,41666666
A. occidentale	-61	-13,6
A. occidentale	-61,0847	5,85222
A. occidentale	-61,183333	2,7
A. occidentale	-61,278056	-5,802222
A. occidentale	-61,466667	2,833333
A. occidentale	-61,557577	-0,794911
A. occidentale	-61,583333	-1,833333
A. occidentale	-61,608958	-11,429928
A. occidentale	-61,816667	-1,666667
A. occidentale	-61,88333	6,21666
A. occidentale	-61,92	-3,6
A. occidentale	-61,935725	-6,385186
A. occidentale	-62,233333	-1,766667
A. occidentale	-62,557378	-4,238767
A. occidentale	-63,166667	-7,516667
A. occidentale	-63,4166667	-17,6833333
A. occidentale	-63,6956	-8,80972
A. occidentale	-63,91666	8,73333
A. occidentale	-64,0063889	9,9472222
A. occidentale	-64,11666	4,45
A. occidentale	-64,41	6,23
A. occidentale	-64,435	-16,7261
A. occidentale	-64,44	-9,54
A. occidentale	-64,5	7,5
A. occidentale	-64,62	-9,36
A. occidentale	-65,1	-2,733333
A. occidentale	-65,1333333	7,4
A. occidentale	-65,9119	7,0758
A. occidentale	-66,6166667	-14,5
A. occidentale	-67	1,833333
A. occidentale	-67,05	7,16666
A. occidentale	-67,05	1,91666
A. occidentale	-67,06666	1,91666
A. occidentale	-67,114609	2,003681
A. occidentale	-67,2375	3,6780556
A. occidentale	-67,5	7,166667
A. occidentale	-67,721111	-11,731111
A. occidentale	-67,75	4,08
A. occidentale	-67,833333	1,916667
A. occidentale	-67,86	3,78
A. occidentale	-67,88333	-14,45
A. occidentale	-68,75	6,16666
A. occidentale	-68,8133333	-12,4833333
A. occidentale	-68,98027	-12,51027
A. occidentale	-69,05	-12,48333

Species	Longitude	Latitude
A. occidentale	-69,08305	-12,52805
A. occidentale	-69,28	-12,83
A. occidentale	-69,3	-12,81666
A. occidentale	-69,733333	-1,283333
A. occidentale	-69,742222	-1,466111
A. occidentale	-69,76	-2,91
A. occidentale	-69,95	-4,05
A. occidentale	-69,973265	6,10193
A. occidentale	-70,0377778	-4,3683056
A. occidentale	-70,08	1,25
A. occidentale	-70,119796	-4,023249
A. occidentale	-70,236106	1,26932
A. occidentale	-70,25	-3,7833333
A. occidentale	-70,39	1,35
A. occidentale	-70,40545	-3,57774
A. occidentale	-70,42319	1,00642
A. occidentale	-70,5	-0,33
A. occidentale	-70,5	0,083333
A. occidentale	-70,66666	0,5
A. occidentale	-70,666667	-0,5
A. occidentale	-71,28594	1,217802
A. occidentale	-71,307975	4,81288
A. occidentale	-72	-1
A. occidentale	-72,31	-0,65
A. occidentale	-72,32	-0,65
A. occidentale	-72,33	-0,61
A. occidentale	-72,35	-0,61
A. occidentale	-72,366669	-0,583333
A. occidentale	-72,39	-0,59
A. occidentale	-72,41	-0,6
A. occidentale	-72,55	9,43333
A. occidentale	-72,633494	8,080919
A. occidentale	-72,64	-1,33
A. occidentale	-72,71	-1,53
A. occidentale	-72,75	-1,41
A. occidentale	-72,833336	9,152778
A. occidentale	-72,8667	3,8811
A. occidentale	-72,94022	4,10509
A. occidentale	-73	8
A. occidentale	-73,01577	4,02421
A. occidentale	-73,183558	7,101636
A. occidentale	-73,2333333	-3,7333333
A. occidentale	-73,2666667	-3,8333333
A. occidentale	-73,28333	-3,66666
A. occidentale	-73,3233334	-3,8286111
A. occidentale	-73,33333	-3,75
A. occidentale	-73,5	-3,8333333
A. occidentale	-73,55	-4,5333333

Species	Longitude	Latitude
A. occidentale	-73,5672223	-3,885
A. occidentale	-73,76	2,25
A. occidentale	-74,13527	7,4233
A. occidentale	-74,14	-9,1839
A. occidentale	-74,147494	7,399346
A. occidentale	-74,2430555	-5,1283334
A. occidentale	-74,25	6,9166667
A. occidentale	-74,5	10
A. occidentale	-74,708996	5,547049
A. occidentale	-74,9728	8,1761
A. occidentale	-75	-4
A. occidentale	-75,0833333	8,0333333
A. occidentale	-75,09834	8,34789
A. occidentale	-75,15	-10,1922222
A. occidentale	-75,183334	2,866667
A. occidentale	-75,21666	-10,2
A. occidentale	-75,25	-10,31666
A. occidentale	-75,28987	6,4619
A. occidentale	-75,3922222	-10,1519444
A. occidentale	-75,3941	7,288114
A. occidentale	-75,45605	1,083517
A. occidentale	-75,5	5,25
A. occidentale	-75,5716667	8,2730556
A. occidentale	-75,597435	4,988008
A. occidentale	-75,606517	1,6641
A. occidentale	-75,61	1,65
A. occidentale	-75,65611	1,499944
A. occidentale	-75,67	1,62
A. occidentale	-75,69	1,63
A. occidentale	-75,706389	1,682778
A. occidentale	-75,74	1,43
A. occidentale	-75,8333333	-3,25
A. occidentale	-76,300003	0,416667
A. occidentale	-76,5	3,75
A. occidentale	-76,54485	5,746797
A. occidentale	-77,116667	8,383333
A. occidentale	-77,75	8,25
A. occidentale	-78,1869	-1,4628
A. occidentale	-78,96666	9,26666
A. occidentale	-79,36666	9,23333
A. occidentale	-79,4	9,16666
A. occidentale	-79,47638	0,34722
A. occidentale	-79,55	8,78333
A. occidentale	-79,616667	8,916667
A. occidentale	-79,65	9,46666
A. occidentale	-79,664444	9,046667
A. occidentale	-79,66666	9,15
A. occidentale	-79,67555	9,08916

Species	Longitude	Latitude
A. occidentale	-79,733333	9,366667
A. occidentale	-79,78333	9,33333
A. occidentale	-79,80833	9,23472
A. occidentale	-79,82416	9,15194
A. occidentale	-79,83722	9,16305
A. occidentale	-79,84361	9,17805
A. occidentale	-79,84972	8,74027
A. occidentale	-79,87388	8,71194
A. occidentale	-79,9	8,683333
A. occidentale	-80,1	9,15
A. occidentale	-80,136111	7,736111
A. occidentale	-80,29166	8,41166
A. occidentale	-82,24666	9,195
A. occidentale	-82,42333	8,60166
A. occidentale	-83,13	10,03
A. occidentale	-83,14	8,61
A. occidentale	-83,141667	8,616667
A. occidentale	-83,21	8,68
A. occidentale	-83,308333	9,211111
A. occidentale	-83,380556	8,75
A. occidentale	-83,52	8,61
A. occidentale	-83,581028	10,438444
A. occidentale	-83,65	9,9
A. occidentale	-83,67	8,59
A. occidentale	-83,68333	14,75
A. occidentale	-83,716667	8,616667
A. occidentale	-83,73	10,64
A. occidentale	-83,736111	10,643889
A. occidentale	-83,741667	12,225
A. occidentale	-83,746111	12,223889
A. occidentale	-83,857778	10,191111
A. occidentale	-83,945833	9,5
A. occidentale	-83,955556	9,5
A. occidentale	-83,972222	9,416667
A. occidentale	-84,03	9,53
A. occidentale	-84,0875	9,55
A. occidentale	-84,1	14,733333
A. occidentale	-84,13	9,45
A. occidentale	-84,21666	15
A. occidentale	-84,21666	11,5
A. occidentale	-84,258333	13,966667
A. occidentale	-84,26666	15,03333
A. occidentale	-84,3	11,93
A. occidentale	-84,39	9,71
A. occidentale	-84,398611	9,719444
A. occidentale	-84,46666	11,03333
A. occidentale	-84,5	9,884722
A. occidentale	-84,56	9,95

Species	Longitude	Latitude
A. occidentale	-84,583333	9,75
A. occidentale	-84,91	10,16
A. occidentale	-84,93333	9,78333
A. occidentale	-85,058333	13,741667
A. occidentale	-85,202778	10,480556
A. occidentale	-85,216667	13,3
A. occidentale	-85,31	10,4
A. occidentale	-85,316667	10,4
A. occidentale	-85,318056	10,440278
A. occidentale	-85,328743	10,392593
A. occidentale	-85,354532	10,456707
A. occidentale	-85,430278	14,1
A. occidentale	-85,450278	10,665278
A. occidentale	-85,474858	10,889386
A. occidentale	-85,48	10,63
A. occidentale	-85,67617	10,804516
A. occidentale	-85,8	11,46666
A. occidentale	-85,90833	15,95833
A. occidentale	-86,13333	11,96666
A. occidentale	-86,261667	13,385278
A. occidentale	-86,3	13,63333
A. occidentale	-86,322222	13,193056
A. occidentale	-87,075278	14,025278
A. occidentale	-87,1	14,2
A. occidentale	-87,164444	14,129722
A. occidentale	-88,31444	16,7975
A. occidentale	-88,32611	17,66055
A. occidentale	-88,53333	17,78333
A. occidentale	-88,55	17,36666
A. occidentale	-88,55222	17,35055
A. occidentale	-88,66666	17,08333
A. occidentale	-88,684767	17,217668
A. occidentale	-88,949997	13,88333
A. occidentale	-88,973056	16,983056
A. occidentale	-90,10027	16,79861
A. occidentale	-90,386111	19,809722
A. occidentale	-90,484167	19,761389
A. occidentale	-90,658333	18,791667
A. occidentale	-90,85	14,8
A. occidentale	-90,916667	18,483333
A. occidentale	-91,823611	17,633611
A. occidentale	-91,891111	18,618611
A. occidentale	-92,395278	15,006111
A. occidentale	-92,460278	17,7775
A. occidentale	-92,628333	15,254444
A. occidentale	-93,288333	15,984167
A. occidentale	-93,730278	17,980556
A. occidentale	-93,752778	16,691667

**Species**	**Longitude**	**Latitude**
A. occidentale	-93,833333	17,833333
A. occidentale	-93,906389	16,353611
A. occidentale	-93,98333333	18,18333333
A. occidentale	-94,109722	17,955
A. occidentale	-94,638333	18,175
A. occidentale	-95,0025	16,868639
A. occidentale	-95,241667	16,991667
A. occidentale	-96,383333	19,266667
A. occidentale	-96,508333	15,808333
A. occidentale	-98,235	16,79
