# Supplementary material for: Insights into the evolutionary history of the most skilled tool-handling platyrrhini monkey: Sapajus libidinosus from the Serra da Capivara National Park
Source: Genet Mol Biol. 2023 Nov 10;46(3 Suppl 1):e20230165. doi: 10.1590/1678-4685-GMB-2023-0165 (PMC10637428; doi:10.1590/1678-4685-GMB-2023-0165)
Supplement: Table S7 - [file 1415-4757-GMB-46-3-s1-e20230165-s7.pdf]

Genetics and Molecular Biology

https://doi.org/10.1590/1678-4685-GMB-2023-0165

Supplementary Material to "Insights into the evolutionary history of the most skilled tool-handling platyrrhini monkey: S*apajus libidinosus* from the Serra da Capivara National Park"

Table S7 - Occurence data of *Attalea speciosa* used for the Species Distribution Modeling.

**Species**	**Longitude**	**Latitude**
A. speciosa	-49,333333	-5,833333
A. speciosa	-47,604167	-11,736389
A. speciosa	-47,601944	-11,729167
A. speciosa	-40,793333	-11,733889
A. speciosa	-60,991	-15,155
A. speciosa	-65,33	-10,75
A. speciosa	-64,75	-12,66666
A. speciosa	-40,75	-4,333333
A. speciosa	-62	-16
A. speciosa	-36,308111	-9,703133
A. speciosa	-40,855278	-11,890833
A. speciosa	-43,92	-5,219722
A. speciosa	-46,98	-5,58
A. speciosa	-47,381964	-6,945275
A. speciosa	-48,824508	-15,778408
A. speciosa	-50,216667	-5,516667
A. speciosa	-55,4833333	-20,4555556
A. speciosa	-56,772222	-9,336667
A. speciosa	-57,22750092	-15,32439995
A. speciosa	-64,628611	-9,849167
A. speciosa	-65,7	-9,86666
A. speciosa	-43,261111	-4,895
A. speciosa	-44,333333	-3,4166666
A. speciosa	-44,833333	-16,5
A. speciosa	-44,9	-6,9
A. speciosa	-44,916666	-6,1666666
A. speciosa	-45,498611	-3,7769444
A. speciosa	-45,5	-3,5
A. speciosa	-45,938086	-3,166032
A. speciosa	-46,08	-3,12
A. speciosa	-46,083333	-3,6666666
A. speciosa	-46,083333	-7,583333

Species	Longitude	Latitude
A. speciosa	-46,62	-5,94
A. speciosa	-46,66833333	-0,92611111
A. speciosa	-46,91666667	-1,08333333
A. speciosa	-47,466666	-6,3333333
A. speciosa	-47,599167	-11,731944
A. speciosa	-47,83	-7,5
A. speciosa	-48,066666	-14,133333
A. speciosa	-48,166666	-6,6666666
A. speciosa	-48,316666	-13,966666
A. speciosa	-49,128194	-14,103583
A. speciosa	-49,333333	-5,0833333
A. speciosa	-50,29	-6,14
A. speciosa	-50,48333333	-6,23333333
A. speciosa	-50,555833	-15,1825
A. speciosa	-51,75	-6,75
A. speciosa	-52,5854588	-7,2040035
A. speciosa	-56,166667	2,166667
A. speciosa	-57,33333333	3,33333333
A. speciosa	-60,95	-15,63
A. speciosa	-62,25	-10,666667
A. speciosa	-62,57	-16,17
A. speciosa	-63,13	-16
A. speciosa	-65,057777	-9,6033333
A. speciosa	-65,080475	-10,838156
A. speciosa	-65,7	-10,63
A. speciosa	-66,13	-11,33
A. speciosa	-60,0520336	-2,6502916
A. speciosa	-62,2449896	-10,7175357
A. speciosa	-44,834953	-7,074903
A. speciosa	-55,634178	-4,238908
A. speciosa	-44,3652958	-2,7942148
A. speciosa	-60,74161	-3,342168
A. speciosa	-46,860874	-1,069934
A. speciosa	-64,683199	-15,064464
A. speciosa	-64,664561	-13,223964
