# Supplementary material for: Insights into the evolutionary history of the most skilled tool-handling platyrrhini monkey: Sapajus libidinosus from the Serra da Capivara National Park
Source: Genet Mol Biol. 2023 Nov 10;46(3 Suppl 1):e20230165. doi: 10.1590/1678-4685-GMB-2023-0165 (PMC10637428; doi:10.1590/1678-4685-GMB-2023-0165)
Supplement: Table S8 - [file 1415-4757-GMB-46-3-s1-e20230165-s8.pdf]

Genetics and Molecular Biology

https://doi.org/10.1590/1678-4685-GMB-2023-0165

Supplementary Material to “Insights into the evolutionary history of the most skilled tool-handling platyrrhini monkey: S*apajus libidinosus* from the Serra da Capivara National Park”

Table S8 - Occurence data of *Ficus gomelleria* used for the Species Distribution Modeling.

**Species**	**Longitude**	**Latitude**
F. gomelleira	-41,70920181	-3,928060055
F. gomelleira	-57,7633472	-16,041553
F. gomelleira	-58,8258722	-9,81975
F. gomelleira	-75,768611	1,737028
F. gomelleira	-46,6670194	-9,26083
F. gomelleira	-44,40825	-18,644444
F. gomelleira	-43,403611	-22,939722
F. gomelleira	-48,8953306	-2,947314
F. gomelleira	-41,742306	-3,359028
F. gomelleira	-39,338056	-17,380278
F. gomelleira	-41,7969444	-21,8469444
F. gomelleira	-43,86169815	-16,73500061
F. gomelleira	-48,63809967	-26,24329948
F. gomelleira	-61,416667	3,366667
F. gomelleira	-39,73220062	-18,59329987
F. gomelleira	-56,907222	-2,199639
F. gomelleira	-41,77690125	-4,273330212
F. gomelleira	-49,37559891	-26,95529938
F. gomelleira	-46,1385994	-23,85440063
F. gomelleira	-56,801667	-9,243611
F. gomelleira	-45,5155556	-21,0813889
F. gomelleira	-40,36970139	-19,83189964
F. gomelleira	-46,3658	-20,2453
F. gomelleira	-57,679861	-16,58
F. gomelleira	-57,509028	-16,820972
F. gomelleira	-57,071633	-9,229156
F. gomelleira	-48,32889938	-25,30669975
F. gomelleira	-40,191167	-19,473056
F. gomelleira	-55,5966667	-19,85
F. gomelleira	-43,197639	-20,281056
F. gomelleira	-39,900833	-19,498444
F. gomelleira	-40,0722	-19,3911

Species	Longitude	Latitude
F. gomelleira	-55,449947	-11,247614
F. gomelleira	-40,945919	-9,586922
F. gomelleira	-41,33333	-11,15
F. gomelleira	-41,4480555	-21,7972222
F. gomelleira	-44,04059982	-22,95969963
F. gomelleira	-41,97330093	-21,04220009
F. gomelleira	-39,374167	-15,262778
F. gomelleira	-52,35309982	-14,67329979
F. gomelleira	-44,881194	-23,313667
F. gomelleira	-48,5746994	-25,88279915
F. gomelleira	-49,729111	-22,465083
F. gomelleira	-57,047222	-9,198889
F. gomelleira	-44,7580986	-21,03310013
F. gomelleira	-41,17607116	-10,04484855
F. gomelleira	-49,1425	-26,964444
F. gomelleira	-39,14310074	-7,249720097
F. gomelleira	-43,455305	-8,906055
F. gomelleira	-40,210556	-14,331667
F. gomelleira	-40,192778	-14,408333
F. gomelleira	-40,227222	-14,356667
F. gomelleira	-40,228333	-14,329444
F. gomelleira	-45,23690033	-19,33169937
F. gomelleira	-57,67890167	-16,07060051
F. gomelleira	-57,75279999	-6,222219944
F. gomelleira	-55,78720093	-20,47109985
F. gomelleira	-53,20529938	-17,32559967
F. gomelleira	-44,5	-14,083333
F. gomelleira	-40,8260994	-3,120280027
F. gomelleira	-61,276906	2,548772
F. gomelleira	-36,266667	-10,000556
F. gomelleira	-39,9821641	-18,40637
F. gomelleira	-48,576306	-25,567672
F. gomelleira	-44,850201	-23,348101
F. gomelleira	-44,999444	-21,245278
F. gomelleira	-56,47669983	-9,664719582
F. gomelleira	-74,16667	2,6667
F. gomelleira	-35,561108	-9,3333
F. gomelleira	-42,73220062	-19,71220016
F. gomelleira	-45,5	-23,633333
F. gomelleira	-41,53310013	-20,7635994
F. gomelleira	-39,004722	-15,299167
F. gomelleira	-41,1894583	-9,735583
F. gomelleira	-41,788669	-10,246361
F. gomelleira	-73,4814	4,0958
F. gomelleira	-36,17559814	-10,12559986
F. gomelleira	-37,870847	-9,556405
F. gomelleira	-56	-9
F. gomelleira	-47,54999924	-24,70000076

Species	Longitude	Latitude
F. gomelleira	-43,35029984	-21,76420021
F. gomelleira	-51,67829895	-20,75110054
F. gomelleira	-36,601944	-10,003333
F. gomelleira	-42,491944	-20,237778
F. gomelleira	-35,95669937	-9,597220421
F. gomelleira	-42,153583	-5,02735
F. gomelleira	-41,330833	-21,184167
F. gomelleira	-41,03939819	-5,865560055
F. gomelleira	-48,07778	-14,07778
F. gomelleira	-40,316667	-10,5
F. gomelleira	-44,995347	-13,421928
F. gomelleira	-42,88190079	-20,75390053
F. gomelleira	-43,191667	-22,041667
F. gomelleira	-48,15	-14,11667
F. gomelleira	-40,581667	-19,950278
F. gomelleira	-40,540556	-20,278333
F. gomelleira	-48,59000015	-24,57999992
F. gomelleira	-44,08140182	-19,55780029
F. gomelleira	-48,064444	-25,0025
F. gomelleira	-72,46667	2,5
F. gomelleira	-36,24079895	-9,371390343
F. gomelleira	-41,09220123	-3,562220097
F. gomelleira	-47,35	-15,633333
F. gomelleira	-42,905278	-22,493333
F. gomelleira	-41,04669952	-21,09889984
F. gomelleira	-42,716667	-11,1075
F. gomelleira	-43,01060104	-21,54000092
F. gomelleira	-36,38809967	-9,447500229
F. gomelleira	-48,66189957	-26,90780067
F. gomelleira	-56,56175	-15,555833
F. gomelleira	-56,09669876	-15,59609985
F. gomelleira	-45,070833	-23,433889
F. gomelleira	-53,916667	-10,666667
F. gomelleira	-42,1310997	-7,025279999
F. gomelleira	-70,22944	-2,50394
F. gomelleira	-57,0433333	-19,4647222
F. gomelleira	-40,441417	-18,763917
F. gomelleira	-70,18	-2,5
F. gomelleira	-40,383056	-19,698889
F. gomelleira	-35,85329819	-9,478329659
F. gomelleira	-48,50920105	-25,52000046
F. gomelleira	-51,205278	-16,407222
F. gomelleira	-48,61610031	-26,11689949
F. gomelleira	-36,561075	-9,063867
F. gomelleira	-46,81361	-13,6225
F. gomelleira	-41,65	-13,583333
F. gomelleira	-41,55889893	-12,52890015
F. gomelleira	-46,85056	-14,48806

Species	Longitude	Latitude
F. gomelleira	-42,23889923	-13,44250011
F. gomelleira	-47,31000137	-22,94000053
F. gomelleira	-52,916667	-0,916667
F. gomelleira	-49,1144444	-26,5430555
F. gomelleira	-43,36220169	-19,8260994
F. gomelleira	-48,149166	-12,933055
F. gomelleira	-48,221111	-12,911111
F. gomelleira	-48,7118988	-25,42860031
F. gomelleira	-48,83440018	-25,4769001
F. gomelleira	-56,8333333	-18,7666667
F. gomelleira	-47,63000107	-24,31999969
F. gomelleira	-39,7075	-14,181389
F. gomelleira	-73,563472	1,664028
F. gomelleira	-72,863222	1,955472
F. gomelleira	-73,04	2,01
F. gomelleira	-39,721667	-13,595278
F. gomelleira	-38,733889	-10,956111
F. gomelleira	-72,866403	2,526128
F. gomelleira	-47,876944	-24,608333
F. gomelleira	-41,366667	-13,083333
F. gomelleira	-40,490556	-12,318611
F. gomelleira	-41,33140182	-12,80720043
F. gomelleira	-75,129058	5,566573
F. gomelleira	-41,326667	-11,493889
F. gomelleira	-73,598689	3,888925
F. gomelleira	-42,893611	-10,110833
F. gomelleira	-40,539444	-12,415278
F. gomelleira	-52,616667	2,233333
F. gomelleira	-42,1391983	-19,78969955
F. gomelleira	-39	-14,1
F. gomelleira	-41,269444	-11,204444
F. gomelleira	-41,709167	-4,099167
F. gomelleira	-42,247222	-13,296389
F. gomelleira	-47,5	-23,95999908
F. gomelleira	-39,083333	-14,416667
F. gomelleira	-39,466667	-4,533333
F. gomelleira	-47,204722	-24,110555
F. gomelleira	-47,29359	-24,01321
F. gomelleira	-40,998056	-11,625556
F. gomelleira	-41,67	-4,107861
F. gomelleira	-45,06999969	-23,43000031
F. gomelleira	-67,084167	-0,098056
F. gomelleira	-41,286944	-10,507778
F. gomelleira	-47,29000092	-22,77000046
F. gomelleira	-42,60969925	-22,70859909
F. gomelleira	-42,2704288	-22,698211
F. gomelleira	-39,133333	-16,333333
F. gomelleira	-50,316944	-6,390833

Species	Longitude	Latitude
F. gomelleira	-48,127222	-24,991944
F. gomelleira	-60,02500153	-3,101939917
F. gomelleira	-42,96749878	-21,45969963
F. gomelleira	-57,4025	-17,851667
F. gomelleira	-40,5821991	-4,141670227
F. gomelleira	-39,419722	-17,099722
F. gomelleira	-52,2	-0,8
F. gomelleira	-43,3375	-11,020278
F. gomelleira	-63,316667	0,816667
F. gomelleira	-41,81140137	-13,57890034
F. gomelleira	-40,351667	-18,846667
F. gomelleira	-42,883333	-22,95
F. gomelleira	-48,50439835	-1,455829978
F. gomelleira	-41,25	-11,916667
F. gomelleira	-45,506944	-23,636111
F. gomelleira	-47,508889	-13,88
F. gomelleira	-49,178333	-26,96
F. gomelleira	-47,834444	-8,438056
F. gomelleira	-48,177222	-13,046111
F. gomelleira	-41,331667	-10,504722
F. gomelleira	-41,5125	-10,170833
F. gomelleira	-41,937222	-10,000556
F. gomelleira	-48,152222	-15,505556
F. gomelleira	-40,210281	-17,120833
F. gomelleira	-43,288333	-13,074167
F. gomelleira	-42,716667	-8,866667
F. gomelleira	-46,670194	-0,926083
F. gomelleira	-41,37080002	-13,00529957
F. gomelleira	-38,96670151	-12,26669979
F. gomelleira	-51,778611	-11,715833
F. gomelleira	-50,018889	-19,226944
F. gomelleira	-49,022222	-26,908056
F. gomelleira	-44,1996994	-20,1432991
F. gomelleira	-43,233333	-22,516667
F. gomelleira	-68,95	-3,484167
F. gomelleira	-69,28	-12,83
F. gomelleira	-43,422472	-19,411222
F. gomelleira	-49,306714	-27,600617
F. gomelleira	-45,101853	-23,074383
F. gomelleira	-44,56330109	-22,49609947
F. gomelleira	-50,635278	-16,985833
F. gomelleira	-43,439167	-18,290278
F. gomelleira	-35,93939972	-8,972499847
F. gomelleira	-47,336139	-1,196139
F. gomelleira	-39,50469971	-13,22859955
F. gomelleira	-46,10559845	-15,91689968
F. gomelleira	-46,989444	-14,278611
F. gomelleira	-46,933611	-14,303889

Species	Longitude	Latitude
F. gomelleira	-59,33530045	-15,22609997
F. gomelleira	-42,439722	-22,928889
F. gomelleira	-42,01860046	-22,87940025
F. gomelleira	-50,55	-12,866667
F. gomelleira	-59	-1
F. gomelleira	-49,27310181	-25,42779922
F. gomelleira	-50,8	-13,3
F. gomelleira	-40,905833	-11,557778
F. gomelleira	-39,999722	-4,071389
F. gomelleira	-47,91999817	-25,01000023
F. gomelleira	-41,465278	-10,624444
F. gomelleira	-41,45	-10,621667
F. gomelleira	-43,79309845	-21,84250069
F. gomelleira	-47,91999817	-24,37999916
F. gomelleira	-43,20920181	-22,11669922
F. gomelleira	-58,622222	-9,036944
F. gomelleira	-57,65330124	-19,00919914
F. gomelleira	-45,40999985	-23,62000084
F. gomelleira	-42,39170074	-22,6508007
F. gomelleira	-48,593333	-25,761111
F. gomelleira	-48,2975	-25,176111
F. gomelleira	-49,078889	-26,905278
F. gomelleira	-52,7	4,083333
F. gomelleira	-74,683333	-12,04065978
F. gomelleira	-67,2	-10,35
F. gomelleira	-62,61666	-14,63333
F. gomelleira	-69,0333333	-11,1833333
F. gomelleira	-67,25	6,28333
F. gomelleira	-76,08333	-0,86666
F. gomelleira	-66,01666	4,05
F. gomelleira	-66,5	-14,75
F. gomelleira	-77,95	-5,86666
F. gomelleira	-66,76666	-10,65
F. gomelleira	-73,25	-3,75
F. gomelleira	-76,85	-1,5333333
F. gomelleira	-77,6	-1,06666
F. gomelleira	-64,51666	-16,58333
F. gomelleira	-76,86666	-0,73333
F. gomelleira	-71,4166667	-11,8333333
F. gomelleira	-72,83333	-3,5
F. gomelleira	-67,98333	-11,73333
F. gomelleira	-62,13166	-16,16916
F. gomelleira	-61,86611	-16,59611
F. gomelleira	-60,8125	-13,92611
F. gomelleira	-52,3178	4,9481
F. gomelleira	-60,9	-13,58333
F. gomelleira	-61,84638	-16,52027
F. gomelleira	-63,16666	7,5

Species	Longitude	Latitude
F. gomelleira	-67,5325	-9,58833
F. gomelleira	-66,744722	-15,419722
F. gomelleira	-61	-13,6
F. gomelleira	-75,05	5,56666
F. gomelleira	-67,79444	-14,6475
F. gomelleira	-67,6530556	-14,6144444
F. gomelleira	-69,11666	-11,16666
F. gomelleira	-79,58333	-0,4
F. gomelleira	-69,6666667	6,8
F. gomelleira	-61,73638	-13,62888
F. gomelleira	-48,4555221	-1,2849922
F. gomelleira	-58,7269878	-2,3065914
F. gomelleira	-52,4926543	-0,8920989
F. gomelleira	-72,40555	-3,69166
F. gomelleira	-41,1030714	-19,3426679
F. gomelleira	-42,29466282	-21,43591886
F. gomelleira	-43,32819372	-22,97431589
F. gomelleira	-39,92070241	-14,6836426
F. gomelleira	-49,92782175	-26,35382218
F. gomelleira	-45,5115278	-23,499444
F. gomelleira	-42,2594756	-19,77441554
F. gomelleira	-48,8099594	-24,233204
F. gomelleira	-43,32869364	-22,94353534
F. gomelleira	-55,5	-21
F. gomelleira	-42,25	-22
F. gomelleira	-47,74583	-13,7975
F. gomelleira	-59,9966667	-2,883333
F. gomelleira	-41,1550667	-9,898083
F. gomelleira	-41,1515083	-9,905194
F. gomelleira	-40,0947278	-9,584264
F. gomelleira	-41,151245	-10,170722
F. gomelleira	-41,1331553	-10,504683
F. gomelleira	-41,1934417	-10,001083
F. gomelleira	-45,5515639	-21,0815
F. gomelleira	-47,75935	-24,0323
F. gomelleira	-59,9470652	-19,15326
F. gomelleira	-60,0651026	-7,230869
F. gomelleira	-55,5782288	-16,82037
F. gomelleira	-61,1944728	-11,195828
F. gomelleira	-41,1570216	-12,553619
F. gomelleira	-42,28742	-20,7528
F. gomelleira	-42,29567	-20,7211
F. gomelleira	-41,13333	-11,15
F. gomelleira	-38,87339	-10,9561
F. gomelleira	-68,82317	-10,9303
F. gomelleira	-67,76167	-10,0667
F. gomelleira	-41,15333	-9,9333
F. gomelleira	-68,86239	-9,4317

Species	Longitude	Latitude
F. gomelleira	-55,5216442	-28,61473
F. gomelleira	-43,32254066	-22,95829671
F. gomelleira	-51,1317981	-8,06
F. gomelleira	-43,3244232	-22,971937
F. gomelleira	-43,32475023	-22,96403694
F. gomelleira	-41,1976795	-21,901462
F. gomelleira	-55,5832205	-15,409056
F. gomelleira	-42,22822161	-22,55363918
F. gomelleira	-40,0010794	-17,982621
F. gomelleira	-43,34604742	-22,98459537
F. gomelleira	-41,10240245	-11,4877814
F. gomelleira	-49,9007992	-25,492994
F. gomelleira	-44,4537458	-22,420659
F. gomelleira	-41,18729634	-20,76547701
F. gomelleira	-42,20423081	-22,42386964
F. gomelleira	-42,2914056	-20,417407
F. gomelleira	-43,39999724	-19,54541172
F. gomelleira	-45,55412846	-20,02073698
F. gomelleira	-44,0766983	-6,601669788
F. gomelleira	-75,61459398	-0,273985642
F. gomelleira	-63,97128121	8,744231211
F. gomelleira	-68,92944	-14,59777
F. gomelleira	-67,02869791	-15,16088721
F. gomelleira	-47,7465842	-13,872463
F. gomelleira	-55,86610031	-1,765560031
F. gomelleira	-39,9583333	-15,166667
F. gomelleira	-67,03036932	3,961174069
F. gomelleira	-64,33333	-12,43333
F. gomelleira	-56,91666	-17,25
F. gomelleira	-53,91666	-10,66666
F. gomelleira	-60,95	9,25
F. gomelleira	-66,5	7
F. gomelleira	-65,88333	10,10194
F. gomelleira	-44,4975583	-12,103361
F. gomelleira	-66,14166	-15,11666
F. gomelleira	-59,96666	-2,88333
F. gomelleira	-46,6413889	-20,258333
F. gomelleira	-64,0688889	-19,3183334
F. gomelleira	-58,8740833	-11,378333
F. gomelleira	-42,2653056	-22,4625
F. gomelleira	-56,62279892	-16,25670052
F. gomelleira	-52,86	2,25
F. gomelleira	-66,50777	-13,59083
F. gomelleira	-46,4233017	-15,61779976
F. gomelleira	-69,13333	-12,56666
F. gomelleira	-65,5275	-9,7888889
F. gomelleira	-73,25444	-12,70222
F. gomelleira	-66,33	-14,85

Species	Longitude	Latitude
F. gomelleira	-48,8217777	-13,167222
F. gomelleira	-47,7217222	-12,906666
F. gomelleira	-70,0730771	-12,56630358
F. gomelleira	-71,6	-11,6833333
F. gomelleira	-39,5833333	-15,1666667
F. gomelleira	-67,6166667	-10,0666667
F. gomelleira	-77,8897222	-1,2430555
F. gomelleira	-69,1691667	-11,8858333
F. gomelleira	-70,25	-12
F. gomelleira	-72,5	-12,5
F. gomelleira	-75	-4
F. gomelleira	-52,28731	-10,8339
F. gomelleira	-41,1234167	-13,397778
F. gomelleira	-42,2243611	-13,296111
F. gomelleira	-39,9516667	-12,933333
F. gomelleira	-39,9083056	-14,793611
F. gomelleira	-37,7543056	-10,482222
F. gomelleira	-46,83056	-14,51944
F. gomelleira	-64,4393611	-9,271944
F. gomelleira	-78,42774778	-5,756237709
F. gomelleira	-67,426389	-16,581667
F. gomelleira	-63,90390015	-8,761940002
F. gomelleira	-64,22720337	-12,44499969
F. gomelleira	-73,425833	4,586111
F. gomelleira	-48,8961944	-26,28
F. gomelleira	-53,24111	3,92472
F. gomelleira	-39,90157674	-9,944686337
F. gomelleira	-42,2308056	-14,343889
F. gomelleira	-42,2037222	-20,704167
F. gomelleira	-56,6818729	-17,32583803
F. gomelleira	-45,5066667	-7,533333
F. gomelleira	-49,91322082	-27,02489805
F. gomelleira	-46,637337	-23,971594
F. gomelleira	-51,1362639	-20,400258
F. gomelleira	-64,4333333	-12,433333
F. gomelleira	-66,95	-15,2
F. gomelleira	-78,42259411	-0,991787905
F. gomelleira	-49,9705556	-24,251111
F. gomelleira	-49,732778	-0,799722
F. gomelleira	-53,28292785	4,821811269
F. gomelleira	-49,26110077	-24,82360077
F. gomelleira	-45,5661583	-23,386901
F. gomelleira	-52,3721157	4,864638452
F. gomelleira	-42,2252536	-11,210025
F. gomelleira	-53,005556	2,383333
F. gomelleira	-47,94191792	-25,1504328
F. gomelleira	-76,2	0
F. gomelleira	-55,568286	4,880095294

Species	Longitude	Latitude
F. gomelleira	-41,1380108	-12,55155
F. gomelleira	-55,5205396	-29,28582
F. gomelleira	-65,87251814	-11,05498941
F. gomelleira	-41,1971996	-21,884795
F. gomelleira	-43,3423591	-22,979614
F. gomelleira	-45,5384444	-23,005278
F. gomelleira	-51,16691256	-29,4502153
F. gomelleira	-63,69551545	-13,27525337
F. gomelleira	-53,04846099	5,064596067
F. gomelleira	-42,2873694	-20,773056
F. gomelleira	-41,1023514	-11,493637
F. gomelleira	-44,4019528	-19,572861
F. gomelleira	-40,0316667	-10,5
F. gomelleira	-40,0433611	-13,415278
F. gomelleira	-41,1250278	-11,376667
F. gomelleira	-42,2833333	-22,433333
F. gomelleira	-49,9748571	-19,284654
F. gomelleira	-41,69072156	-22,29134278
F. gomelleira	-49,9077222	-26,903333
F. gomelleira	-47,7578333	-7,584722
F. gomelleira	-40,0141111	-13,496944
F. gomelleira	-44,4656667	-22,642778
F. gomelleira	-47,7079181	-22,75792749
F. gomelleira	-60,08064964	-7,348055843
F. gomelleira	-44,48	-22,733333
F. gomelleira	-55,5760138	-10,986351
F. gomelleira	-43,3245555	-22,544722
F. gomelleira	-42,2006605	-20,815495
F. gomelleira	-42,2016265	-20,745884
F. gomelleira	-42,2016667	-20,716667
F. gomelleira	-39,95394487	-7,31014254
F. gomelleira	-43,3178091	-22,897195
F. gomelleira	-41,19775	-20,723333
F. gomelleira	-42,2016533	-20,75412
F. gomelleira	-43,39967294	-19,91138785
F. gomelleira	-40,01458047	-18,33746331
F. gomelleira	-39,9657778	-18,103889
F. gomelleira	-39,9664167	-18,069722
F. gomelleira	-39,9878333	-18,364722
F. gomelleira	-43,3044722	-22,975556
F. gomelleira	-53,34324752	4,583718765
F. gomelleira	-47,7787076	-15,86158
F. gomelleira	-59,9465253	-10,162723
F. gomelleira	-39,9338476	-14,846457
F. gomelleira	-49,9281561	-24,782105
F. gomelleira	-58,616667	5,016667
F. gomelleira	-39,9464023	-14,229809
F. gomelleira	-61,1751381	3,751746

**Species**	**Longitude**	**Latitude**
F. gomelleira	-59,947127	-1,919076
F. gomelleira	-61,25733034	10,64147488
F. gomelleira	-67,69532358	-11,01442061
F. gomelleira	-61,733333	8,366667
F. gomelleira	-61,13488222	8,553716539
F. gomelleira	-63,69948459	-17,48071774
F. gomelleira	-76,166667	0
F. gomelleira	-55,32332603	4,504280967
F. gomelleira	-56,6702804	-15,12891
F. gomelleira	-46,6833333	-15,333333
F. gomelleira	-74,14	-9,1839
F. gomelleira	-73	3,5
F. gomelleira	-39,9778056	-18,645556
