# Supplementary material for: Insights into the evolutionary history of the most skilled tool-handling platyrrhini monkey: Sapajus libidinosus from the Serra da Capivara National Park
Source: Genet Mol Biol. 2023 Nov 10;46(3 Suppl 1):e20230165. doi: 10.1590/1678-4685-GMB-2023-0165 (PMC10637428; doi:10.1590/1678-4685-GMB-2023-0165)
Supplement: Table S9 - [file 1415-4757-GMB-46-3-s1-e20230165-s9.pdf]

Genetics and Molecular Biology

https://doi.org/10.1590/1678-4685-GMB-2023-0165

Supplementary Material to "Insights into the evolutionary history of the most skilled tool-handling platyrrhini monkey: S*apajus libidinosus* from the Serra da Capivara National Park"

Table S9 - Occurence data of *Hymenaea martiana* used for the Species Distribution Modeling.

Species	Longitude	Latitude
H. martiana	-35,55388889	-7,603055556
H. martiana	-35,055361	-7,587667
H. martiana	-35,058389	-7,588944
H. martiana	-35,068028	-7,587333
H. martiana	-35,170444	-7,223444
H. martiana	-35,5825	-6,865833
H. martiana	-35,654806	-6,985667
H. martiana	-35,664722	-7,695889
H. martiana	-35,843889	-8,381944
H. martiana	-35,939444	-8,381944
H. martiana	-36,27222	-9,9925
H. martiana	-36,366944	-9,836667
H. martiana	-37,113056	-7,846944
H. martiana	-37,122222	-11,005278
H. martiana	-37,158333	-8,4
H. martiana	-37,17	-8,62
H. martiana	-37,206389	-11,014722
H. martiana	-37,214444	-8,548056
H. martiana	-37,242027	-8,582055
H. martiana	-37,298055	-7,864444
H. martiana	-37,379408	-8,810586
H. martiana	-37,986222	-9,683167
H. martiana	-38,3975	-10,256111
H. martiana	-38,510833	-12,971389
H. martiana	-38,639444	-12,025833
H. martiana	-38,7475	-7,461389
H. martiana	-38,7925	-7,530556
H. martiana	-38,975833	-7,666667
H. martiana	-39,15	-10,032778
H. martiana	-39,15	-10,016667
H. martiana	-39,429166	-8,448611
H. martiana	-39,578056	-8,090833

**Species**	**Longitude**	**Latitude**
H. martiana	-39,579156	-8,889522
H. martiana	-40,35	-10,73333333
H. martiana	-40,294972	-9,184583
H. martiana	-40,301388	-9,187222
H. martiana	-40,333333	-9,35
H. martiana	-40,349416	-9,359555
H. martiana	-40,409736	-9,358336
H. martiana	-40,481277	-9,008166
H. martiana	-40,485924	-11,248653
H. martiana	-40,496886	-9,381733
H. martiana	-40,5	-9,687778
H. martiana	-40,5	-9,4
H. martiana	-40,509167	-11,172778
H. martiana	-40,516667	-9,366667
H. martiana	-40,544694	-9,44125
H. martiana	-40,572097	-9,019383
H. martiana	-40,573883	-9,462777
H. martiana	-40,675556	-9,323611
H. martiana	-40,677725	-9,466394
H. martiana	-40,85	-5,833333
H. martiana	-40,859722	-9,5425
H. martiana	-40,871389	-5,2
H. martiana	-41,31666667	-13,83333333
H. martiana	-41,96666667	-14,86666667
H. martiana	-41,0475	-11,399444
H. martiana	-41,1225	-11,919722
H. martiana	-41,338056	-11,490278
H. martiana	-41,369722	-11,471667
H. martiana	-41,45	-10,621667
H. martiana	-41,502778	-12,826944
H. martiana	-41,631944	-5,310833
H. martiana	-41,795556	-13,597222
H. martiana	-41,815278	-13,603889
H. martiana	-41,83	-13,72
H. martiana	-41,833333	-13,65
H. martiana	-41,835556	-11,753333
H. martiana	-41,966667	-14,883333
H. martiana	-42,52666667	-14,53333333
H. martiana	-42,59305556	-14,77722222
H. martiana	-42	-9
H. martiana	-42,033889	-9,921389
H. martiana	-42,234722	-11,8075
H. martiana	-42,243611	-13,296111
H. martiana	-42,301389	-10,001111
H. martiana	-42,321111	-9,414444
H. martiana	-42,4	-11,4
H. martiana	-42,416667	-13,916667
H. martiana	-42,483333	-14,05

Species	Longitude	Latitude
H. martiana	-42,495278	-14,068889
H. martiana	-42,504167	-10,003333
H. martiana	-42,516667	-14,583333
H. martiana	-42,519048	-14,263365
H. martiana	-42,526667	-14,536111
H. martiana	-42,527778	-11,743056
H. martiana	-42,537778	-14,344167
H. martiana	-42,574167	-14,747222
H. martiana	-42,721111	-11,110833
H. martiana	-42,728333	-9,861944
H. martiana	-42,730833	-10,821944
H. martiana	-42,73092725	-10,82551323
H. martiana	-42,766667	-11,016667
H. martiana	-42,8	-13,633333
H. martiana	-42,816667	-10,783333
H. martiana	-42,817222	-10,793889
H. martiana	-42,819167	-10,785556
H. martiana	-42,833333	-10,8
H. martiana	-42,9	-12,366667
H. martiana	-42,9	-11,866667
H. martiana	-42,906667	-12,329444
H. martiana	-42,991667	-10,907222
H. martiana	-42,995181	-8,727482
H. martiana	-43,15	-18,11611111
H. martiana	-43,32	-12,96666667
H. martiana	-43,041944	-10,959444
H. martiana	-43,1	-5
H. martiana	-43,133333	-11,083333
H. martiana	-43,166667	-11,8
H. martiana	-43,227487	-22,969004
H. martiana	-43,233333	-11,7
H. martiana	-43,293889	-9,84
H. martiana	-43,316667	-12,966667
H. martiana	-43,366417	-10,680167
H. martiana	-43,635556	-18,279444
H. martiana	-43,702847	-13,417311
H. martiana	-43,777778	-14,3275
H. martiana	-43,788889	-14,322222
H. martiana	-43,809444	-14,312778
H. martiana	-44,83	-15,45
H. martiana	-44	-15,01
H. martiana	-44	-15
H. martiana	-44,309722	-13,625833
H. martiana	-44,320556	-15,439722
H. martiana	-44,553056	-16,273056
H. martiana	-44,648889	-14,234444
H. martiana	-44,753611	-15,523056
H. martiana	-44,92	-11,97

Species	Longitude	Latitude
H. martiana	-45,46277778	-18,1
H. martiana	-45,462728	-18,111022
H. martiana	-46,2175	-14,05166667
H. martiana	-46,31666667	-13,66666667
H. martiana	-46,40861111	-14,07972222
H. martiana	-46,41666667	-15,5
H. martiana	-46,5	-12,31666667
H. martiana	-46,5	-14,16666667
H. martiana	-46,87444444	-17,1
H. martiana	-46,98333333	-13,95
H. martiana	-46,333333	-13,666667
H. martiana	-46,408611	-1,406944
H. martiana	-46,416667	-15,5
H. martiana	-46,5	-14,166667
H. martiana	-46,81222	-14,55194
H. martiana	-46,8375	-14,52167
H. martiana	-46,846389	-17,087222
H. martiana	-46,874444	-17,222222
H. martiana	-46,87480632	-17,22595224
H. martiana	-46,980278	-14,277222
H. martiana	-46,991389	-13,952222
H. martiana	-47,33333333	-15,53333333
H. martiana	-47,33333333	-17,33333333
H. martiana	-47,74111111	-7,205277778
H. martiana	-47,92944444	-15,77972222
H. martiana	-47,0825	-13,624444
H. martiana	-47,1	-12,65
H. martiana	-47,233333	-14,116667
H. martiana	-47,3344	-15,5372
H. martiana	-47,462778	-7,3525
H. martiana	-47,47	-6,233333
H. martiana	-47,495556	-7,306944
H. martiana	-47,55	-15,533333
H. martiana	-47,5625	-16,462778
H. martiana	-47,630556	-6,811389
H. martiana	-47,66722	-8,44972
H. martiana	-47,789444	-15,1075
H. martiana	-47,816667	-15,85
H. martiana	-47,874681	-15,766972
H. martiana	-47,92	-15,97
H. martiana	-47,92987267	-15,78342081
H. martiana	-47,933333	-15,733333
H. martiana	-47,936389	-7,864722
H. martiana	-47,940278	-16,774722
H. martiana	-47,9503	-16,2525
H. martiana	-48,24805556	-13,19222222
H. martiana	-48,41666667	-15
H. martiana	-48,52027778	-17,9

Species	Longitude	Latitude
H. martiana	-48	-20
H. martiana	-48,081944	-14,0675
H. martiana	-48,081944	-1,406111
H. martiana	-48,110278	-13,085833
H. martiana	-48,133333	-15,55
H. martiana	-48,171667	-13,483333
H. martiana	-48,17944	-13,38611
H. martiana	-48,19361	-16,33694
H. martiana	-48,213888	-12,761388
H. martiana	-48,217778	-13,167222
H. martiana	-48,256111	-12,756944
H. martiana	-48,283333	-13,93333
H. martiana	-48,283333	-1,393056
H. martiana	-48,33333	-13,83333
H. martiana	-48,380714	-16,255015
H. martiana	-48,387778	-15,646111
H. martiana	-48,4	-16,2875
H. martiana	-48,416667	-15,166667
H. martiana	-48,4775	-15,2675
H. martiana	-48,5	-15,35
H. martiana	-48,5	-17,783333
H. martiana	-48,516667	-14,016667
H. martiana	-48,516667	-14,028333
H. martiana	-48,558889	-18,143333
H. martiana	-48,5678	-20,5572
H. martiana	-48,75	-14,8
H. martiana	-48,75	-17,8
H. martiana	-48,8086	-15,9236
H. martiana	-48,98333	-14,61667
H. martiana	-49,50083333	-15,06277778
H. martiana	-49,166667	-12,2
H. martiana	-49,246389	-20,543611
H. martiana	-49,282222	-14,203889
H. martiana	-49,31	-20,53
H. martiana	-49,3177	-14,2841
H. martiana	-49,504722	-20,321111
H. martiana	-49,516667	-19,916667
H. martiana	-49,530367	-19,922106
H. martiana	-49,853056	-15,933333
H. martiana	-49,966667	-20,7
H. martiana	-50,05796	-20,45739
H. martiana	-50,07418	-20,46994
H. martiana	-50,086667	-20,514444
H. martiana	-50,0927	-17,6803
H. martiana	-50,583333	-10,666667
H. martiana	-51,05361111	-16,91388889
H. martiana	-51,57916667	-16,98916667
H. martiana	-51,82	-17,1

Species	Longitude	Latitude
H. martiana	-51,78	-17,2
H. martiana	-52,133333	-22,916667
H. martiana	-52,8731	-10,8339
H. martiana	-53,08982	-10,77311
H. martiana	-53,214722	-19,037778
H. martiana	-54,589444	-20,52
H. martiana	-54,798889	-20,14
H. martiana	-55,017778	-19,439444
H. martiana	-55,39	-27,6
H. martiana	-55,425278	-20,453333
H. martiana	-56,81888889	-19,17
H. martiana	-56,083333	-20,15
H. martiana	-56,261667	-21,739722
H. martiana	-56,348889	-19,865556
H. martiana	-56,35333	-22,79388
H. martiana	-56,65	-18,983333
H. martiana	-56,7425	-20,781944
H. martiana	-56,83	-19,17
H. martiana	-56,833333	-19,166666
H. martiana	-57,45027778	-19,01833333
H. martiana	-57,25	-25,28333
H. martiana	-57,433611	-18,250277
H. martiana	-57,452778	-19,2
H. martiana	-57,483333	-18,166667
H. martiana	-57,511722	-17,876806
H. martiana	-57,577222	-19,281111
H. martiana	-57,616667	-19,190833
H. martiana	-57,62275	-19,200528
H. martiana	-57,624694	-19,266667
H. martiana	-57,632778	-16,207222
H. martiana	-57,669639	-19,209694
H. martiana	-57,671111	-19,201556
H. martiana	-58,05388889	-15,82694444
H. martiana	-58,1689	-23,2025
H. martiana	-61,43	-15,85
H. martiana	-71,209444	0,072222
H. martiana	-72,91666	-3,25
H. martiana	-74,14	-9,1839
H. martiana	-76,680833	7,666389
H. martiana	-76,73333	8,09805
H. martiana	-77,15917	7,574371
