# Supplementary material for: Insights into the evolutionary history of the most skilled tool-handling platyrrhini monkey: Sapajus libidinosus from the Serra da Capivara National Park
Source: Genet Mol Biol. 2023 Nov 10;46(3 Suppl 1):e20230165. doi: 10.1590/1678-4685-GMB-2023-0165 (PMC10637428; doi:10.1590/1678-4685-GMB-2023-0165)
Supplement: Table S10 - [file 1415-4757-GMB-46-3-s1-e20230165-s10.pdf]

Genetics and Molecular Biology

https://doi.org/10.1590/1678-4685-GMB-2023-0165

Supplementary Material to "Insights into the evolutionary history of the most skilled tool-handling platyrrhini monkey: S*apajus libidinosus* from the Serra da Capivara National Park"

Table S10 - Occurence data of *Manihot dichotoma* used for the Species Distribution Modeling.

Species	Longitude	Latitude
M. dichotoma	-37,85338	-9,97719
M. dichotoma	-40,12119	-13,78182
M. dichotoma	-41,80334	-13,62608
M. dichotoma	-41,83545	-14,28134
M. dichotoma	-35,508056	-6,329722
M. dichotoma	-35,922222	-8,238333
M. dichotoma	-35,944722	-7,376667
M. dichotoma	-36,0442009	-7,903059959
M. dichotoma	-36,436944	-9,250278
M. dichotoma	-36,508056	-6,329722
M. dichotoma	-36,520556	-9,645278
M. dichotoma	-36,624722	-6,331389
M. dichotoma	-36,666667	-8,75
M. dichotoma	-36,75	-6,116667
M. dichotoma	-36,896111	-8,587083
M. dichotoma	-36,90916	-10,27635
M. dichotoma	-36,94273	-9,76788
M. dichotoma	-36,95729	-9,75814
M. dichotoma	-37,1	-5,233333
M. dichotoma	-37,108306	-7,878778
M. dichotoma	-37,13491	-7,04139
M. dichotoma	-37,145028	-9,65975
M. dichotoma	-37,169722	-6,692222
M. dichotoma	-37,2048124	-8,6131782
M. dichotoma	-37,230306	-7,070528
M. dichotoma	-37,237444	-6,976083
M. dichotoma	-37,2459866	-8,5900906
M. dichotoma	-37,2525749	-7,2024906
M. dichotoma	-37,298444	-7,064083
M. dichotoma	-37,303222	-6,980111
M. dichotoma	-37,395833	-5,051944
M. dichotoma	-37,414722	-10,0375

Species	Longitude	Latitude
M. dichotoma	-37,4175	-10,035278
M. dichotoma	-37,4175	-10,046667
M. dichotoma	-37,433333	-8,208056
M. dichotoma	-37,438889	-6,706389
M. dichotoma	-37,451667	-9,749444
M. dichotoma	-37,48621	-9,74073
M. dichotoma	-37,50019	-9,7406
M. dichotoma	-37,55162	-9,14222
M. dichotoma	-37,605028	-8,336111
M. dichotoma	-37,611111	-8,186111
M. dichotoma	-37,684167	-9,805278
M. dichotoma	-37,69029999	-8,540559769
M. dichotoma	-37,731556	-9,641083
M. dichotoma	-37,863778	-9,979444
M. dichotoma	-37,87517	-9,34134
M. dichotoma	-37,98005	-9,55781
M. dichotoma	-38,273611	-9,674722
M. dichotoma	-38,533333	-8,2
M. dichotoma	-38,556944	-10,175556
M. dichotoma	-38,57435	-8,596925
M. dichotoma	-38,645278	-8,091667
M. dichotoma	-38,69	-8,046944
M. dichotoma	-38,708056	-8,156944
M. dichotoma	-38,7225	-9,204444
M. dichotoma	-38,722778	-8,283333
M. dichotoma	-38,740278	-8,1715
M. dichotoma	-38,793389	-8,034917
M. dichotoma	-38,800917	-8,086889
M. dichotoma	-39,1425	-7,975833
M. dichotoma	-39,31000137	-8,514169693
M. dichotoma	-39,329119	-8,386058
M. dichotoma	-39,332222	-9,723056
M. dichotoma	-39,364444	-11,738333
M. dichotoma	-39,55	-10,67
M. dichotoma	-39,6	-10,966667
M. dichotoma	-39,608889	-10,972222
M. dichotoma	-39,633333	-10,95
M. dichotoma	-39,866667	-12,083333
M. dichotoma	-39,866667	-10,666667
M. dichotoma	-40,016666	-13,283333
M. dichotoma	-40,017222	-13,318888
M. dichotoma	-40,12189	-13,79915
M. dichotoma	-40,2	-12,716667
M. dichotoma	-40,212778	-12,729722
M. dichotoma	-40,2347	-15,2392
M. dichotoma	-40,25	-14,2
M. dichotoma	-40,25571	-14,22618
M. dichotoma	-40,266667	-14,133333

Species	Longitude	Latitude
M. dichotoma	-40,283333	-14,116667
M. dichotoma	-40,366667	-14,383333
M. dichotoma	-40,416667	-11
M. dichotoma	-40,50080109	-9,398610115
M. dichotoma	-40,5226632	-14,0314121
M. dichotoma	-40,558361	-9,328944
M. dichotoma	-40,66667	-11,2
M. dichotoma	-40,7772481	-13,1364035
M. dichotoma	-40,818982	-8,847222
M. dichotoma	-40,820556	-8,856389
M. dichotoma	-40,98333	-11,33333
M. dichotoma	-40,999722	-11,676111
M. dichotoma	-41,00500107	-8,515000343
M. dichotoma	-41,03333	-11,38333
M. dichotoma	-41,049483	-13,586025
M. dichotoma	-41,13333	-11,55
M. dichotoma	-41,133333	-14,583333
M. dichotoma	-41,576389	-13,688611
M. dichotoma	-41,66667	-14,18333
M. dichotoma	-41,71293	-4,06688
M. dichotoma	-41,833333	-14,2
M. dichotoma	-41,916667	-13,583333
M. dichotoma	-41,95	-14,2
M. dichotoma	-41,9972	-12,183
M. dichotoma	-42,054722	-11,825833
M. dichotoma	-42,635278	-8,944583
M. dichotoma	-42,636667	-14,0675
M. dichotoma	-42,691917	-9,072231
M. dichotoma	-42,7	-14,8
M. dichotoma	-42,7018	-8,91828
M. dichotoma	-42,75	-14,916667
M. dichotoma	-42,75	-11,083333
M. dichotoma	-42,85989	-15,32616
M. dichotoma	-42,883333	-15,316667
M. dichotoma	-42,916666	-14,91667
M. dichotoma	-43,033333	-15,083333
