# Supplementary material for: Insights into the evolutionary history of the most skilled tool-handling platyrrhini monkey: Sapajus libidinosus from the Serra da Capivara National Park
Source: Genet Mol Biol. 2023 Nov 10;46(3 Suppl 1):e20230165. doi: 10.1590/1678-4685-GMB-2023-0165 (PMC10637428; doi:10.1590/1678-4685-GMB-2023-0165)
Supplement: Table S11 - [file 1415-4757-GMB-46-3-s1-e20230165-s11.pdf]

Genetics and Molecular Biology

https://doi.org/10.1590/1678-4685-GMB-2023-0165

Supplementary Material to "Insights into the evolutionary history of the most skilled tool-handling platyrrhini monkey: S*apajus libidinosus* from the Serra da Capivara National Park"

Table S11 - Occurence data of *Syagrus coronata* used for the Species Distribution Modeling.

Species	Longitude	Latitude
S. coronata	-35,5862	-6,8916
S. coronata	-36,0589867	-8,99658
S. coronata	-36,308111	-9,703133
S. coronata	-36,366944	-9,836667
S. coronata	-36,5078603	-8,948862
S. coronata	-36,5289249	-8,8782449
S. coronata	-36,5812	-10,4716
S. coronata	-36,5945	-10,441001
S. coronata	-36,727778	-8,81
S. coronata	-36,782222	-10,641944
S. coronata	-36,800778	-10,681667
S. coronata	-36,829444	-10,699444
S. coronata	-36,8360682	-10,7146228
S. coronata	-37,0037766	-8,430437
S. coronata	-37,053817	-10,890033
S. coronata	-37,0733	-10,5118
S. coronata	-37,0753	-10,4618
S. coronata	-37,0805	-10,412
S. coronata	-37,1011	-10,4823
S. coronata	-37,1067495	-9,1121528
S. coronata	-37,129611	-10,717167
S. coronata	-37,1353	-10,68851
S. coronata	-37,1651	-10,4732
S. coronata	-37,1944	-11,0429
S. coronata	-37,2113052	-11,1057267
S. coronata	-37,255611	-8,562166
S. coronata	-37,321889	-9,252806
S. coronata	-37,3371872	-10,7671206
S. coronata	-37,338916	-7,965
S. coronata	-37,419861	-10,1035
S. coronata	-37,483889	-10,441667
S. coronata	-37,5125	-11,557778

Species	Longitude	Latitude
S. coronata	-37,566667	-11,5
S. coronata	-37,6	-11,8
S. coronata	-37,616667	-11,816667
S. coronata	-37,65	-10,918056
S. coronata	-37,684167	-9,805278
S. coronata	-38	-12
S. coronata	-38,033333	-12,583333
S. coronata	-38,04169846	-12,26309967
S. coronata	-38,05	-12,016667
S. coronata	-38,083333	-11,933333
S. coronata	-38,11	-10,423056
S. coronata	-38,126389	-9,071944
S. coronata	-38,183333	-10,708333
S. coronata	-38,205556	-11,977222
S. coronata	-38,216431	-9,3830004
S. coronata	-38,25	-9,333333
S. coronata	-38,303611	-12,813611
S. coronata	-38,398333	-10,25
S. coronata	-38,421944	-9,676667
S. coronata	-38,431694	-9,446722
S. coronata	-38,433333	-12,166667
S. coronata	-38,492222	-9,808889
S. coronata	-38,583056	-7,046667
S. coronata	-38,5908	-10,05448
S. coronata	-38,61667	-12,93028
S. coronata	-38,833333	-12,25
S. coronata	-38,875278	-11,711667
S. coronata	-39,046944	-12,221389
S. coronata	-39,051944	-16,370278
S. coronata	-39,059694	-7,466138
S. coronata	-39,068622	-7,463055
S. coronata	-39,073972	-7,458055
S. coronata	-39,083333	-12,533333
S. coronata	-39,142305	-7,501777
S. coronata	-39,2	-14,8
S. coronata	-39,316667	-10,433333
S. coronata	-39,336944	-11,802778
S. coronata	-39,4682942	-12,1906024
S. coronata	-39,532033	-17,9359936
S. coronata	-39,559167	-11,059167
S. coronata	-39,626944	-13,056
S. coronata	-39,7074243	-12,7307325
S. coronata	-39,72065	-12,93843
S. coronata	-39,7351	-12,9499
S. coronata	-39,796389	-12,744167
S. coronata	-39,85	-12,883333
S. coronata	-39,87	-12,72
S. coronata	-39,876289	-12,830086

**Species**	**Longitude**	**Latitude**
S. coronata	-39,887138	-10,636527
S. coronata	-40,028333	-10,731111
S. coronata	-40,03	-11,372083
S. coronata	-40,0808689	-13,5615656
S. coronata	-40,1028245	-14,2944447
S. coronata	-40,123611	-10,388611
S. coronata	-40,135611	-10,231222
S. coronata	-40,140278	-14,039722
S. coronata	-40,156389	-10,379722
S. coronata	-40,166667	-14
S. coronata	-40,1990126	-10,3041935
S. coronata	-40,208503	-16,146383
S. coronata	-40,219167	-12,079722
S. coronata	-40,2206917	-10,2321422
S. coronata	-40,263611	-10,428611
S. coronata	-40,43080139	-13,44110012
S. coronata	-40,475833	-12,335
S. coronata	-40,485861	-11,193472
S. coronata	-40,546389	-11,939722
S. coronata	-40,56676	-13,5114
S. coronata	-40,725833	-11,618611
S. coronata	-40,751556	-13,8875
S. coronata	-40,900444	-8,450444
S. coronata	-40,935611	-8,475055
S. coronata	-41,016667	-11,65
S. coronata	-41,0841854	-13,9204791
S. coronata	-41,153611	-10,232222
S. coronata	-41,236389	-12,902778
S. coronata	-41,266667	-13,8
S. coronata	-41,270278	-11,646944
S. coronata	-41,305	-11,828889
S. coronata	-41,4844017	-17,07419968
S. coronata	-41,48611	-14,6
S. coronata	-41,55889893	-12,52890015
S. coronata	-41,7575	-15,576944
S. coronata	-41,761111	-10,343889
S. coronata	-41,8	-13,3
S. coronata	-41,8216313	-13,6217167
S. coronata	-42,281703	-13,969644
S. coronata	-42,8	-15,4
S. coronata	-42,9125	-22,48222222
S. coronata	-43,704194	-15,30425
S. coronata	-52,8731	-10,8339
S. coronata	-53,08972	-10,77306
