# Supplementary material for: Insights into the evolutionary history of the most skilled tool-handling platyrrhini monkey: Sapajus libidinosus from the Serra da Capivara National Park
Source: Genet Mol Biol. 2023 Nov 10;46(3 Suppl 1):e20230165. doi: 10.1590/1678-4685-GMB-2023-0165 (PMC10637428; doi:10.1590/1678-4685-GMB-2023-0165)
Supplement: Table S12 - [file 1415-4757-GMB-46-3-s1-e20230165-s12.pdf]

Genetics and Molecular Biology

https://doi.org/10.1590/1678-4685-GMB-2023-0165

Supplementary Material to "Insights into the evolutionary history of the most skilled tool-handling platyrrhini monkey: S*apajus libidinosus* from the Serra da Capivara National Park"

Table S12 - Results of the Species Distribution Models for the two studied primates (*S. libidinosus* and *S. nigritus*) and the nine plant species[a].

Species	Nº of records	Feature[b]	Regularization multiplier	AUC test[c]	Parameters	AICc scores[d]	Cross-validate replicates	AUC mean all EV[e]	AUC mean model[f]	Enviromental variables - EV (% of contribution)[g]
Sapajus libidinosus	48	Linear/quadratic	1	0.945	16	1514,843988	5	0.918	0,893	bio_4 (30,2), bio_17 (28,4), bio_12 (2,9), bio_6 (6,8), bio_5 (3,6)
Sapajus nigritus	36	Linear/quadratic	1	0.948	15	1132,381077	5	0.922	0,915	bio_18 (22,1), bio_4 (21,1), bio_6 (17,5), bio_16 (7,4), bio_8 (4,3)
Astrocaryum campestre	70	Linear/quadratic	1	0.946	17	2151,327019	10	0.938	0.914	bio_4 (24,2), bio_17 (28,5), bio_6 (7,8), bio_18 (2,3), bio_19 (7,8)
Syagrus coronata	124	Auto	3	0.967	24	3494,874934	10	0,964	0,965	bio_4 (33,4),

Species	Nº of records	Feature[b]	Regularization multiplier	AUC test[c]	Parameters	AICc scores[d]	Cross-validate replicates	AUC mean all EV[e]	AUC mean model[f]	Enviromental variables - EV (% of contribution)[g]
Manihot dichotoma	116	Auto	3	0.982	24	3241,604811	10	0,981	0,979	bio_2 (14,9), bio_18 (6,0), bio_12 (4,3), bio_8 (2,9)
Anacardium occidentale	956	Quadratic	1	0.836	134	30847,1206	10	0.789	0,708	bio_12 (40,5), bio_7 (32,4), bio_6 (3), bio_14 (2,3)
Attaleas peciosa	67	Linear/quadratic	1	0.885	15	2160,849297	10	0.870	0,866	bio_4 (42,9), bio_6 (14,1), bio_18 (3,2), bio_15 (4,7)
Attalea maripa	117	Auto	3	0.874	22	3772,370483	10	0.853	0,849	bio_11 (30,5), bio_4 (16,8), bio_14 (14,5), bio_18 (11,6), bio_15 (5,5), bio_12 (3,7)
Hymenaea martiana	265	Linear/quadratic	1	0.898	17	8350,65911	10	0,895	0,881	bio_19 (16,1), bio_6 (26,5), bio_16 (8,5), bio_4 (7,2), bio_17 (6,6)
										bio_4 (25,8), bio_19 (22,9), bio_17 (12,4), bio_6 (9,0), bio_2 (5,4), bio_16 (3,5)

Species	Nº of records	Feature[b]	Regularization multiplier	AUC test[c]	Parameters	AICc scores[d]	Cross-validate replicates	AUC mean all EV[e]	AUC mean model[f]	Enviromental variables - EV (% of contribution)[g]
Hymenaea stigonocarpa	877	Auto	1	0.898	103	27282,71045	10	0,895	0,88	bio_17 (22,6), bio_4 (17,3), bio_6 (14,3), bio_12 (14), bio_5 (2,9)
Ficcus gomeleria	477	Auto	1	0.841	97	15443,84253	10	0,821	0,789	bio_7 (44,6), bio_8 (5,9), bio_12 (5,7), bio_9 (3,4)

[a]Number of location records included in the models, results of the statistical tests used to evaluate model discrimination ability (area under the receiver operator curve; AUC) for training and test datasets, and the percent contribution of the different environmental variables.

[b]Feature - The mathematical transformation of the predictor (environmental covariates) created by MaxEnt.

[c]AUC test - The AUC value of parametization test.

[d]AICc– Corrected AIC (akaike information criteria).

[e]AUC mean all EV - The average test AUC for the replicate runs based on all environmental variable (EV).

[f]AUC mean model - The average test AUC for the replicate runs of the models.

[g]bio_2, Mean Diurnal Range (Mean of monthly, max temp - min temp); bio_4, Temperature Seasonality (standard deviation ×100); bio_5, Max Temperature of Warmest Month;bio_6, Min. The Temperature of Coldest Month; bio_7, Temperature Annual Range (bio_5-bio_6); bio_8, Mean Temperature of Wettest Quarter; bio_9, Mean Temperature of Driest Quarter, bio_11, Mean Temperature of Coldest Quarter; bio_12, Annual Precipitation; bio_14, Precipitation of Driest Month; bio_15, Precipitation Seasonality; bio_16, Precipitation of Wettest Quarter; bio_17, Precipitation of Driest Quarter; bio_18, Precipitation of Warmest Quarter; bio_19, Precipitation of Coldest Quarter.
