# Supplementary material for: Insights into the evolutionary history of the most skilled tool-handling platyrrhini monkey: Sapajus libidinosus from the Serra da Capivara National Park
Source: Genet Mol Biol. 2023 Nov 10;46(3 Suppl 1):e20230165. doi: 10.1590/1678-4685-GMB-2023-0165 (PMC10637428; doi:10.1590/1678-4685-GMB-2023-0165)
Supplement: Table S13 - [file 1415-4757-GMB-46-3-s1-e20230165-s13.pdf]

Genetics and Molecular Biology

https://doi.org/10.1590/1678-4685-GMB-2023-0165

Supplementary Material to "Insights into the evolutionary history of the most skilled tool-handling platyrrhini monkey: S*apajus libidinosus* from the Serra da Capivara National Park"

Table S13 - Clades in the Primates phylogeny†

Node	Thousands of years before the present (kya) ‡	95% HPD
Sapajus libidinosus (SCNP) + *Sapajus libidinosus* (UNP)	250	[0,11 - 0,39]
Sapajus libidinosus (SCNP, UNP) + *Sapajus flavius*	420	[0,18 - 0,49]
S. libidinosus (SCNP, UNP) + *S. libidinosus* (TEP) e *S. flavius*	460	[0,24 - 0,55]
S. libidinosus e *S. flavius* + *S. macrocephalus* e *S. apella*	510	[0,30 - 0,63]
S. macrocephalus + *S. apella*	340	[0,18 - 0,51]
S. cay + other *Sapajus*	540	[0,36 - 0,73]
S. robustus + other *Sapajus*	970	[0,66 -1,33]
S. nigritus + other *Sapajus*	2,460	[1,82 - 3,01]
Genus *Sapajus*	3,070	[2,41 - 3,76]
Genera *Cebus* + *Sapajus*	6,360	[5,31 - 7,38]

† Trees showed in the Figure S4. ‡Divergence times and 95% interval of highest probability densities.
