# Supplementary material for: Insights into the evolutionary history of the most skilled tool-handling platyrrhini monkey: Sapajus libidinosus from the Serra da Capivara National Park
Source: Genet Mol Biol. 2023 Nov 10;46(3 Suppl 1):e20230165. doi: 10.1590/1678-4685-GMB-2023-0165 (PMC10637428; doi:10.1590/1678-4685-GMB-2023-0165)
Supplement: Table S14 - [file 1415-4757-GMB-46-3-s1-e20230165-s14.pdf]

Genetics and Molecular Biology

https://doi.org/10.1590/1678-4685-GMB-2023-0165

Supplementary Material to "Insights into the evolutionary history of the most skilled tool-handling platyrrhini monkey: S*apajus libidinosus* from the Serra da Capivara National Park"

Table S14 - The number of individuals *pe*r species or group, nucleotide, and haplotype diversities.

Species	N	Haplotype diversity (H)	Nucleotide diversity (π)
Sapajus libidinosus (SCNP)	47	0.1943+/- 0.0710	0.000181+/- 0.000257
Sapajus libidinosus (UNP)	14	0.7582+/- 0.0841	0.0016584+/- 0.001144
Sapajus libidinosus (TEP)	10	0.6222+/- 0.1383	0.001324+/- 0.000996
Sapajus libidinosus (SNCP) + *Sapajus libidinosus* (UNP)	61	0.5011+/- 0.0698	0.000919+/- 0.000699
Sapajus libidinosus (all samples from Caatinga)	66	0.5604 +/- 0.0626	0.001001 +/- 0.000742

AMOVA analysis. *Sapajus libidinosus* (SCNP) + *Sapajus libidinosus* (UNP): F_{ST} = 0.69390 (*p*-value = 0.00000+-0.00000).
