# Supplementary material for: Insights into the evolutionary history of the most skilled tool-handling platyrrhini monkey: Sapajus libidinosus from the Serra da Capivara National Park
Source: Genet Mol Biol. 2023 Nov 10;46(3 Suppl 1):e20230165. doi: 10.1590/1678-4685-GMB-2023-0165 (PMC10637428; doi:10.1590/1678-4685-GMB-2023-0165)
Supplement: Table S15 - [file 1415-4757-GMB-46-3-s1-e20230165-s15.pdf]

Genetics and Molecular Biology

https://doi.org/10.1590/1678-4685-GMB-2023-0165

Supplementary Material to "Insights into the evolutionary history of the most skilled tool-handling platyrrhini monkey: S*apajus libidinosus* from the Serra da Capivara National Park"

Table S15 - Demographic parameters and effective size for each software.

**Software**	**θ**	***g***	**Median N_e (95% HPD[†] or CI[‡])**
BEAST (BSP)	-	-	21,791 (1,709 – 226.334)
LAMARC (without *g*)	0.001746 (0.000694 – 0.003642)	-	14,550 (5,783 – 30,350)
LAMARC (with *g*)	0.001921 (0.000718 – 0.004371)	912.7075 (-431.4512 – 979.0675)	16,008 (5,983 – 36,425)
DIYABC (Best scenario) N_e (current effective size)	-	-	21,800 (11,800 – 29,400)
DIYABC (Best scenario) N_a (ancestral effective size)	-	-	12,900 (6,320 – 24,100)

Note: BSP stands for Bayesian Skyline Plot. Theta (θ) value used was without *g* factor due to low reliability in the parameter. [†]HPD is the 95% High Posterior Density. [‡]CI is the 95% Credibility intervals.
