# Supplementary material for: Insights into the evolutionary history of the most skilled tool-handling platyrrhini monkey: Sapajus libidinosus from the Serra da Capivara National Park
Source: Genet Mol Biol. 2023 Nov 10;46(3 Suppl 1):e20230165. doi: 10.1590/1678-4685-GMB-2023-0165 (PMC10637428; doi:10.1590/1678-4685-GMB-2023-0165)
Supplement: Figure S2 - [file 1415-4757-GMB-46-3-s1-e20230165-s17.pdf]

Genetics and Molecular Biology

https://doi.org/10.1590/1678-4685-GMB-2023-0165

Supplementary Material to "Insights into the evolutionary history of the most skilled tool-handling platyrrhini monkey: S*apajus libidinosus* from the Serra da Capivara National Park"

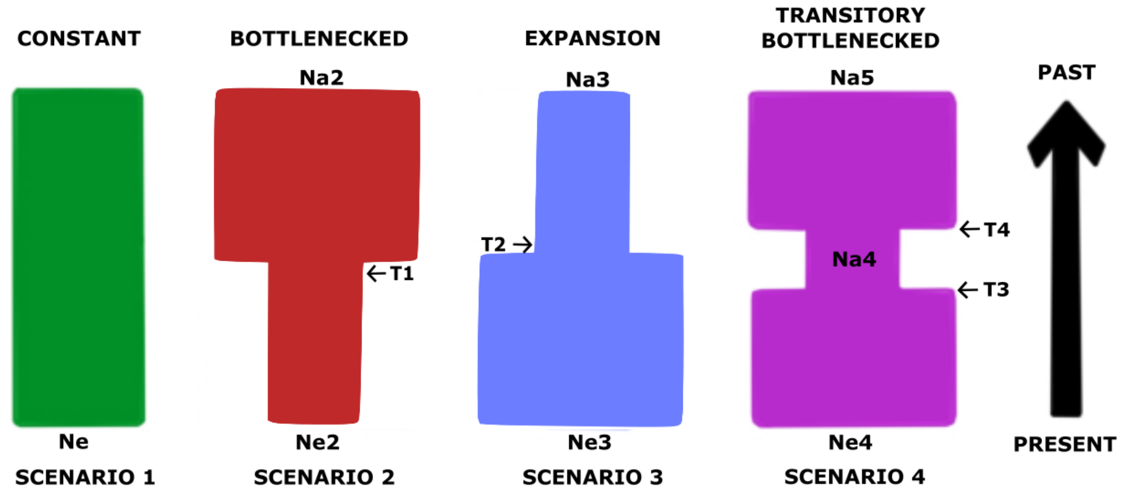

Figure S2 - Historical scenarios analyzed on DIYABC 2.1. Scenario 1 is the null hypothesis that assumes a population whose effective size (N_e) remained constant over time. Scenario 2 assumes an ancestral effective population size (N_a2) that has declined to its current effective size (N_e2) in T1 generations. Scenario 3 assumed an ancestral effective population size (N_a3) that increased to its current effective size (N_e3) in T2 generations ago. For Scenario 4, we consider two times (T3 and T4) between periods that we place as a general interval in which possible significant events could have occurred, with a momentary decline in the effective population size (N_a4). The four scenarios were compared for both "BSP model" and "LAMARC model", amounting eight different scenarios.
