# Supplementary material for: Insights into the evolutionary history of the most skilled tool-handling platyrrhini monkey: Sapajus libidinosus from the Serra da Capivara National Park
Source: Genet Mol Biol. 2023 Nov 10;46(3 Suppl 1):e20230165. doi: 10.1590/1678-4685-GMB-2023-0165 (PMC10637428; doi:10.1590/1678-4685-GMB-2023-0165)
Supplement: Figure S3 - [file 1415-4757-GMB-46-3-s1-e20230165-s18.pdf]

Genetics and Molecular Biology

https://doi.org/10.1590/1678-4685-GMB-2023-0165

Supplementary Material to "Insights into the evolutionary history of the most skilled tool-handling platyrrhini monkey: S*apajus libidinosus* from the Serra da Capivara National Park"

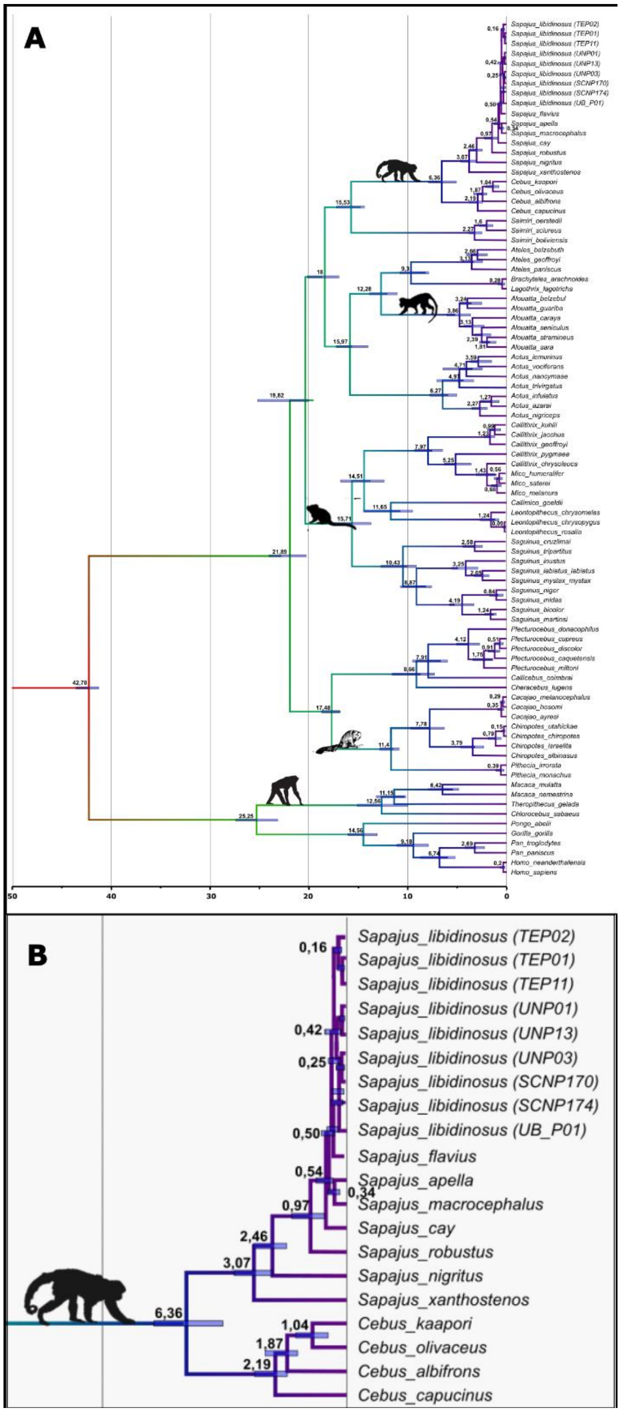

Figure S3 - (A) Phylogenetic tree generated in BEAST, based on *CYTB* sequences. One individual per species, except for *S. libidinosus* sampled in the Pedra Furada (PF), site located at the Serra da Capivara National Park (SCNP), Ubajara National Park (UNP), and Tietê Ecological Park (TEP). In these three cases, the different haplotypes found in the present study were considered. (B) Detail of the phylogenetic tree considering the genera *Sapajus* and *Cebus* species. This Figure shows that the divergence between the genera *Cebus* and *Sapajus* was estimated at approximately 6.36 million years ago (mya) [Highest Posterior Density (HPD) Interval = 5.31 – 7.38] (Table S13). This result strengths previous estimated mean values in the literature using mitochondrial, nuclear sequences and/or SNPs (~5.7-~6.8 mya) (Perelman et al., 2011; Lynch-Alfaro et al., 2012, 2015; Lima et al., 2017; Martins-Junior et al., 2018; Martins et al., 2023). The separation between *S. libidinosus* and *S. flavius* was estimated at 420 kya (HPD = 0.18-0.49), a number similar to that found by Martins et al. (2023).
