# Supplementary material for: Insights into the evolutionary history of the most skilled tool-handling platyrrhini monkey: Sapajus libidinosus from the Serra da Capivara National Park
Source: Genet Mol Biol. 2023 Nov 10;46(3 Suppl 1):e20230165. doi: 10.1590/1678-4685-GMB-2023-0165 (PMC10637428; doi:10.1590/1678-4685-GMB-2023-0165)
Supplement: Figure S4 - [file 1415-4757-GMB-46-3-s1-e20230165-s19.pdf]

Genetics and Molecular Biology

https://doi.org/10.1590/1678-4685-GMB-2023-0165

Supplementary Material to "Insights into the evolutionary history of the most skilled tool-handling platyrrhini monkey: S*apajus libidinosus* from the Serra da Capivara National Park"

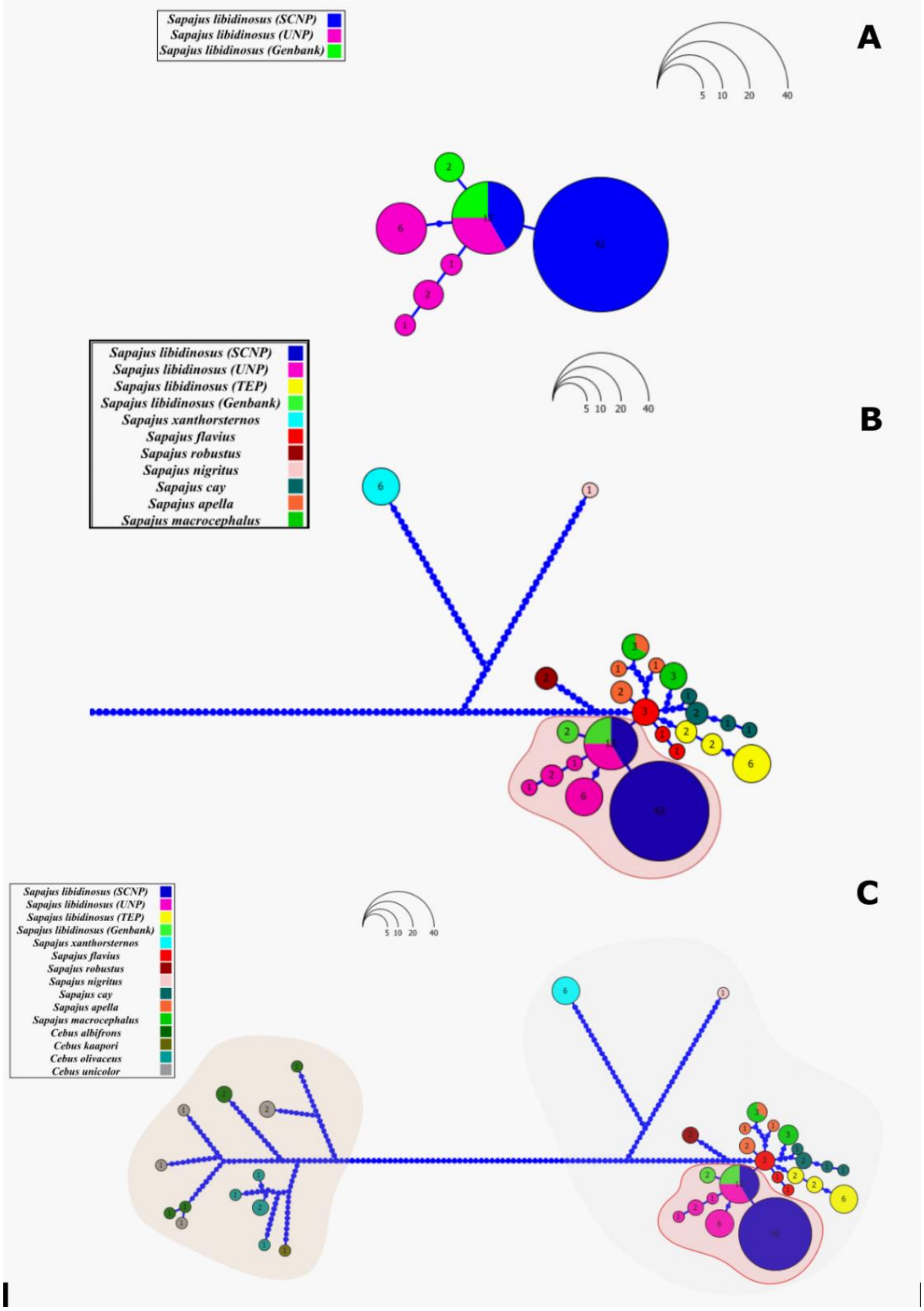

Figure S4 - Haplotype network based on *CYTB* sequences. Haplotype network of *Sapajus* species and *Cebus* species as outgroup. The size of each haplotype circle is proportional to the number of individuals observed in it. Each dot on a line connecting two haplotypes indicates a mutational step. The colors correspond to the different species described in the legend. The overlapping shapes indicate the groups, the red indicates the species *S. libidinosus* from the Caatinga, and the gray colors indicate the different genera (*Cebus* and *Sapajus*). The intersection of lines can indicate a mean vector interpreted as an unsampled but possibly existing sequence (haplotype) or even extinct ancestral sequences.
