# Supplementary material for: Insights into the evolutionary history of the most skilled tool-handling platyrrhini monkey: Sapajus libidinosus from the Serra da Capivara National Park
Source: Genet Mol Biol. 2023 Nov 10;46(3 Suppl 1):e20230165. doi: 10.1590/1678-4685-GMB-2023-0165 (PMC10637428; doi:10.1590/1678-4685-GMB-2023-0165)
Supplement: Figure S5 - [file 1415-4757-GMB-46-3-s1-e20230165-s20.pdf]

Genetics and Molecular Biology

https://doi.org/10.1590/1678-4685-GMB-2023-0165

Supplementary Material to "Insights into the evolutionary history of the most skilled tool-handling platyrrhini monkey: *Sapajus libidinosus* from the Serra da Capivara National Park"

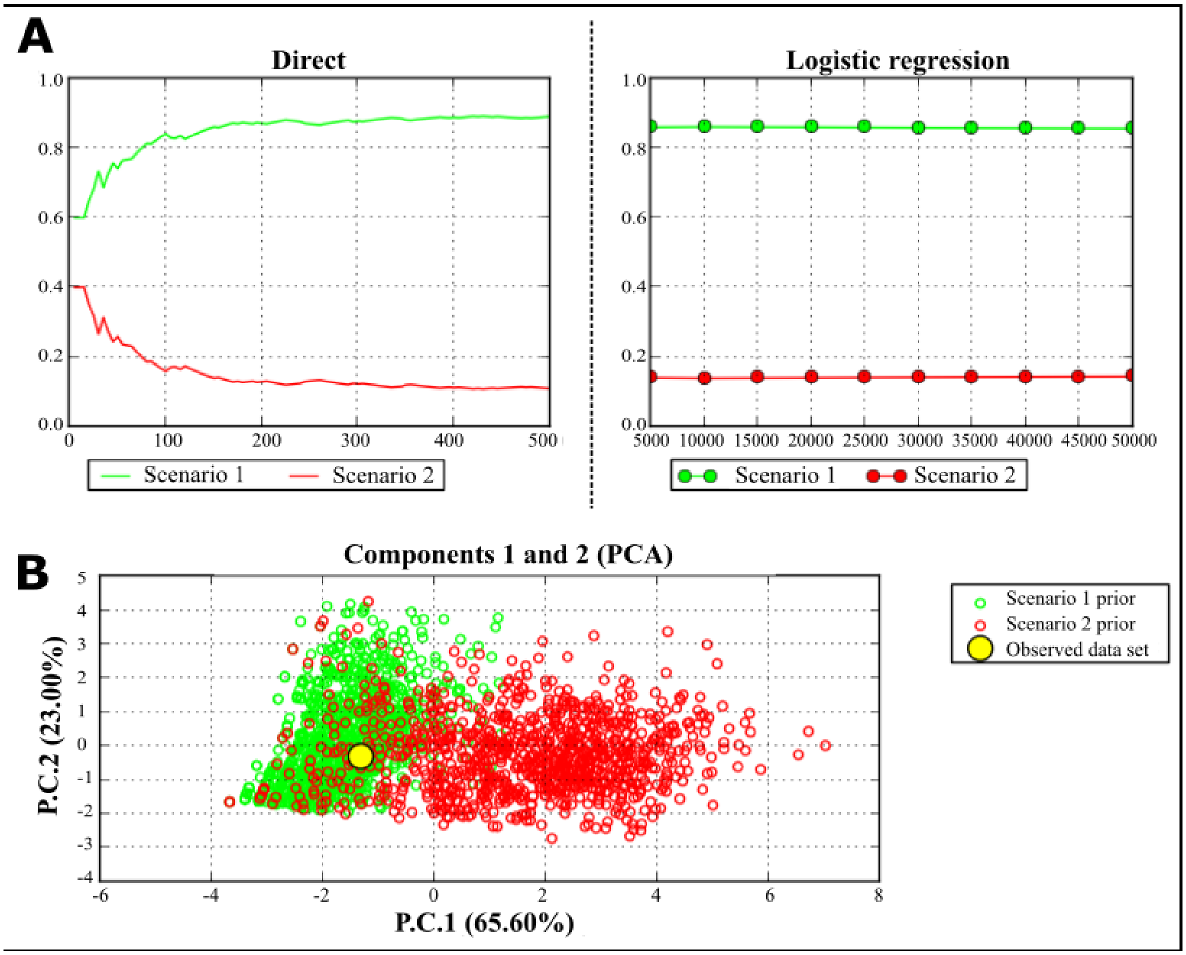

Figure S5 - Comparison of the two best scenarios of each methodology, Bayesian Skyline Plot (BSP) and LAMARC, both selecting population expansion. (A) Estimates of the posterior probability of each scenario and their comparison. Direct estimate: The number of times a given scenario is closest to the simulated datasets. Logistic regression: A logistic regression based on the first closest datasets with the proportion contributed by each scenario and each running point. In all these cases, Scenario 1 (LAMARC) better explained the observed data. (B) Principal component analysis: visual information about how close the simulated datasets in each scenario are to the observed dataset. In this analysis, scenario one also fits better with the observed data (Cornuet et al.,2008).
