# Supplementary material for: Insights into the evolutionary history of the most skilled tool-handling platyrrhini monkey: Sapajus libidinosus from the Serra da Capivara National Park
Source: Genet Mol Biol. 2023 Nov 10;46(3 Suppl 1):e20230165. doi: 10.1590/1678-4685-GMB-2023-0165 (PMC10637428; doi:10.1590/1678-4685-GMB-2023-0165)
Supplement: Figure S7 - [file 1415-4757-GMB-46-3-s1-e20230165-s22.pdf]

Genetics and Molecular Biology

https://doi.org/10.1590/1678-4685-GMB-2023-0165

Supplementary Material to “Insights into the evolutionary history of the most skilled tool-handling platyrrhini monkey: S*apajus libidinosus* from the Serra da Capivara National Park”

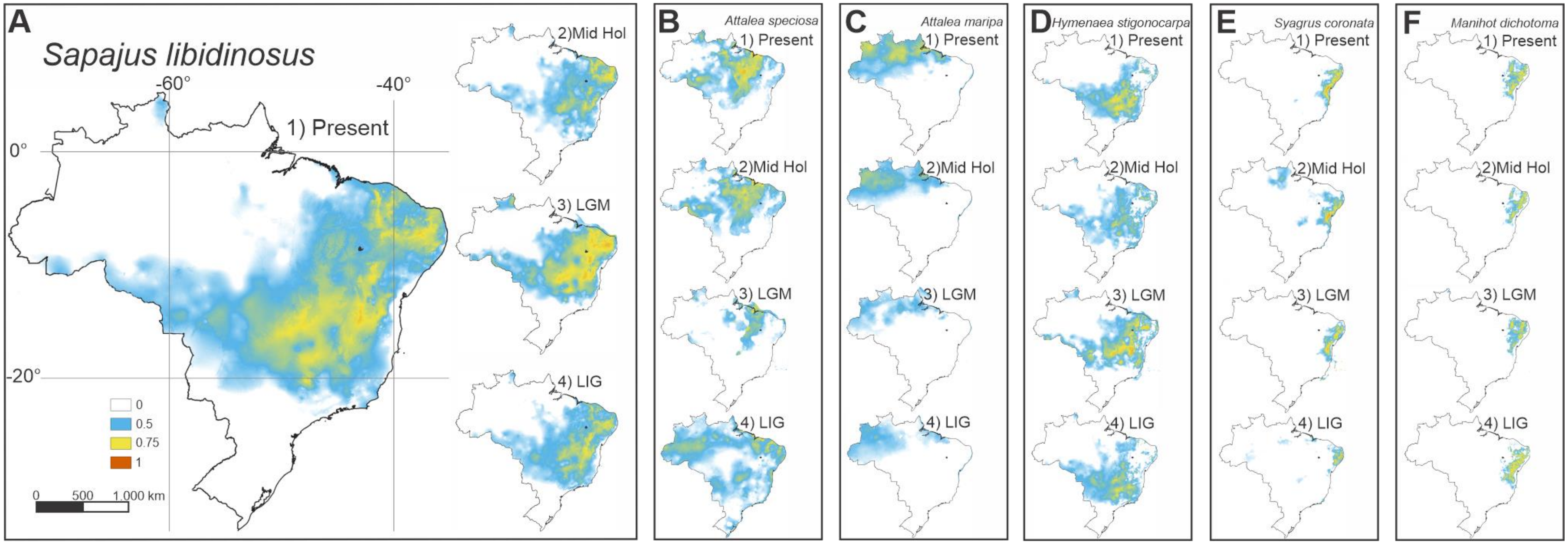

Figure S7 - Species Distribution Model (SDM) results for *Sapajus libidinosus, Attalea speciosa, Attalea maripa, Hymenaea stignocarpa, Syagrus coronate,* and *Manihot dichotoma.* Predictive distributions were inferred based on (1) Present, (2) Middle Holocene (~6 kya), (3) Last Glacial Maximum – LGM (~22 kya), and (4) Last Interglacial – LIG (~140-120 kya) conditions. White indicates areas of unsuitable habitat, with green, yellow, and red indicating increasing suitability of habitat for species. The Serra da Capivara National Park, located in the state of Piauí (Figure S1), appears marked on the maps.
