# Supplementary material for: Insights into the evolutionary history of the most skilled tool-handling platyrrhini monkey: Sapajus libidinosus from the Serra da Capivara National Park
Source: Genet Mol Biol. 2023 Nov 10;46(3 Suppl 1):e20230165. doi: 10.1590/1678-4685-GMB-2023-0165 (PMC10637428; doi:10.1590/1678-4685-GMB-2023-0165)
Supplement: Supplementary material (Materials and Methods) - [file 1415-4757-GMB-46-3-s1-e20230165-s23.pdf]

Genetics and Molecular Biology

https://doi.org/10.1590/1678-4685-GMB-2023-0165

Supplementary Material to "Insights into the evolutionary history of the most skilled tool-handling platyrrhini monkey: S*apajus libidinosus* from the Serra da Capivara National Park"

Material and Methods

Samples, DNA extraction, sequencing, and ethical authorization

The Serra da Capivara National Park (SCNP; 8.8333°S, 42.5500°W; Figure S1) has 91,848.88 hectares (918.5 km^2) and was created in 1979 to protect the rich pre-Colombian archaeological heritage of the region. Due to its historical and cultural value, the SCNP was declared a World Heritage Site by UNESCO in 1991 (https://www.icmbio.gov.br/portal/visitacao1/unidades-abertas-a-visitacao/199-parque-nacional-da-serra-da-capivara). SCNP is located within the Caatinga biome in the northeastern state of Piauí, northeastern Brazil (Figure S1). SCNP is home to numerous capuchin groups, but the population investigated here is known as the PF group. They have been studied since 2007 (Falótico and Ottoni, 2013), and the research team has identified adults and other younger group members. This population is well known for having the most diverse tool use repertoire among capuchin monkeys (*Sapajus* and *Cebus*), using stone and wood tools for several actions (Falótico and Ottoni, 2013; 2014; 2016). Sixty-seven samples were collected from at least 47 known individuals (duplicate samples were collected). The composition of the group fluctuated in the sampled period from 38 to 47 individuals. Good quality DNA was extracted from the fecal material of these *S. libidinosus* individuals. It is important to comment that *Sapajus libidinosus* is a species classified as "Near Threatened" by the Red List of the International Union for Conservation of Nature (IUCN) (Martins et al., 2021) and is at risk of losing 54% of its habitat in the Caatinga in the next 50 years (Moraes et al., 2020). The *S. libidinosus* groups in SCNP are particularly vulnerable, as a decline in population may lead to the end of their tool-using culture (Presotto et al., 2020).

The Ubajara National Park (UNP; 3.833°S, 40.896°W) has 563 ha (5.63km^2) and was created in 1959. UNP is located within the Caatinga biome in the state of Ceará, northeastern Brazil. UNP has several groups of capuchins (*S. libidinosus*), that customarily use stone tools to

process encased fruits (Falótico et al., 2023). The population sampled here is called the “Sertão group” and has been investigated since 2021. This group had a range of 28-30 individuals. Fifteen samples from UNP were collected opportunistically in two periods (Oct/2021 and Nov/2021) using the same protocol above. Most of the samples have individual identities (6 individuals), but five of them have only the information of age class, and sex. Fourteen individuals had their *CYTB* sequenced to present study.

The Serra da Itabaiana National Park (SINP; 10.812°S, 37.291°W) has 8,000 ha ($80km^2$), and is in the state of Sergipe, Brazil. SINP is located in the Atlantic Forest biome but presents a transition to Caatinga in the north area. It is home to several yellow-breasted capuchin monkeys (*Sapajus xanthosternos*) groups. As far as we know, the monkeys in this population do not use stone tools, although the phenomenon has already been described in other populations (Canale et al., 2009). We have mapped a location in the south of the park, where at least one group of around 20-25 monkeys live (we have observed them during the mapping) and found no sign of stone tools processing sites (Falótico et al. in preparation) Eight samples from SINP were collected on March 18th, 2022, below a tree where the monkeys had been foraging 20min before. This capuchin group is not habituated, so it was not possible to have detailed information about the individuals that were sampled. Six individuals had their *CYTB* sequenced to present study.

One of us (Tiago Falótico; TF) used a minimally invasive sample collection from an SCNP/PF, UNP and SINP populations. Initially, a non-invasive strategy was developed with traps to collect hair, but it did not work, as the animals (this strategy was tried in the SCNP/PF) soon learned how to obtain the fruit inside the trap without the loss of any hair on the adhesive tapes at the opening structure of the trap. Subsequently, fecal samples were collected in the fieldwork during the period in which PF members were followed to collect behavioral data. The fieldwork seasons were Jun-Jul/2015, Dec/2017, May/2019, and Oct/2019. The collections in the other locations (UNP and SINP) took place after the local conditions of the Covid-19 pandemic allowed. When an individual was observed to evacuate, the researcher waited for the monkey to leave the location and then approached the place and searched carefully for the feces. It was collected if the feces were located and isolated for potential contaminants (*e.g.,* feces of other animals, urine, and food). The researcher collecting the sample used masks to avoid any contamination, and a sample (the whole feces, if possible) was pushed inside a sterile sample tube (Sarstedt tube 76x20) using a sterile spoon attached to the cap of the tube. The spoon was then detached and discarded. Since the individuals in the SCNP/PF have been investigated for years, the identification of each individual, with their proper names, was provided to the tubes with the biological samples. After that, the tube was stored in a thermic bag with ice, and at the end of the day, it was moved to a freezer until DNA extraction.

Tietê Ecological Park, located in the eastern zone of São Paulo city, was inaugurated in 1982. Additionally, for comparative purposes, ten individuals identified as *S.libidinosus* from the Tietê Ecological Park (TEP) (https://www.saopaulo.sp.gov.br/conhecasp/parques-e-reservas-naturais/parque-ecologico-do-tiete/) were also obtained. The founding individuals (two males and three females) were released on the TEP after confiscation by the Brazilian Institute for the Environment and Renewable Natural Resources (IBAMA). The origin of the founder specimens was unknown. Although the group is supplied daily, these Capuchin monkeys scan the entire area, consuming naturally available items. They use stone tools to open hard-shelled fruits (Ottoni and Mannu, 2001; Coelho et al., 2015; Izar et al., 2016). Regarding these TEP specimens, there is a caveat that some of them may be hybrids between *S. libidinosus* and *S. nigritus* or *S. apella*, according to observations based on visible phenotypes such as coat color (Ferreira, 2004). The animals are provisioned daily, but they also forage for naturally available resources and the mature individuals crack nuts, although with variable frequency and efficiency (Ottoni and Izar, 2008).

DNA samples from *S. libidinosus* were extracted using the Qiagen Stool Kit® and Power Fecal Kit® according to the manufacturer's protocols. For the other specimens of interest (see below), DNA was extracted from blood and/or tissue using the Qiagen DNeasy Blood and Tissue Kit®, according to the manufacturer's instructions. The visualization observed the success of the extraction in UV transilluminator of the DNA on 1% agarose gel with the addition of DNA dye (GelRedTM). The DNA samples were quantified using a spectrophotometer (NanoDrop® - Uniscience). A low-mass molecular marker (100 bp) was used as a control during electrophoresis.

In addition, we also used five Caatinga *S. libidinosus* sequences from Genbank (Lima et al., 2017) and for comparative purposes, other Platyrrhini species were also investigated: sequence data for *CYTB* of the species *Callicebus coimbrai, Mico melanura, Mico saterei, Saguinus martinsi*, and *Saguinus niger* were obtained for the present study (one individual per species). To date, the variability of this gene in these species is unknown. We also generated original *CYTB* data for the species *Chiropotes utahickae* and *Mico humeralifer* and used them in our analyses because the sequences available in public banks are incomplete. The original samples were provided by the Rio de Janeiro Primatology Center (CPRJ). This center is geographically located in Rio de Janeiro State (Brazil), between 22º27'S-22º32'S and 42º50'W-42º56'W, in an area of 239.54 hectares (~2.4 km²) with 95% forest cover, where the animals are kept in captivity, without public access (http://mapadecultura.rj.gov.br/guapimirim/centro-de-primatologia-do-rio-dejaneiro/).

We selected *CYTB* for sequencing because it has been considered the primary genetic locus in the classification and characterization of species (Bradley and Baker, 2001; Cao and

Wu, 2019; Kumar et al., 2017), at least so far, as genomic data for these species are still relatively scarce. For instance, a study of the DNA sequences of 217 mammalian species revealed that *CYTB* more accurately reconstructs the phylogeny and known relationships between species based on other molecular and morphological analyses at superorder, order, family, and generic levels (Tobe et al., 2010). More recently, Schrago and Mello (2020) demonstrated that *CYTB* genetic distances remains a valuable tool for the broad characterization of mammalian within- and between-species divergences, including primates, indicating that it can also be useful for population studies.

We used for the amplification of the *CYTB* gene the primers and conditions according to Irwin et al. (1991) and Anderson and Yates (2000). As a complement to the *CYTB* original sequences generated for the present study, data from other Primate species were obtained from public banks (Table S1).

Gene sequences were obtained through an external service provider (Macrogen; https://dna.macrogen.com). The sequences were verified and aligned by us using the MUSCLE algorithm, as implemented in the AliView v.1.26 software (Larsson, 2014)

The present project is registered in three official Brazilian systems: the Biodiversity Authorization and Information System (SISBIO; protocol number 57039; 09/01/2017), which permits the collection of biological material from conservation units for research; and SISGEN (National System for the Management of Genetic Heritage and Associated Traditional Knowledge; protocol number AF00ED5; 27/09/2018), an instrument to assist the Genetic Heritage Management Council (CGEN) in the management of genetic heritage and associated traditional knowledge.

Field activities in the SCNP, including the collection of biological samples, were authorized by IBAMA/ICMBio 60134, and registered in the SISBIO (protocol numbers: 48323-1, 05/05/2015 and 59019-1, 23/06/2017). The State Environmental Institute (INEA), linked to the State Department of the Environment of “Rio de Janeiro” also authorized the genetic studies using CPRJ specimens (administrative procedure E-07 / 002.12978 / 2017). The Animal Ethics Committee of the “Universidade Federal do Rio Grande do Sul” approved the project “The molecular evolution of behavioral genes in primates” on November 25, 2014. The Animal Ethics Committee of the “Universidade de São Paulo” also approved the project (CEUA/IP/USP#3036140715).

Our studies with these samples comply with the principles proposed by the American Society of Primatologists for the ethical treatment of non-human primates (https://www.asp.org/society/resolutions/Ethical TreatmentOfNonHumanPrimates.cfm)

Diversity indices and network analysis

The diversity indices (nucleotide diverstity [$\boldsymbol{\pi}$] and haplotype diversity [H]) and genetic structure analysis (F_{ST}) were calculated using the *S. libidinosus CYTB* gene dataset, using Arlequin v.3.5.2.2 (Excoffier and Lischer, 2010). The DnaSP v.6 program (Rozas et al., 2017) was used to identify the haplotypes of the *S. libidinosus* CYTB sequences and generate data matrices. The hierarchical relationships between haplotypes were observed from haplotype networks generated by the Haplotype Viewer program (Salzburger et al., 2011), which is based on the implementation of the Phylip package algorithm (phylogeny inference package) that generates a tree from of the maximum likelihood method (DNAML) (Felsenstein, 1993).

Demographic history

A Bayesian skyline plot (BSP, Drummond et al., 2005) was generated using the BEAST v.2.6.7 program (Bouckaert et al., 2019) to identify possible changes in the effective population size (N_e; weighted for males and females) over time, based on the CYTB data set of S. libidinosus. This approach incorporates uncertainty in the genealogy using the integration of the Markov chain Monte Carlo (MCMC) under a coalescent model, in which the time of dates provides information on effective population sizes over time, allowing us to estimate demographic fluctuation over time. The chains were executed for 200 million iterations, from a random starting tree, and sampled every 5,000 generations for the Caatinga biome (including samples from SCNP, UNP, and Genbank sequences) dataset. The first 10% was discarded as burn-in. We tested BSP with the substitution model GTR+I+G selected according to the results of the jModelTest v.2.1.9 program (Darriba et al., 2012) and present in BEAST 2 package, under the Akaike Information Criterion (AIC), and the BEAST model in which the very software estimates the best-fitting substitution model. We used a strict clock and a rate of $2x10^{-8}$ mutations per site per year (Brown et. al., 1979). The demographic history over time was reconstructed using Tracer v. 1.7 (Rambaut et al., 2018) and Effective Sample Sizes (ESSs) > 200 were checked in the same software.

Another demographic analysis was performed using LAMARC 2.1.10 (Kuhner, 2006) We estimated the molecular diversity parameter theta (Θ, Watterson, 1975) and growth rate (*g*) to estimate N_e and population patterns (expansion, stability, or contraction) Through the Θ parameter we can estimate N_e using the formula $\Theta = N_e \times \mu$ (according to the software developers for mtDNA), where μ is the mutation rate in generations. We used the same molecular clock adjusted for generations (i.e 1.2×10^{-7}, considering a generation time of 6 years for *S. libidinosus* (Gage, 1998) We used a Bayesian search strategy with three replicas, each run for 15,000,000 iterations, sampling every 100 generations, with a 10% burn-in. The best substitution model used was GTR estimated in jModelTest v.2.1.9 (Darriba et al., 2012) from

the available models present in the LAMARC software. All ESS values (> 200) were also checked using Tracer v. 1.7 (Rambaut et al. 2018).

Finally, Approximate Bayesian Computation (ABC; Beaumont, 2010) analyzes were conducted to compare scenarios of population stability, bottleneck, expansion, and transitory bottleneck using the BSP N_e estimates and LAMARC N_e estimates using DIYABC v. 2.1.0 (Cornuet et al., 2014), amounting eight different historical scenarios (Figure S2). This approach allows the choice of the demographic scenario that best-fits the related data to the value of posterior probabilities of the parameters. The posterior probability of each scenario was estimated using logistic regression and direct method (Cornuet et al. 2008). For the best scenario we estimated the analysis of model verification which compares the simulations between prior and posterior distributions and a "real" dataset considering various parameter sets.

We conducted 8,000,000 simulations as indicated by the software and because the GTR model (used in LAMARC runs) is not implemented in the DIYABC software, we used TN93+I+G as the substitution model and the same mutation rate used in BSP and LAMARC. We also conducted a comparison of the best scenario of each model (Figure S2) with 5,000,000 simulations and used the median values of current N_e (*Ne*), ancestral N_e (*Na*), and generation time (T) to calculate population patterns (whether growth, stability, or contraction).

Parameters observed for scenario choice were (i) pre-evaluation of scenario-prior combinations using Principal Component Analysis (PCA), locating the observed dataset among the simulated dataset, which allows for checking if a chosen set of prior distributions and models (scenarios) can generate a collection of summary statistics that closely match the observed summary statistics, thereby verifying their potential; (ii) computation of posterior probabilities of scenarios, which involves estimating the (relative) posterior probabilities of a finite number of models through two approaches - direct and logistic regression-based estimations; and (iii) evaluating the confidence in scenario choice, in which simulated pseudo-observed data sets (pods) are generated by drawing parameter values from prior distributions using fixed values. The ABC posterior probabilities are then computed from these pods to evaluate false allocation rates (type I and II errors) and determine confidence in model choice empirically. For the best scenarios more analyzes were performed including (iv) estimation of posterior distribution of parameters, which computes the Euclidean distances between the simulated and observed datasets in the space of summary statistics. The simulates datasets that are closest to the observed datasets are kept, and the parameter values used to simulate these selected datasets are then used to provide a sample of parameter values that are approximately distributed based on their own posterior distribution; (v) computation of bias and precision on parameter estimations, in which the comparison of the real values to the estimated values of

parameters can give insights into the precision of the estimation process; and (vi) a model checking using PCA, which evaluates the dissimilarity between a model and parameter posterior distributions and a real dataset, by analyzing numerous sets of test variables (see Cornuet et al., 2014 - Supplementary data).

For the current Caatinga N_e we used both values obtained in the BSP (wider interval) and LAMARC analyzes (stricter interval). For ancestral Caatinga N_e (Na) we used an uniformative uniform distribution between 1 and 1,000 generations (which was the approximated time interval captured in the BSP analysis). However, preliminary runs were done using shorter and larger intervals (6 to 6,000 years, 6,000 to 12,000 years, 12,000 to 22,000 years, 22,000 to 40,000 years, 40,000 to 120,000 years, 120,000 to 200,000 years, and a more general one from 6 to 200,000 years). The summary statistics (SuSt) chosen were the following: number of haplotypes, number of segregating sites, mean pairwise differences, variance of pairwise differences, private segregation sites, mean of number of the rarest nucleotide at segregating sites, and the variance of numbers of the rarest nucleotide at segregating sites, because these were the SuSt that retained the highest percentage of total information in preliminary runs. For all Bayesian analyzes and calculations we used median values and 95% HPD or 95% Credibility Intervals (CI) depending on the software and their methodologies.

Times of divergence

The BEAST v.2.6.7 package (Bouckaert et al., 2019) was used to infer the phylogeny and divergence times based on data from the *CYTB* gene of Platyrrhnini primates (Table S1). We performed the Markov Chain Monte Carlo (MCMC) analysis for 100 million iterations and sampled states every 5,000 generations with a random starting tree and relaxed molecular clock log normal, and default distributions considering other parameters. We employed the Yule Method as the prior tree. The substitution model was GTR+I+G, selected according to the program jModelTest v.2.1.10 (Darriba et al., 2012), under the AIC. We evaluated the convergence visually using Tracer v.1.7 (Rambaut et al., 2018) to plot probability scores for all parameters by generation time and to check the ESS (>200). A 10% burn-in was used. The Tree Annotator v.2.6.7 (available in the BEAST package) was used to summarize all nodes and the posterior distributions of each parameter in a maximum clade credibility (MCC) tree. The tree was visualized using the FigTree v.1.4.4 (Rambaut et al., 2018).

To obtain the later distribution of the estimated divergence times, nine calibration points were applied as normal priorities to restrict the age of the nodes, based on information on primate fossils: minimum age for the Simiiform Infrastructure of 43.5 ± 4.5 mya based on the fossil +*Darwinius masillae* (Franzen et al., 2009); minimum age for parvordem Platyrrhini of 23.5 ± 3.0 mya based on the fossil +*Dolichocebus gaimanensis* (Kay et al., 2008); minimum age

for the Hominidae family of 15.5 ± 2.5 mya (Matsui et al., 2009); minimum age for *Homo*-*Pan* separation of 6.5 ± 1.0 mya (Vignaud et al., 2002); minimum age for the subfamily Pitheciinae of 15.7 ± 2.0 mya, based on the fossil $^{+}$Proteropithecia (Kay et al., 1998); minimum age for the Atelinae subfamily at 12.6 ± 2.0 mya, based on the fossil $^{+}$Stirtonia (Kay et al., 1987); minimum age of the subfamily Aotinae at 12.5 ± 2.0 mya, based on the fossil $^{+}$*Aotus dindensis* (Kay and Fleagle, 2010); minimum age for the Callitrichinae subfamily at 13.4 ± 2.0 mya, based on $^{+}$Patasola and $^{+}$Lagonimico (Kay and Feagle, 2010); and a minimum age for the subfamily Cebinae based on $^{+}$Neosaimiri at 12.5 ± 2.0 mya (Rosenberger et al., 1991; Takai, 1994). To expand the dataset, ortholog sequences from the GenBank of 10 primates belonging to the parvordem Catarrhini were used as external groups: modern human (*Homo sapiens*), archaic human (Neanderthal*; Homo neanderthalensis*), common chimpanzee (*Pan troglodytes*), chimpanzee-pygmy (*Pan paniscus*), gorilla (*Gorilla gorilla*), orangutan (*Pongo abelii*), Rhesus monkey (*Macaca mulatta*), pig-tailed monkey (*Macaca nemestrima*), green monkey (*Chlorocebussabaeus*), and gelada baboon (*Theropitecus gelada*).

Species distribution modeling

We used Species Distribution Models (SDMs) for *Sapajus libidinosus*, *Sapajus nigritu*s, and eight plant species (*Astrocarium campestre, Attalea maripa, Attalea speciosa, Anacardium occidentale, Hymenaea martiana, Hymenaea stignocarpa, Manihot dichotoma,* and *Syagrus coronata*) accessed as food through the use of tools by *S. libidinosus* individuals. We also included *Ficus gomelleria*, which the Capuchin monkeys also consume. It is a juicy fig without a hard shell, so it does not require processing for consumption (http://servicos.jbrj.gov.br/flora/search/Ficus_gomelleira). We used both current and past climate data (Middle Holocene – Mid Hol: ~6,000 years ago (6 kya); Last Glacial Maximum (LGM): ~22 kya; Last Interglacial – LIG: ~140-120 kya) at 30″ spatial resolution (c. 1 km^2), based on bioclimatic variables from the WorldClim dataset: bio_1 = Annual Mean Temperature; bio_2 = Mean Diurnal Range [Mean of monthly (max temp - min temp)]; bio_3 = Isothermality (bio_2/bio_7) (×100);bio_4 = Temperature Seasonality (standard deviation ×100); bio_5 = Max Temperature of Warmest Month; bio_6 = Min Temperature of Coldest Month; bio_7 = Temperature Annual Range (bio_5-bio_6); bio_8 = Mean Temperature of Wettest Quarter; bio_9 = Mean Temperature of Driest Quarter; bio_10 = Mean Temperature of Warmest Quarter; bio_11 = Mean Temperature of Coldest Quarter; bio_12 = Annual Precipitation; bio_13 = Precipitation of Wettest Month; bio_14 = Precipitation of Driest Month; bio_15 = Precipitation Seasonality (Coefficient of Variation); bio_16 = Precipitation of Wettest Quarter; bio_17 = Precipitation of Driest Quarter; bio_18 = Precipitation of Warmest Quarter; bio_19 =

Precipitation of Coldest Quarter (Hijmans et al., 2005) (version 1.4). The three periods (Mid Hol, LGM, and LIG) are within the time (~200 kya) in which there is evidence of *Sapajus* species in the geographic region that is currently the Caatinga. The Mid Hol, LGM, and LIG climate data were derived from Community Climate System Model 4 (CCSM4). We retrieved the geospatial occurrences of monkeys and plant species from the Global Biodiversity Information Facility (www.gbif.org, 2021) and Link (www.macaulaylibrary.org, 2021). We also include location records from the literature (Falótico and Ottoni, 2013; Luncz et al., 2016) and unpublished field data collection by one of us (TF) Of the records obtained, we excluded those with inaccurate geographic location or localities in areas outside species' natural distribution. We also removed redundant geolocation within 20 km to minimize the overfit models caused by spatial sampling bias (Reddy and Davalos, 2003; Kramer-Schadt et al., 2013) As a result, 3,142 occurrences, considering the 11 investigated species (Tables S2-S11), were used in the niche modeling predictions.

We used a presence-only approach in Maxent v. 3.4.4 (Phillips et al., 2023) to first create SDMs of the present day and then project these models to the Middle Holocene, LGM, and LIG climates. We excluded correlated variables (Pearson's correlation coefficient $R^2 > 0.7$) among the 19 WorldClim variables to avoid overly complex models. To reduce over fitting and improve model performance, we optimized the regularization parameter testing for the optimal regularization value (1- 3- 5- 7- 9- 11) using ENMTools version 1.3 (Warren et al., 2010) (Table S12). Based on the optimized model parameters, we projected the models onto the Neotropical region (tropical America) (Antonelli and Sanmartín, 2011). We used five replicates for the species with less than 60 records and 10 replicates for species with more than 60 records. We used the "cross-validate" option and the jackknife data partitioning method to test and train the model. The model outputs shown are averages of these replicates, with pixels receiving a continuous output score between 0 and 1 indicating habitat suitability. Habitat suitability models were evaluated according to the acceptable area under the receiver-operator curve (ROC) values (AUC > 0.7) (DeLong et al., 1988; Pearce and Ferrier, 2000). In other words, values > 0.7 were considered acceptable for model discrimination.

Supplementary Material text and Supplementary Figure legends and Tables References

Anderson S and Yates TL (2000) A new genus and species of phyllotine rodent from Bolivia. J Mammal 81:18-36.

Antonelli A and Sanmartín I (2011) Why are there so many plant species in the Neotropics?. Taxon 60:403-414.

Beaumont MA (2010) Approximate Bayesian computation in evolution and ecology. *Annu* Rev Ecol Evol Syst, 41:379-406.

Bouckaert R, Vaughan TG, Barido-Sottani J, Duchêne S, Fourment M, Gavryushkina A, Heled J, Jones G, Kühnert D, De Maio N, et al. (2019) BEAST 2.5: An advanced software platform for Bayesian evolutionary analysis. PLoScomputationalbiology 15 e1006650.

Bradley RD, and Baker, RJ (2001) A test of the genetic species concept: cytochrome-b sequences and mammals. J Mammal, 82:960-973.

Brown WM, George Jr M, and Wilson AC (1979) Rapid evolution of animal mitochondrial DNA. Proc Natl Acad Sci, 76:1967-1971.

Canale GR, Guidorizzi, CE, Kierulff, MCM and Gatto CAFR (2009) First record of tool use by wild populations of the yellow breasted capuchin monkey (Cebus xanthosternos) and new records for the bearded capuchin (Cebus libidinosus) Am J Primatol, 71:366–372.

Cao LZ, and Wu KM (2019) Genetic diversity and demographic history of globe skimmers (Odonata: Libellulidae) in China based on microsatellite and mitochondrial DNA markers. Sci Rep, 9:8619.

Coelho CG, Falótico T, Izar P, Mannu M, Resende BD, Siqueira JO, and Ottoni EB (2015) Social learning strategies for nut-cracking by tufted capuchin monkeys (Sapajus spp.) Anim Cogn, 18, 911-919.

Cornuet JM, Pudlo P, Veyssier J, Dehne-Garcia A, Gautier M, Leblois R, Marin JM and Estoup, A. (2014) DIYABC v2. 0: a software to make approximate Bayesian computation inferences about population history using single nucleotide polymorphism, DNA sequence and microsatellite data. Bioinform, 30:, 1187-1189.

Cornuet JM, Santos F, Beaumont MA, Robert CP, Marin JM, Balding DJ, Guillemaud T and Estoup A (2008) Inferring population history with DIY ABC: a user-friendly approach to approximate Bayesian computation. Bioinform, 24: 2713-2719.

Darriba D, Taboada GL, Doallo R, and Posada D (2012) jModelTest 2: more models, new heuristics and parallel computing. Nat Methods 9:772-772.

DeLong ER, DeLong DM, and Clarke-Pearson DL (1988) Comparing the areas under two or more correlated receiver operating characteristic curves: a nonparametric approach. Biometrics, 837-845.

Drummond AJ, Rambaut A, Shapiro BETH, and Pybus OG (2005) Bayesian coalescent inference of past population dynamics from molecular sequences. Mol Biol Evol, 22: 1185-1192.

Excoffier L, and Lischer HE (2010) Arlequin suite ver 3.5: a new series of programs to perform population genetics analyses under Linux and Windows. Mol Ecol Resour, 10:564-567.

Falótico T, and Ottoni EB (2013) Stone throwing as a sexual display in wild female bearded capuchin monkeys, *Sapajus libidinosus*. PLoS One, 8: e79535.

Falótico T, and Ottoni EB (2014) Sexual bias in probe tool manufacture and use by wild bearded capuchin monkeys. Behav Processes, 108:117-122.

Falótico T, and Ottoni EB (2016) The manifold use of pounding stone tools by wild capuchin monkeys of Serra da Capivara National Park, Brazil. Behav, 153:421-442.

Falótico T, Valença T, Verderane MP, Santana BC, and Sirianni G. 2023. Mapping nut-cracking in a new population of wild capuchin monkeys (*Sapajus libidinosus*) at Ubajara National Park, Brazil. Am J Primatol (accepted)

Felsenstein J (1993) phylip (phylogeny inference package), version 3.5c. Department of Genetics, University of Washington, Seattle.

Ferreira RC (2004) Coalitions and social dynamics of a semi-free ranging Cebus apella group (Doctoral dissertation, University of Cambridge)

Franzen JL, Gingerich PD, Habersetzer J, Hurum JH, Von Koenigswald W, and Smith BH (2009) Complete primate skeleton from the middle Eocene of Messel in Germany: morphology and paleobiology. PLoS One, 4:e5723.

Gage TB (1998) The comparative demography of primates: with some comments on the evolution of life histories. Ann Rev Anthropol, 27:197-221.

Irwin DM, Kocher TD, and Wilson AC (1991) Evolution of the cytochrome *b* gene of mammals. J Mol Evol, 32:128-144.

Izar P, Ferreira RG, and Sato T (2006) Describing the organization of dominance relationships by dominance-directed tree method. Am J Primatol, 68:189-207.

Kay RF., and Fleagle JG (2010) Stem taxa, homoplasy, long lineages, and the phylogenetic position of Dolichocebus. J Hum Evol, 59, 218-222.

Kay RF, Fleagle JG, Mitchell TRT, Colbert M, Bown T and Powers DW (2008) The anatomy of Dolichocebusgaimanensis, a stem platyrrhine monkey from Argentina. J Hum Evol, 54:323-382.

Kay RF, Johnson D and Meldrum DJ (1998) A new pitheciin primate from the middle Miocene of Argentina. Am J Primatol, 45:317-336.

Kay RF, Madden RH, Plavcan JM, Cifelli, RL and Díaz JG (1987) Stirtoniavictoriae, a new species of Miocene Colombian primate. J Hum Evol, 16:173-196.

Kramer-Schadt S, Niedballa J, Pilgrim JD, Schröder B, Lindenborn J, Reinfelder V, Stillfries M, Heckmann I, Scharf A and Wilting, A. (2013) The importance of correcting for sampling bias in MaxEnt species distribution models. Divers Distrib, 19:1366-1379.

Kuhner MK (2006) LAMARC 2.0: maximum likelihood and Bayesian estimation of population parameters. Bioinform, 22:768-770.

Kumar R, Pandey BK, Sarkar UK, Nagpure NS, Baisvar VS, Agnihotri P, Awasthi A, Mishra A and Kumar, N. (2017) Population genetic structure and geographic differentiation in butter catfish, Ompokbimaculatus, from Indian waters inferred by cytochrome b mitochondrial gene. Mitochondrial DNA A, 28:442-450.

Larsson A (2014) AliView: a fast and lightweight alignment viewer and editor for large datasets. Bioinform, 30:3276-3278.

Lima MG, Buckner JC, Silva-Júnior JDSE, Aleixo A, Martins AB, Boubli JP, Link A, Farias IP, da Silva MN, Röhe F. et al (2017). Capuchin monkey biogeography: understanding Sapajus Pleistocene range expansion and the current sympatry between Cebus and Sapajus. J Biogeogr 44:810-820.

Luncz LV, Falótico T, Pascual-Garrido A, Corat C, Mosley H, and Haslam M (2016) Wild capuchin monkeys adjust stone tools according to changing nut properties. Sci Rep, 6: 33089.

Lynch Alfaro JW, Boubli JP, Olson LE, Di Fiore A, Wilson B, Gutiérrez-Espeleta GA, Chiou KL, Schulte M, Neitzel S, Alfaro, ME et al. (2012) Explosive Pleistocene range expansion leads to widespread Amazonian sympatry between robust and gracile capuchin monkeys. Journal of biogeography, 39:272-288.

Lynch Alfaro JW, Boubli JP, Paim, FP, Ribas CC, da Silva MNF, Messias MR, Röhe F, Mercês MP, Silva Júnior JS, Silva CR et al. (2015) Biogeography of squirrel monkeys (genus Saimiri): South-central Amazon origin and rapid pan-Amazonian diversification of a lowland primate. Mol Phylogenet Evol 82:436-454.

Martins AB, Fialho MS, Jerusalinsky L, Valença-Montenegro MM, Bezerra BM, Laroque PO and Lynch Alfaro, JW (2021) *Sapajus libidinosus* (amended version of 2019 assessment) IUCN Red List Threat. Species, e-T136346A192593226.

Martins AB, Valença-Montenegro MM, Lima MGM, Lynch JW, Svoboda WK, Silva-Júnior JDSE, Röhe F, Boubli JP and Fiore AD (2023) New assessment of robust capuchin monkey (*Sapajus*) evolutionary history using genome-wide SNP marker data and a bayesian approach to species delimitation. Genes 14:970.

Martins-Junior AMG, Carneiro J, Sampaio I, Ferrari SF and Schneider H (2018) Phylogenetic relationships among Capuchin (Cebidae, Platyrrhini) lineages: An old event of sympatry explains the current distribution of Cebus and Sapajus. Genet Mol Biol 41:699-712.

Matsui A, Rakotondraparany F, Munechika I, Hasegawa M, and Horai S. (2009) Molecular phylogeny and evolution of prosimians based on complete sequences of mitochondrial DNAs. Gene, 441:53-66.

Moraes B, Razgour O, Souza-Alves JP, Boubli JP, and Bezerra B. (2020) Habitat suitability for primate conservation in north-east Brazil. Oryx, 54:803-813.

Ottoni EB and Izar P (2008) Capuchin monkey tool use: overview and implications. Evol Anthropol, 17:171-178.

Ottoni EB, and Mannu M (2001) Semifree-ranging tufted capuchins (Cebus apella) spontaneously use tools to crack open nuts. Int J Primatol 22:347-358.

Pearce J, and Ferrier S. (2000) Evaluating the predictive performance of habitat models developed using logistic regression. Ecol Modell, 133:225-245.

Perelman, P, Johnson WE, Roos C, Seuánez HN, Horvath JE, Moreira MA, Kessing B, Pontius J, Roelke M, Rumpler Y et al. (2011) A molecular phylogeny of living primates. PLoS Genet 7:e1001342.

Phillips SJ, Dudík M, Schapire RE (2023) Maxent software for modeling species niches and distributions (Version 3.4.4) [accessed 14 December 2022]. http://biodiversityinformatics.amnh.org/open_source/maxent/.

Presotto A, Remillard C, Spagnoletti N, Salmi R, Verderane M, Stafford K Izar, P. (2020) Rare bearded capuchin (Sapajus libidinosus) tool-use culture is threatened by land use changes in northeastern Brazil. Int J Primatol, 41, 596-613.

Rambaut A (2018) FigTree v.1.4.4. [accessed 10 March 2023]. http://tree.bio.ed.ac.uk/software/figtree/

Rambaut A, Drummond AJ, Xie D, Baele G, and Suchard MA (2018) Posterior summarization in Bayesian phylogenetics using Tracer 1.7. Syst Biol, 67:901-904.

Reddy S, and Dávalos, LM (2003) Geographical sampling bias and its implications for conservation priorities in Africa. J Biogeogr, 30:1719-1727.

Rosenberger AL, Hartwig WC, Takai M, Setoguchi T, and Shigehara N (1991) Dental variability in Saimiri and the taxonomic status of Neosaimirifieldsi, an early squirrel monkey from La Venta, Colombia. Int J Primatol, 12:291-301.

Rozas J, Ferrer-Mata A, Sánchez-DelBarrio, JC, Guirao-Rico S, Librado P, Ramos-Onsins, SE, and Sánchez-Gracia A (2017) DnaSP 6: DNA sequence polymorphism analysis of large data sets. Mol Biol Evol, 34, 3299-3302.

Salzburger W, Ewing GB, and Von Haeseler A (2011) The performance of phylogenetic algorithms in estimating haplotype genealogies with migration. Molecular ecology, 20:1952-1963.

Schrago CG, and Mello B (2020) Employing statistical learning to derive species-level genetic diversity for mammalian species. Mamm Rev, 50, 240-251.

Takai M (1994) New specimens of Neosaimirifieldsi from La Venta, Colombia: a middle Miocene ancestor of the living squirrel monkeys. J Hum Evol 27, 329-360.

Tobe SS, Kitchener AC, and Linacre AM (2010) Reconstructing mammalian phylogenies: a detailed comparison of the cytochrome *b* and cytochrome oxidase subunit I mitochondrial genes. PloS One, 5: e14156.

Vignaud P, Duringer P, Mackaye HT, Likius A, Blondel C, Boisserie JR, Bonis L, Eisenmann V, Etienne ME, Brunet M et al. (2002) Geology and palaeontology of the Upper Miocene Toros-Menalla hominid locality, Chad. Nature, 418, 152-155.

Warren DL, Glor, RE, and Turelli M. (2010) ENMTools: a toolbox for comparative studies of environmental niche models. Ecography, 33:607-611.

Watterson GA. (1975) On the number of segregating sites in genetical models without recombination. Theor Popul Biol, 7: 256-276.
